# Supplementary material for: GRAYU: graph-based database integrating Ayurvedic formulations, medicinal plants, phytochemicals and diseases
Source: Front Pharmacol. 2026 Jan 22;16:1727224. doi: 10.3389/fphar.2025.1727224 (PMC12872832; doi:10.3389/fphar.2025.1727224)
Supplement: Supplementary file 1 [file Supplementaryfile1.docx]

*Supplementary Table 1: List of Ayurvedic terms and their descriptions adapted from Ayurvedic Standard Treatment Guidelines and Ayurvedic Pharmacopeia.*

| **Ayurvedic Term** | **Description** |
| --- | --- |
| Aadharniya vega | Non-suppression of natural urges It means the natural urges which should not be suppressed by force as a habit. |
| Abhighatabhicari Jvara | Fever due to physical or psychological trauma |
| Abhinyasa Jvara | Meningitis |
| Abhisyanda | Conjunctivitis |
| Abhyanga | An Ayurvedic oil massage practice. This helps loosen and facilitate the removal of accumulated Ama (toxins) and the Doshas (Vata, Pitta and Kapha) from the body. |
| Adhimantha | Glaucoma |
| Adhmana | Flatulance with gurgling sound |
| Adhyavata | Gout |
| Adrsti | Loss of eyesight |
| Agantuka | External factor |
| Aghata | Trauma |
| Agni | Agni is the form of fire and heat that is the basis of the digestive system and the process of release of energy. The term includes the body heat, body temperature, sight, the digestive fire; its function is transformation, absorption, elimination and discrimination is Agni. |
| Agni Vikrti | Impaired digestion |
| Agnidagdha | Burn injury |
| Agnimandya | Digestive impairment |
| Agnivikara | Diseases due to vitiation of Agni |
| Ahara | Food articles used by human |
| Ahara Rasa | It is the nutrient substance developed after digestion process taken in the digestive tract where enzymes or the bile juice acted upon food articles |
| Ajirna | Dyspepsia |
| Ajirna/ Ajeerna | Indigestion, weak digestion |
| Aksepa | Convulsions |
| Aksiroga | Eye disease |
| Alasaka | Intestinal atony |
| Alasya | Laziness |
| All kinds of Slipada | Filariasis |
| All Pittaroga | All diseases due to Pitta dosa |
| All types of Jvara | Fever |
| All varieties of Sotha | Inflammation |
| Ama | Toxins or Ama is produced in the body by the raw, undigested food products that become fetid. |
| Amadosa | Products of impaired digestion and metabolism / consequences of Ama |
| Amagrahani | Sprue associated with indigestion |
| Amaja sula | Intestinal colic due to indigestion |
| Amajirna | Indigestion due to Ama |
| Amajvara | Fever due to indigestion |
| Amasula | Colicky Pain due to Ama |
| Amatisara | Diarrhoea due to indigestion/ Ama |
| Amavata | Rheumatism |
| Amla | Sour taste |
| Amladaha | Hyperacidity |
| Amlapitta | Dyspepsia |
| Amsaphalaka Sula | Pain in Scapular region |
| Amsatapa | Burning sensation in scapular region |
| Anaha | Distension of abdomen due to obstruction to passage of urine and stools |
| Anda Sula | Testicular pain |
| Andhatva | Blindness |
| Angadaha | Burning sensation all over the body |
| Angamarda | Body ache |
| Angastambha | Body stiffness |
| Anidra | Insomnia |
| Anna-vaha srotas | The channels transporting grains or food, the digestive system, alimentary canal. |
| Antardaha | Internal Burning sensation |
| Antarvidradhi | Internal abscess |
| Antarvidradhi | Internal abscess |
| Antra Vrddhi | Hernia |
| Antrasula | Intestinal colic |
| Anupana | Vehicle for medicine |
| Anuvasana (Basti) | Enema given with an oily substance |
| Apaci | Chronic lymphadenopathy / scrofula |
| Apana | One of the five types of Vata, which goes downward and is responsible for expulsion of faces, flatus, urine, menstrual blood etc. |
| Apana Vayu Nirodha | Obstruction of flatus |
| Apasmara | Epilepsy |
| Apatanaka | Tetanic convulsions |
| Apatantraka | Hysteria with loss of consciousness |
| Arbuda | Tumor |
| Ardhavabhedaka | Migraine |
| Ardita | Facial palsy |
| Arditavata | Facial palsy |
| Arma | Pterygium |
| Arocaka | Tastelessness |
| Arsa | Haemorrhoids |
| Arsoroga | Haemorrhoids |
| Artava | Menstrual blood |
| Artava Ruja | Dysmenorrhoea |
| Artava Vedana | Dysmenorrhoea |
| Artavadosa | Vitiation of Artava |
| Artavavaha Srotas | The channel that carries menstrual fluid and ovum; consists of female reproductive system i.e. fallopian tubes, ovaries, uterus and vaginal canal |
| Aruci | Tastelessness |
| Asatmya | Unwholesome, bad, improper. |
| Aschyotana | Type of eye treatment in which drops of herbal liquids are put into the eyes. |
| Asmari | Calculus |
| Asmariksaya | Urinary calculus |
| Aspastabhasana | Incoherent speech |
| Asrgdara | Menorrhagia or Metrorrhagia or both |
| Asrgdara Ruja | Dysmenorrhoea |
| Asta Jvara | Eight types of Fever |
| Asthi Bhagna | Bone fracture |
| Asthi Bhanga | Bone fracture |
| Asthi Cyuti | Dislocation of bones, joints |
| Asthi Ruja | Ostealgia |
| Asthi Strava | Discharge from bones |
| Asthicyuta | Dislocation of bones, joints |
| Asthigata Vata | Vata confined to bones |
| Asthila | Prostatic Hyperplasia |
| Asthisosa | Osteoporosis |
| Asthivata | Bone diseases due to Vata dosa |
| Asya Roga | Diseases of buccal cavity |
| Atisara | Diarrhoea |
| Atopa | Gurgling found in abdomen with pain |
| Audumbara Kustha | Patchy leprosy / lepromatous leprosy |
| Avabahuka | Brachialgia |
| Avrana Sukra | Corneal opacity |
| Ayama | Stretching |
| Ayu | Life |
| Baddhapurisa | Hard stools |
| Badhiratva | Deafness |
| Badhirya | Deafness |
| Bahu Sosa | Muscular wasting of forearm |
| Bahumutra | Polyuria |
| Bahusula | Pain in arm |
| Bahyadaha | Burning sensation |
| Bahyayama | Opisthotonos |
| Bala | Strength |
| Bala Graha | Specific disorders of children |
| Bala Roga | Diseases of children |
| Bala Sosa | Emaciation in children |
| Balaksaya | Loss of strength / immunity |
| Balavarna Ksaya | Loss of physical strength and complexion |
| Balya | An energizer that gives strength to the body |
| Basti | It refers to the enema therapy under Panchakarma. Basti is the most effective treatment of Vata disorder. In Ayurveda Basti involves the introduction of herbal solution and oil preparations in the rectum. |
| Bastigataroga | Disorders of urinary system |
| Bastiroga | Diseases of urinary system |
| Bastiruja | Pain in urinary system |
| Bhagandara | Fistula-in-ano |
| Bhasma | Substance obtained by calcination |
| Bhasmaka | Polyphagia with emaciation |
| Bhrama | Vertigo |
| Bhru-Sankha-Karnasula | Trigeminal neuralgia |
| Bhuta Badha | Psychological disorder |
| Bhuta Visa | Microbial infection |
| Bhutonmada | Exogenous psychosis |
| Bradhna Vrddhi | Hernia |
| Buddhi Bhrama | Dementia |
| Buddhi Daurbalya | Weak intelligence |
| Buddhiksaya | Impaired intelligence |
| Buddhimandata | Low intelligence |
| Buddhimandya | Low intelligence |
| Caladanta | Loose tooth |
| Carmadala | Exfoliative dermatosis |
| Carmaroga | Skin disorders |
| Chakhyushya | Substances that are good for eyes |
| Chardi | Emesis |
| Chikitsa | Treatment, a therapy to retain balance of Doshas, practice or science of medicine. |
| Churna / Curna | Powder |
| Dadru | Taeniasis |
| Dadrukustha | Taeniasis |
| Dagdha Vrana | Burn ulcer |
| Daha | Burning sensation |
| Damsa Vrana | Ulcer due to bites |
| Damstra Visa | Poisonous bite |
| Dandapatanaka | Tetanus / Plenosthotonus |
| Danta Calana | Loose tooth |
| Danta Krmi | Dental caries |
| Danta Roga | Disease of tooth |
| Danta sula | Dental Pain |
| Danta-Nakha Ksata | Injury caused by teeth and nails |
| Dantabandha | Lock jaw |
| Dantapida | Pain in Teeth |
| Dantaroga | Dental disease |
| Dantasausirya | Dental caries |
| Dantodbhava Jvara | Dentitional fever |
| Dantodbhava Kalina Jvara | Fever during dentation |
| Daruna Visucika | Severe gastro enteritis |
| Daurbalya | Weakness |
| Deepaniya | Natural substances that kindle the gastric fire and augment  the appetite |
| Dehasithilata | Unenergetic body |
| Dehasula | Bodyache |
| Dhanurvata | Tetanus / Plenosthotonus |
| Dhatu | Basic structural and nutritional body factor that supports or nourishes the seven body tissues. These seven tissues of our body includes the rasa, Rakta, Mamsa, Meda, Asthi, Majja and Shukra. |
| Dhatu Ksaya | Tissue wasting |
| Dhatugata Atisara | Diarrhoea resulting in tissue wasting |
| Dhatusosa | Cachexia |
| Dhatustha Jvara | Fever due to dosa residing in particular Dhatu |
| Dhvajabhanga | Failure of penile erection |
| Dinacharya | Daily routine to be followed in day to day practice |
| Drsti | Sight |
| Drsti Daurbalya | Weakness of vision |
| Drsti Lopa | Defects in eye sight |
| Drsti Lopa | Defects in eye sight |
| Duhkha Prasava | Difficult labour |
| Duradrsti | Hypermetropia / Hyperopia |
| Dushti | Unhappiness, imbalanced, improper functioning |
| Dusta Vrana | Non-healing ulcer |
| Dustanadivrana | Infected/ non healing fistula |
| Dustapinasa | Infected/ non responding rhinitis |
| Ekanga Sosa | Wasting of one limb |
| Enhances Bala | Strength / Immunity |
| Enhances Bala, Varna, Ayu | promotes Physical strength, Complexion and Longevity |
| For external use only as Abhyanga | (Massage) |
| Gadhavitkata | Constipation |
| Gala Roga | Diseases of throat |
| Galaganda | Goiter |
| Galagraha | Difficulty in swallowing |
| Galasosa | Dryness in throat |
| Gambhira Vrana | Deep ulcer |
| Ganda | Maxillar prominence |
| Gandamala | Cervical lymphadenitis |
| Gandupada Krmi Roga | Ascariasis |
| Gara | Slow / accumulated poison |
| Gara Visa | Slow / accumulated poison |
| Gara-Udararoga | Abdominal disorder due to slow / accumulated poison |
| Garadosa | Effects of slow / accumulated poison |
| Garbhadosa | Foetal anomaly |
| Garbhapata | Abortion |
| Garbhasaya Dosa | Uterine disorder |
| Garbhasayacyuti | Uterine prolapse |
| Garbhasayaroga | Disorders of Uterus |
| Garbhasrava | Threatened abortion |
| Garbhini Daha | Burning sensation during pregnancy |
| Garbhini Jvara | Pyrexia during pregnancy |
| Garbhini Roga | Disorders during pregnancy |
| Garbhini Roga | Disorders during Pregnancy |
| Gatra Kampa | Tremors |
| Gatra Kandu | Generalised pruritus |
| Gatra Saitya | Cold calm extremities |
| Gatrasosa | Wasting of limbs |
| Gatrasula | Bodyache |
| Ghrita (Ghee) | Clarified butter made by heating unsalted butter from cow especially |
| Given as Rasayana and Vajikarana also | Nutrient to body and mind with adapto-immuno-neuro-endocrino-modulator properties and Aphrodisiac |
| Graha Dosa | Psychosis |
| Grahani | Malabsorption syndrome |
| Grahani Dosa | Disorders of small intestine |
| Grahani Roga | Malabsorption syndrome |
| Graharoga | Psychosis |
| Granthi | Cyst |
| Granthiruk | Pain in cyst |
| Grdhrasi | Sciatica |
| Griva Stambha | Neck Stiffness |
| Griva suskata | Wasting in cervical region |
| Guda Nihsarana | Rectal prolapse |
| Guda Roga | Anorectal disease |
| Guda Ruja | Pain in the anorectal area |
| Guda Sopha | Swelling in the anorectal area |
| Guda Sotha | Inflammation of the rectum |
| Guda Sula | Pain in the anorectal area |
| Gudabhramsa | Prolapse of the rectum |
| Gudankura (Arsa) | Piles |
| Guhyavrana | Ulcer in genitalia |
| Gulma | Abdominal lump |
| Halimaka | Chronic obstructive Jaundice/Chlorosis/Advanced stage of Jaundice |
| Hanugraha | Lock jaw |
| Hanusopha | Swollen Jaw |
| Hanustambha | Lock jaw |
| Hasta Pada Daha | Burning sensation in palms and soles |
| Hastakampa | Tremors in hands |
| Hastapada Roga | Disease of the limbs |
| Hidhma | Hiccup |
| Hikka | Hiccup |
| Hina yoga | Insufficient, deficient |
| Hinamamsa | Lack of muscle tissue |
| Hrcchula | Angina pectoris |
| Hrdayadaurbalya | Weakness of heart |
| Hrdayagra Vrdhhi | Ventricular Hypertrophy |
| Hrdayasula | Angina pectoris |
| Hrddaha | Burning sensation in heart region |
| Hrddaurbalya | Weakness of the heart |
| Hrdroga | Heart disease |
| Hrdruja | Angina pectoris |
| Hrllasa | Nausea |
| Hrtgraha | Cardiac failure |
| Hrtkampa | Cardiac fibrillation |
| Hrtsula | Angina pectoris |
| Improves Bala | Strength / Immunity |
| Improves Bala, Varna, Drsti | Improves strength, complexion, vision power |
| Improves Bala, Vrsyasakti, Mamsa | i.e. physical strength, sexual vigour and muscle mass |
| Improves Bhuja bala | Improves Physical Strength |
| Improves Netra drsti | Eyesite |
| Improves Vak | Speech |
| Indralupta | Alopecia |
| It is a Hrdyavirecaka | laxative |
| Jala | Water |
| Jalamrta | Drowning |
| Jalauka | Leech |
| Jalodara | Ascites |
| Jangha Janugata Vata | Pain in calf and knee |
| Jangha Ruja | Pain in thigh |
| Jangha Sopha | Swelling in the thigh |
| Janghasula | Pain in calves |
| Janghoru-Prsthatrikasthana and Vastigatasula | Pain in calves-thighs-back-sacral and bladder region |
| Janu Stabdhata | Stiffness of the knee |
| Janusula | Pain in the knee |
| Jara | Senility/Progeriasis |
| Jaradosa | Senility |
| Jarayu Dosa | Vitiation of placenta |
| Jathara | Stomoch |
| Jathara | Stomach, belly or abdomen |
| Jatharagni | Digestive fire |
| Jatharagni | Fire located in stomach, digestive fire, gastric juices, digestive enzymes |
| Jatumani | Congenital mole |
| Jihvastambha | Glossal palsy |
| Jirna Kasa | Chronic cough |
| Jirnajvara | Chronic fever |
| Jvara | Fever |
| Jvaratisara | Diarrhoea with fever |
| Kaca | Cataract |
| Kacchu | Itching |
| Kala | Time, period, season |
| Kalka | Paste of herbs to be used for medicinal purposes |
| Kamala | Jaundice |
| Kampa | Tremor |
| Kandu | Itching |
| Kantha Roga | Disease of throat |
| Kanthabandha | Feeling of bandage around neck |
| Kanthadaha | Burning sensation in throat |
| Kantharoga | Disorders of Throat |
| Kapha | It is one of the three Doshas i.e. the water humour, the intracellular fluid and the extra cellular fluid that plays significant role in the nutrition and existence of body cells and tissues |
| Kapha Jvara | Fever due to Kapha dosa |
| Kaphadosa | Vitiation of Kapha dosa |
| Kaphaja Agnimandya | Impaired digestive fire due to diminished agni |
| Kaphaja Hrdroga | Heart disease due to Kapha dosa |
| Kaphaja Kasa | Cough due to Kapha dosa |
| Kaphaja Roga | Disease due to Kapha dosa |
| Kaphaja sula | Pain due to Kapha dosa |
| Kaphajasandhibandha | Stiffness in joints due to Kapha dosa |
| Kaphapitta | Disease due to Kapha dosa and Pitta dosa |
| Kaphapitta Kustha | Skin disease caused by Kapha and Pitta dosa |
| Kaphapittajasula | Pain due to Kapha and Pitta dosa |
| Kaphapittaroga | Disease due to Kapha dosa and Pitta dosa |
| Kaphapraseka | Excessive salivation |
| Kapharoga | Disease due to Kapha dosa |
| Kaphavataja Nadi Vrana. | Fistula due to Kapha, Vata |
| Kaphavataroga | Disease due to Kapha and Vata dosa |
| Kaphavikara | Disorders due to vitiation of Kapha dosa |
| Kaphonmada | Psychosis due to Kapha dosa |
| Kaphotklesa | Nausea |
| Karna Krmi | Worm infestation in Ear |
| Karna Roga | Disease of ear |
| Karna Strava | Otorrhoea |
| Karnagata Nadivrana | Fistula in Ear |
| Karnanada | Tinnitus |
| Karnapaka | Otitis externa diffusa |
| Karnaruja | Otalgia |
| Karnasula | Otalgia |
| Karsya | Emaciation |
| Kasa | Cough |
| Kashaya | Astringent taste or flavour; sometimes use for decoction of herbs |
| Kastartava | Dysmenorrhoea |
| Kati Graha | Stiffness in lumbo-sacral region |
| Kati sula | Lower backache |
| Kati-uru-Prstha Daurbalya | Weaknes and lower backache radiating to thighs |
| Katiruja | Backache |
| Katistambha | Restricted movement of the lumbo-sacral region |
| Katiuruprstha Daurbalya | Weaknes and lower backache radiating to thighs |
| Kativata | Disorders of lumbo-sacral region due to vitiated Vata dosa |
| Katu | Pungent taste or flavour |
| Kaubja | Hump/Kyphosis |
| Kaumarabhrtya | The branch of Ayurveda that deals with child health (Paediatrics) |
| Kayachikitsa | Internal Medicine, treatment of body diseases, |
| Kesa Patana | Falling of hair |
| Kesapata | Falling of hair |
| Kesasata | Loss of hair |
| Khalitya | Alopecia |
| Khanja | Limping |
| Khanja Vata | Limping |
| Khudaka | Limping |
| Kilasa | Vitiligo |
| Kitibha | Depigmentation |
| Klama | Tiredness without exertion/Langour |
| Kledi Vrana | Oozing/weeping ulcer |
| Kostha sula | Pain in abdomen |
| Kostha Vikara | Disorders of abdomen |
| Kostharuja | Pain in abdomen |
| Kotha | Urticaria |
| Krcchrartava | Dysmenorrhoea |
| Kriyakalpa | Medical procedures used in eye treatment |
| Krmi | Helminthiasis/Worm infestation |
| Krmi Roga | Worm infestation |
| Krmija Hrdroga | Bacterial endocarditis |
| Krostuka Sirsa | Synovitis of Knee joint |
| Krsata | Emaciation/Cachexia |
| Krsatva | Emaciation |
| Krtrima Visa | Synthetic poison |
| Ksata | Wound |
| Ksata Ksaya | Emaciation due to injury |
| Ksata Ksina | Debility due to chest injury |
| Ksataja Ksaya | Emaciation due to injury |
| Ksaya | Pthisis |
| Ksayaja Kasa | Cough due to Pthisis |
| Kshara | Alkali preparations of herbs |
| Ksina Retas | Oligospermia |
| Ksina sukra | Oligospermia |
| Ksinendriya | Impaired senses |
| Ksudra Kustha | Group of minor skin diseases |
| Kubja | Hump-back/Kyphosis |
| Kubja Roga | Hump-back/Kyphosis |
| Kubja Vata | Kyphosis |
| Kubjata | Dwarfism |
| Kuksi Ruja | Pelvic pain |
| Kuksisula | Pelvic pain |
| Kukunaka | Ophthalmia neonatorum |
| Kumbhakamala | Hepatitis |
| Kustha | Diseases of skin |
| Laghu | Light, small, minute |
| Lalajihvata | Excess salivation |
| Langhana | Depletion therapy, slimming therapy makes body thin and light |
| Lavana | Salty taste of flavour |
| Lekhaniya | Substances that have scraping actions on body tissues |
| Luta Visa | Poison of Spider |
| Luta-Vrscika-Sarpavisa | Poison of Scorpian, Spider and Snake |
| Mada | Intoxication |
| Madatyaya | Alcoholism |
| Madhu | Honey |
| Madhumeha | Diabetes mellitus |
| Madhura | Sweet taste of flavour, pleasant, charming, delightful |
| Madyapanaja Vikara | Ailments due to Intoxication of Fermented liquids |
| Mahakustha | Group of major skin diseases |
| Mahatapa | Severe body temperature |
| Mahavata Roga | Major neurological disorder |
| Majja | Bone marrow |
| Majjagata Vata | Bone marrow related disorder |
| Majjavata | Bone marrow disorder |
| Mala | Waste products that are to be excreted out of the body. It primarily includes urine, faeces and sweat |
| Malabandha | Constipation |
| Malasodhaka | Laxative |
| Mamsa | Muscle |
| Mamsa | Muscles and related systems in body |
| Mamsa Ksaya | Muscle Atrophy |
| Mamsa Vrddhi | Muscle Hypertrophy |
| Mamsavaha Srotas | Channels transporting to the muscles and related tissues |
| Mana | Mind |
| Manasa Dosa | Mental disorders |
| Mandabuddhitva | Retarded intellect |
| Mandadrsti | Diminished vision |
| Mandagni | Impaired digestive fire |
| Mandajvara | Low grade fever |
| Mandala Kustha | Lepromatous lesion |
| Mandala Vrana | Ulcers |
| Manodaurbalya | Mental weakness |
| Manodosa | Mental disorder |
| Manoglani | Depression |
| Manoroga | Psychological Disorder |
| Manovaha Srotas | The channel that carries thought, feelings and emotions; refers to the entire mind |
| Manovibhrama | Delirium |
| Manovikara | Mental disorder |
| Manya Stambha | Neck rigidity/Torticollis |
| Manyaroga | Diseases of Neck |
| Manyastambha | Neck rigidity/Torticollis |
| Marmasritavrana | Ulcers in vital points |
| Marmavata | Vitiation of Vata in Vital points |
| Marmavikara | Disorders of vital points |
| Masaka | Mole |
| Mastaka Bhrama | Vertigo |
| Mastiskaroga | Disorders of Brain |
| Masurikajvara | Eruptive fever |
| Meda | Adipose tissue |
| Meda | It is the fat tissue supported by Mamsa Dhatu |
| Medha | Intelligence |
| Medhalpata | Retarded intelligence |
| Medhraroga | Penile diseases |
| Medhya | Brain tonic/nootropic |
| Medhya | That which enhances wisdom, mental power and intelligence |
| Medodosa | Disorder of adipose tissue |
| Medogatavata | Disease of adipose tissue affected by vitiated Vata |
| Medoroga | Obesity |
| Medovaha Srotas | Channels transporting to fat and related tissues |
| Medovikara | Disordes of Fat |
| Medovrddhi | Obesity |
| Meha | Excessive flow of urine |
| Mithyaayoga | Wrong use, wrong employment |
| Moha | Delusion |
| Mootra /Mutra | Urine |
| Moudhyam | Stupidity |
| Mudha Garbha | Malpresentation of the foetus |
| Mudha Vata | Obstructed movement of Vata dosa |
| Mudhata | Entrapment of Vata |
| Mukata | Aphasia |
| Mukha Daurgandhya | Halitosis |
| Mukha Paka | Stomatitis |
| Mukha Roga | Disease of mouth |
| Mukha Roga (Kavala) | Disease of mouth |
| Mukha Sosa | Dryness in mouth / buccal cavity |
| Mukha-Karna-Nasa-Aksi Vikrti | Disorders of Mouth, Ear, Nose and Eyes |
| Mukhadurgandha | Halitosis |
| Mukhadurgandhya | Halitosis |
| Mukhajadya | Restricted movement of jaw |
| Mukhapaka | Mouth ulcer |
| Mula Visa | Poisoning due to roots of plants |
| Murcha | Syncope |
| Murcha Kampa | Tremors due to syncope |
| Musika Visa | Rat poisoning |
| Mutra Sarkara | Urinary calculus |
| Mutra Vibandha | Retention of urine |
| Mutrabandha | Retention of urine |
| Mutradosa | Urinary disorders |
| Mutraghata | Urinary obstruction |
| Mutrakrcchra | Dysuria |
| Mutraroga | Urinary diseases |
| Mutrasada | Oligouria |
| Mutrasanga | Obstruction in urinary tract |
| Mutratisara | Polyurea |
| Mutravaha Srotas | Channels transporting to the urinary system |
| Mutravirecanartha | Diuresis |
| Nabhi Sula | Pain in umbilical region |
| Nadi | Pulse, any tubular organ such as vein or artery |
| Nadi Vrana | Wounds with sinuses |
| Nadivrana | Fistula |
| Naktandhya | Night blindness |
| Naktandhya | Night blindness |
| Napumsakata | Impotency |
| Nasa Roga | Disease of nose |
| Nasikadurgandha | Ozena |
| Nasta Puspa | Amenorrhoea |
| Nastasukra | Oligospermia/ Azoospermia |
| Nasya | Herbal medication through nasal |
| Navajvara | Acute fever |
| Nayana Roga | Disease of the eye |
| Netra | Eye |
| Netra Adhimamsa | Pterygium |
| Netra Daha | Burning sensation in eyes |
| Netra Kandu | Itching in eyes |
| Netra Ruja | Pain in eyes |
| Netra Vrana | Corneal ulcer |
| Netrabhisyanda | Conjunctivitis |
| Netradrsti | Eye sight |
| Netragata | Related to eye |
| Netraroga | Eye disorder |
| Netraroga (Dhavanartha) | Eye disorder (for cleansing) |
| Netrasrava | Chronic dacrocystitis/epiphora |
| Netravikara | Disorder of eye |
| Nidaana | Causes, refers to the etiology or cause of the disease |
| Nidaana Parivarjana | Removal or avoiding of causative factors related to the disease |
| Nidra | Sleep |
| Nija | Innate, one’s own, internal |
| Nilika | Mole |
| Niruha (Basti) | An enema of herbal decoctions |
| Nyaccha | Naevus/mole |
| Ojaksaya | Loss of body strength (immunity) |
| Ojaksaya | Loss of body strength (immunity) |
| Ojas | Vigour, strength and vitality that is the essence of all tissues (Dhatus) |
| Paala Roga | (Likely same as Patala Roga or a typo) |
| Pachaniya | Substances that help in proper digestion |
| Padadaha | Burning sensation of feet |
| Padminikantaka | Papilloma of skin |
| Paittika Netra Roga | Eye disease due to Pitta dosa |
| Paksaghata | Paralysis/Hemiplegia |
| Paksavadha | Paralysis/Hemiplegia |
| Paksmakopa | Trichiasis/Entropion |
| Paktisula | Duodenal ulcer |
| Pakvatisara | Chronic diarrhoea |
| Palita | Graying of hair |
| Palitya | Graying of hair, particularly of scalp |
| Pama | Eczema |
| Panajirna | Alcoholic intoxication |
| Panatyaya | Acute alcoholism |
| Panavibhrama | Delirium due to alcohol intoxication |
| Panchakarma | According to Ayurveda this refers to the five cleansing therapies i.e. Vaman, Virechana, Basti, Nasya and Raktamokshana. |
| Pandu | Anaemia |
| Pandu Roga | Anaemia |
| Pangu | Paraplegia |
| Pangutva | Paraplegia |
| Pangutva | Paraplegia |
| Panguvata | Paraplegia |
| Parigarbhika Roga | Kwashiorkor |
| Parikarta | Fissure-in-ano |
| Parinama Sula | Duodenal ulcer |
| Parisarpa | Erysipelas |
| Parsva Siroruja | Hemicrania |
| Parsva Sopha | Swelling in lateral part of chest region |
| Parsva Sopha | Pleural effusion |
| Parsva Sula | Intercostal neuralgia and pleurodynia |
| Parsvaruja | Intercostal neuralgia and pleurodynia |
| Patala | Layers of Eye |
| Patala Arbuda | Growth in layers of eye |
| Patala Roga | Disorders of layers of eye |
| Patala Roga | Disorders of layers of eye |
| Phakka Roga | Ricketts |
| Phiranga | Syphilis |
| Phiranga Roga | Syphilis |
| Phirangaja Vrana | Chancroid |
| Phupphusa Roga | Lung disorders |
| Picchasrava | Mucoid discharge |
| Pidaka | Carbuncle |
| Pinasa | Chronic rhinitis/sinusitis |
| Pipasa | Thirst |
| Pitaka | Boil/Carbuncle |
| Pitta | It is one of the three Doshas i.e. the bile humour, entire hormones, enzymes, coenzymes and agencies responsible for the physiochemical processes of the body |
| Pitta Daha | Burning sensation due to Pitta dosa |
| Pitta Jvara | Fever due to Pitta dosa |
| Pitta Roga | Disease due to Pitta dosa |
| Pittaja Gulma | Tumour due to Pitta dosa |
| Pittaja Kustha | Skin disorders due to Pitta dosa |
| Pittaja Netra Roga | Eye disease due to Pitta dosa |
| Pittaja Pandu | Anaemia due to Pitta dosa |
| Pittaja Roga | Disease due to Pitta dosa |
| Pittaja Sirahsula | Headache due to Pitta dosa |
| Pittaja Siroruja | Headache due to Pitta dosa |
| Pittaja svasa | Asthma due to Pitta dosa |
| Pittaja Unmada | Insanity due to Pitta dosa |
| Pittarsa | Piles due to Pitta dosa |
| Pittatisara | Diarrhoea due to Pitta dosa |
| Pittavikara | Disorder of Pitta dosa |
| Pittavyadhi | Disease of Pitta dosa |
| Pliha | Splenic disease |
| Pliha Roga | Splenic disease |
| Pliha Vrddhi | Splenomegaly |
| Pliha-Yakrdroga | Disorder of Spleen and Liver |
| Plihamaya | Splenic disease |
| Pliharuja | Pain due to splenic disease |
| Plihayakrtvrddhi | Enlargement of liver and Spleen |
| Plihodara | Disorder of Spleen, Ascites associated with spleenomegaly |
| Pradara | Excessive vaginal discharge |
| Pralapa | Delirious speech |
| Pralepaka Jvara | Hectic fever |
| Prameha | Urinary disorders |
| Prameha and Mutramarga Roga | Polyurias and diseases of Urinary tract |
| Prameha Pidaka | Diabetic carbuncle |
| Praseka | Excessive salivation |
| Prasutavata | Puerperal disorder |
| Prasveda | Excessive sweating |
| Pratisyaya | Coryza |
| Pratituni/ pratuni | Renal/ureteric colic |
| Pratyasthila | Rectovesical tumour |
| Pratyasthila | Distended, Regid abdomen resembling paralytic Ileus |
| Pravahika | Dysentery |
| Prstha Ruja | Pain in the lower back |
| Prstha Stambha | Stiffness of the Lower back |
| Prstha sula | Backache |
| Purana Tvagroga | Degenerative/Chronic Skin disorder |
| Puranajvara | Chronic fever |
| Puyasrava | Secretion of pus |
| Rajahkrcchra | Scanty menstruation (Oligomenorrhea) |
| Rajayaksma | Tuberculosis |
| Rajo Sula | Dysmenorrhea |
| Rajodosa | Menstrual disorder |
| Rajorodha | Obstruction to menstrual flow |
| Rakta Dosa | Vitiation of Blood and its components |
| Rakta Nisthivana | Blood stained sputum |
| Raktagata Vata | Rakta affected by Vata Dosa |
| Raktakasa | Coughing up blood from the respiratory tract-hemoptysis |
| Raktaksaya | Blood loss |
| Raktapitta | Bleeding disorder |
| Raktapradara | Menorrhagia or Metrorrhagia or both |
| Raktapravahika | Bacillary dysentery |
| Raktaroga | Diseases of blood |
| Raktarsa | Bleeding haemorrhoids |
| Raktasrava | Haemorrhage |
| Raktatisara | Diarrhoea with Bleeding |
| Raktavami | Haematemesis |
| Raktavataja Sopha | Inflammation due to Rakta and Vata dosa |
| Raktavikara | Disorders of blood |
| Rasayanarthi | Person seeking Rasayana effect |
| Ratryandha | Night blindness (Nyctalopia) |
| Retovikara | Diseases of Semen |
| Ruja | Pain |
| Sahaja Arsoroga | Congenital haemorrhoids |
| Samadosa | Dosa afflicted by Ama |
| Samipa Drsti | Myopia |
| Samnyasa | Coma |
| Sandhi Pida | Joint pain |
| Sandhigata Vata | Osteoarthropathy |
| Sandhivata | Osteoarthritis |
| Sanjnānāsa | Loss of consciouness |
| Sannipata Jvara | High grade fever due to vitiation of all Dosa |
| Sannipata Roga | Disease due to vitiation of all Dosa |
| Santatajvara | Continuous Fever |
| Sanyasa | Coma |
| Saptadhatugata Jvara | Fever due to involvement of all seven dhatu |
| Sarira Varna Hani | Change in body complexion |
| Sarkara | Gravel in urine |
| Sarkarameha | Crystalluria |
| Sarpa Visa | Snake poison |
| Sarpadamsa | Snake bite |
| Sarpadamsta | Snake bite |
| Saruja Vrana | Painful ulcer |
| Sarva Balaroga | All types of Pediatric conditions |
| Sarva Dhatu Sosa | Emaciation of all tissues |
| Sarva Jvara | All types of fevers |
| Sarva Krcchra Sadhya Roga | All types of disorders with difficult prognosis |
| Sarva Roga | All diseases |
| Sarva sula | All kinds of pain |
| Sarvajvara | All types of fevers |
| Sarvanga Graha | Stiffness and Tightness in all limbs |
| Sarvanga Grahana | Stiffness and Tightness in all limbs |
| Sarvanga Kampa | Generalized tremors |
| Sarvanga Sotha | Anasarca |
| Sarvanga Vata | Quadriplegia |
| Sarvangasotha | Generalized tremors |
| Sarvatisara | All types of diarrhoea |
| Sastraprahara Vrana | Wounds due to sharp weapons |
| Satatadi Jvara | Continous fever and the like |
| Savrana Sukra | Corneal ulcer/Ulcerative keratitis |
| Sidhma | Pityriasis versicolor |
| Sighra Sukra Skhalana | Premature ejaculation |
| Sikata Vartma | Granular Eyelid disorder |
| Sirahkampa | Tremor/Shaking of head |
| Sirahsula | Headache |
| Sirodaha | Burning sensation in the head |
| Sirogata Vata | Neurological disorders of head |
| Sirograha | Stiffness in head |
| Sirogriva Stambha | Stiffness in head and neck |
| Siroroga | Disease of head |
| Siroruja | Headache |
| Sita Jvara | Fever with chills |
| Sitapitta | Urticaria |
| Skandha Suskata | Emaciation of shoulder |
| Slesmajvara | Fever due to Kapha dosa |
| Slesmapittaja Kasa | Cough due to Kapha and Pitta |
| Slesmavikara | Disorder due to Kapha dosa |
| Slesmodara | Ascites due to Kapha dosa |
| Slipada | Filariasis |
| Smasru Patana | Falling of hair of beard and moustache |
| Smrti | Memory |
| Smrti Bhramsa | Impairment of memory |
| Smrti Daurbalya | Weak memory |
| Smrti Ksaya | Loss of memory |
| Smrtibhrama | Impairment of memory |
| Smrtihani | Loss of memory |
| Smrtiksina | Decrease in memory |
| Smrtinasa | Loss of memory |
| Smrtiprada | Improves memory |
| Snayu Bhagna | Tendon tear |
| Snayudaurbalya | Rupture of ligaments |
| Snayuruja | Pain in tendons |
| Snayuvata | Inflammation of ligaments |
| Somaroga | Polyuria in females |
| Sopha | Oedema |
| Sosa | Cachexia |
| Sotha | Inflammation |
| Sotha Roga | Inflammatory disease |
| Sparsa sunyata | Lack of tactile sensation |
| Sphota | Boil |
| Sphotaka | Boil |
| Srama | Fatigue/lethargy |
| Sravana Lopa | Impaired hearing |
| Srikhalā Vata | Neurological disorder |
| Sroni sula | Pain in pelvic region |
| Srotorodha | Obstruction to srotas |
| Stanasula | Pain in breast tissue |
| Stanya Dusti | Vitiation of breast milk |
| Stanya Ksaya | Decrease in breast milk |
| Stanyadvesa | Aversion to breast milk |
| Sthaulya | Obesity |
| Sthavara Visa | Poison of plant and mineral origin |
| Sthulata | Obesity |
| Stimita | Feeling of Numbness |
| Striroga | Gynaecological disorders |
| Sukadosa | Ulcer on penis due to chemical agents |
| Sukla | Opacity |
| Sukra | Semen |
| Sukra (Opacity) | SNA |
| Sukra Dosa | Vitiation of semen |
| Sukra Ksaya | Deficiency of semen |
| Sukra Vikara | Disorders of semen |
| Sukraksaya | Oligospermia |
| Sukrameha | Spermatorrhoea |
| Sukraroga | Diseases of semen |
| Sula | Colicky Pain |
| Sularoga | Gastric ulcer/Duodenal ulcer/Colic |
| Supta Vata | Numbness |
| Suptajihvatva | Numbness of tongue |
| Suryavarta | Sinusitis |
| Suryavarta | Sinusitis |
| Suskarsa | Non bleeding Haemorrhoids |
| Sutika Jvara | Post partum Fever puerperal fever |
| Sutika Roga | Puerperal disease |
| Sutika Vata | Neurological disorders of puerperium |
| Sutikaroga | Post Partum disease |
| Sutikaroga | Post partum disorder puerperal disorders |
| Svapnadosa | Nocturnal emission |
| Svara Ksaya | Aphasia |
| Svarabheda | Hoarseness of voice |
| Svarahina | Aphasia |
| Svarakarsya | Aphasia |
| Svasa | Dyspnoea/Asthma |
| Svasanaka Jvara | Pneumonia |
| Svayathu | Oedema |
| Sveda | Sweat |
| Svedadhikya | Excessive Sweating |
| Sveta Pradara | Leucorrhoea |
| Svitra | Leucoderma/Vitiligo |
| Svitraroga | Leucoderma/Vitiligo |
| Tamaka Svasa | Bronchial asthma |
| Tandra | Lassitude |
| Taruna Jvara | Acute fever |
| Tilaka | Non-elevated mole |
| Timira | early stage of Catract |
| Timira Roga | Blindness |
| Tridosajatisara | Diarrhoea due to all Dosa |
| Trika Ruja | Pain in sacral region |
| Trika Sopha | Swelling in sacral region |
| Trika sula | Pain in sacral region |
| Trsna | Thirst |
| Trt | Thirst |
| Tuni | Neuralgic pain renal/ureteric colic |
| Tvagdosa | Skin disorder |
| Tvagrakta dosa | Skin and blood disorder |
| Tvagroga | Skin disease |
| Tvak rakta roga | Skin and blood disorder |
| Tvakrauksya | Dryness of skin |
| Udakodara | Ascites |
| Udara | Diseases of abdomen / enlargement of abdomen |
| Udararoga | Ascites |
| Udararuja | Pain in the abdomen |
| Udarasula | Pain in the abdomen |
| Udarda | Urticaria |
| Udavarta | Condition in which threre is upward movement of vayu |
| Ugra Atisara | Severe Diarrhoea |
| Unmada | Mania/Psychosis |
| Unmada Hrdroga | Insanity Heart disease |
| Upadamsa | Syphilis/Soft chancre |
| Upadamsaja Vrana | Chancroid |
| Upajihvika | Epiglottis |
| Urahksata | Chest wound / injured chest/ disease of lungs |
| Urastoya | Pleural effusion/ Hydrothorax |
| Urdhva Jatrugata Roga | Diseases of head and neck |
| Urdhvagata Raktapitta | Bleeding from orifices of the upper part of the body |
| Urdhvajatrugata Roga | Disorders of body parts above clavicle |
| Urdhvanga Ratkapitta | Bleeding from orifices of the upper part of the body |
| Urdhvanga Roga | Disordrs of Head and brain |
| Urdhvaraktapitta | Bleeding from orifices of the upper part of the body |
| Urdhvasvasa | Shallow breathing/ Shortness of breath |
| Urograha | Stiffness and Tightness in the chest |
| Uroroga | Disease of thorax |
| Uru Sopha | Swollen thighs |
| Urugraha | Stiffness in thighs |
| Urustambha | Stiffness in thigh muscles |
| Urusula | Pain in thighs |
| used as a Rasayana | Nutrient to body and mind with adapto-immuno-neuro-endocrino-modulator properties |
| Used as Balya | Improves physical strength |
| Used as Balya, Rasayana, Vajikarana | Improves strength,Nutrient to body and mind with adapto-immuno-neuro-endocrino-modulator properties and Aphrodisiac |
| Used as Rasayana | Nutrient to body and mind with adapto-immuno-neuro-endocrino-modulator properties |
| Used as Rasayana | Nutrient to body and mind with adapto-immuno-neuro-endocrino-modulator properties |
| used as Rasayana, Medhya, Smrtiprada | Nutrient to body and mind with adapto-immuno-neuro-endocrino-modulator properties, Brain Tonic / Nootropic, Improves memory |
| used as Rasayana, Vajikara, Medhya, Smrtivardhaka | Nutrient to body and mind with adapto-immuno-neuro-endocrino-modulator properties, Aphrodisiac, Brain Tonic / Nootropic, Improves memory |
| Used as Vajikara | Aphrodisiac |
| Used as Vajikara and Rasayana | Aphrodisiac and Nutrient to body and mind with adapto-immuno-neuro-endocrino-modulator properties |
| Used as Virecaka | Purgative |
| used as Vrsya Rasayana Medhya | Aphrodisiac and Nutrient to body and mind with adapto-immuno-neuro-endocrino-modulator properties, Brain Tonic / Nootropic |
| Utklesa | Nausea |
| Vaivarnya | Discolouration |
| Vajikara | Aphrodisiac |
| Vajikarana | Aphrodisiac |
| Vajikarana also | Aphrodisiasis |
| Vajikaranartha | For aphrodisiac property |
| Vakdosa | Disorder of speech |
| Vaksa Suskata | Emaciation of chest muscles |
| Vakswara Bhanga | Impairment of speech and voice |
| Vaktraruja | Pain in mouth |
| Vali | Wrinkles in the skin |
| Vali Palita | Wrinkles in skin and graying of hair |
| Valmika Arbuda | Malignant growth (Cancer) |
| Vami | Vomiting |
| Vandhyaroga | Infertility |
| Vandhyatva | Infertility |
| Vanksana Ruja | Pain in groin |
| Vanksana Sula | Pain in the groin |
| Varcovibandha | Impaction due to Hard stools |
| Vardhma | Hernia |
| Varna | Complexion |
| Varna Vikara | Pigmentation disorder |
| Varna Vikrti | Pigmentation disorder |
| Vartma Roga | Disease of eye lids |
| Vastiroga | Diseases of urinary bladder |
| Vastisula | Pain in urinary bladder |
| Vata Gulma | Lump due to Vata dosa |
| Vata Jvara | Fever due to Vata dosa |
| Vata Kapha Jvara | Fever due to Vata dosa and Kapha dosa |
| Vata Kapha Roga | Disease due to Vata dosa and Kapha dosa |
| Vata Kaphaja Gulma | Lump due to Vata dosa and Kapha dosa |
| Vata Kaphaja Roga | Disease due to Vata dosa and Kapha dosa |
| Vata Pittaja Roga | Disease due to Vatadosa and Pitta dosa |
| Vata Slesma Roga | Disease due to Vata and Kapha dosa |
| Vata Vaigunya | Abnormality of Vata dosa |
| Vata Vidradhi | Abscess due to Vata dosa |
| Vata Vyadhi | Disease due to Vata dosa |
| Vataja Grahani | Malabsorption due to Vata dosa |
| Vataja Kasa | Cough due to Vata dosa |
| Vataja Siroruja | Headache due to Vata dosa |
| Vataja sula | Pain due to Vata dosa |
| Vatajaroga | Disease due to Vata dosa |
| Vatakaphaja Grahani | Malabsorption due to Vata Kapha dosa |
| Vatakapharoga | Disease due to Vata Kapha dosa |
| Vatakundalika | Scanty and painful flow of urine |
| Vatapitta Jvara | Fever due to Vata dosa and Pitta dosa |
| Vatapitta Roga | Disease due to Vata dosa and Pitta dosa |
| Vatapittaja Vastiroga | Disorder of Urinary tract and Bladder due to Vata Pitta dosa |
| Vatapittakapha Vrana | Ulcer due to Vata Pitta Kapha dosa |
| Vatarakta | Gout |
| Vataraktaruja | Pain due to Vatarakta |
| Vataroga | Disease due to Vata dosa |
| Vataslesma Jvara | Fever due to Vata dosa and Slesma dosa |
| Vataslesma Pratisyaya | Rhinitis due to Vata dosa and Slesma dosa |
| Vataslesmaja Roga | Disorders of Vata Kapha dosa |
| Vataslesmaja Vrsanasotha | Inflammatory swelling/ ulceration of scrotum due to Vata Kapha dosa |
| Vataslesmajaksaya | Pthisis due to Vata dosa and Slesma dosa |
| Vataslesmatisara | Diarrhoea due to Vata and Kapha dosa |
| Vatasonita | Gout |
| Vatasula | Pain due to Vata dosa |
| Vatavikara | Disorder due to Vata dosa |
| Vatodara | Distention of abdomen due to gases |
| Vayusula | Colicky Pain due to Vata dosa |
| Vibandha | Constipation |
| Vicarcika | Eczema |
| Vidradhi | Abscess |
| Vidvibandha | Constipation |
| Vipadika | Scaly lesions on palms and soles |
| Vipluta Yoniroga | Disorder of Vagina(vaginismus) |
| Virecanartha | For purgation |
| Viryaksaya | Azoospermia |
| Visa | Poison |
| Visa Vikara | Disorders due to poison |
| Visamagni | Impaired digestion power |
| Visamajvara | Intermittent fever |
| Visamajvara | Intermittent fever |
| Visarpa | Erysipelas |
| Visarpa | Erysipelas |
| Visavikara | Morbidity due to Poisonous substance |
| Visphota | Blister |
| Visphotaka | Blisterous eruption |
| Vistambha | Constipation |
| Visuci | Gastro-enteritis with piercing pain |
| Visucika | Gastro-enteritis with piercing pain |
| Visvaci | Brachial neuralgia |
| Vitbandha | Constipation |
| Vitsanga | Constipation |
| Vrana | Ulcer |
| Vrana Dosa | Ulcer |
| Vranopacara | Ulcer debriding agent |
| Vrddhi | Inguino-scrotal swellings |
| Vrddhiroga | Inguino-scrotal swellings |
| Vrkkaroga | Disorders of Kidney |
| Vrkkasula | Kidney Pain |
| Vrsya sakti | Vigour/Aphrodisiac strength |
| Vyadhikarsita Nastasukra | Oligospermia due chronic illness |
| Vyanga | Pigmentation disorder |
| Yakrt roga | Disease of liver |
| Yakrtodara | Enlargement of liver (Hepatomegaly) |
| Yakrtpliharoga | Disorder of Liver and Spleen |
| Yakrtplihasula | Pain due to hepatic and splenic diseases |
| Yakrtplihavrddhi | Enlargement of liver and spleen |
| Yakrtplihodara | Disease of liver and spleen |
| Yakrtvikara | Disorder of liver |
| Yakrtvrddhi | Enlargement of liver (Hepatomegaly) |
| Yaksma | Tuberculosis |
| Yauvana Pitika | Acne vulgaris |
| Yoni Vikara | Disorder of Vagina |
| Yonibhramsa | Prolapse of Vaginal wall |
| Yonidosa | Disorder of female genital tract |
| Yoniroga | Disease of female genital tract |
| Yoniruja | Vaginal Pain |
| Yonisankocaka | Vaginal constricting agent |
| Yonisula | Pain in female genital tract |

*Supplementary Table 2. Mapping of Ayurvedic disease terms to DOIDs, MeSH IDs, and SYMP IDs. Mappings were manually curated by assessing semantic similarity between disease descriptions in the Ayurvedic Standard Treatment Guidelines, the Ayurvedic Pharmacopoeia, and relevant web resources.*

| **Ayurvedic Term** | **DOID** | **MESH ID** | **SYMP ID** | **Disease Node Label** |
| --- | --- | --- | --- | --- |
| Abhishyanda | DOID_6195; DOID_9699 | D003231; D009878 | SYMP_0000128 | Conjunctivitis; Ophthalmia Neonatorum |
| Abhishyanda; Kukurnaka | DOID_9699 | D009878 |  | Ophthalmia Neonatorum |
| Adhimantha | DOID_1686 | D005901 |  | Glaucoma |
| Adhmana |  | D005414 | SYMP_0000420; SYMP_0000509 | Bloating; Flatulence |
| Adhyavata | DOID_13189 | D006073 |  | Gout |
| Adhyavata; Vatarakta | DOID_13189 | D006073 |  | Gout |
| Agnimandya |  | D004415 |  | Dyspepsia |
| Ajarna | DOID_10041 | D004416 | SYMP_0000423 | Dysplastic Nevus Syndrome; Pyrosis |
| Ajirna | DOID_10041 | D004416 | SYMP_0000423 | Dysplastic Nevus Syndrome; Pyrosis |
| Ajirn∃A | DOID_10041 | D004416 | SYMP_0000423 | Dysplastic Nevus Syndrome; Pyrosis |
| Ajrirna | DOID_10041 | D004416 | SYMP_0000423 | Dysplastic Nevus Syndrome; Pyrosis |
| Ajīrṇa | DOID_10041 | D004416 | SYMP_0000423 | Dysplastic Nevus Syndrome; Pyrosis |
| Akshiroga | DOID_0070356 | D005128 |  | Eye Diseases |
| Aksiroga | DOID_0070356 | D005128 |  | Eye Diseases |
| Alasaka | DOID_8440 | D045823 |  | Ileus |
| All Types Of Jvara |  | D005334 | SYMP_0000613 | Fever |
| Amaja Sula |  | D003085 | SYMP_0000235 | Colic |
| Amajasula |  | D003085 | SYMP_0000235 | Colic |
| Amavata |  | D012216 |  | Rheumatic Diseases |
| Amla Pitta |  | D004418; D045262 | SYMP_0000425 | Dyspnea, Paroxysmal; Reticulocytosis |
| Amlapitta |  | D004418; D045262 | SYMP_0000425 | Dyspnea, Paroxysmal; Reticulocytosis |
| Anaha |  |  | SYMP_0000768 | Testicular Pain |
| Anaha; Antarvidradhi |  |  | SYMP_0000768 | Testicular Pain |
| And Cough |  | D003371 | SYMP_0000614 | Cough |
| Antarvidradhi | DOID_1283 | D006547 |  | Hernia |
| Antra Vrddhi |  | D000072281; D003085; D004415 | SYMP_0000235; SYMP_0000236; SYMP_0019142 | Colic; Colicky Pain; Dyspepsia; Lymphadenopathy |
| Antra Vrddhi; Mandagni |  | D004415 |  | Dyspepsia |
| Antra Vrddhi; Sula |  |  | SYMP_0000236 | Colicky Pain |
| Antra Vriddhi |  | D000072281; D003085; D004415 | SYMP_0000235; SYMP_0000236; SYMP_0019142 | Colic; Colicky Pain; Dyspepsia; Lymphadenopathy |
| Antravriddhi |  | D000072281; D003085; D004415 | SYMP_0000235; SYMP_0000236; SYMP_0019142 | Colic; Colicky Pain; Dyspepsia; Lymphadenopathy |
| Antravriddhi; Mandagni |  | D004415 |  | Dyspepsia |
| Antravriddhi; Shula |  |  | SYMP_0000236 | Colicky Pain |
| Apaca |  | D000072281 | SYMP_0019142 | Lymphadenopathy |
| Apasmara | DOID_1826 | D004827 |  | Epilepsy |
| Apasmāra | DOID_1826 | D004827 |  | Epilepsy |
| Appendicitis | DOID_8337 | D001064 |  | Appendicitis |
| Arbuda | DOID_14566; DOID_162 | D009369 |  | Disease Of Cellular Proliferation; Neoplasms |
| Ardita | DOID_13934 | D005158 | SYMP_0000283 | Facial Paralysis |
| Arma | DOID_0002116 | D011625 |  | Pterygium |
| Arocaka |  | D000855 | SYMP_0000523 | Anorexia |
| Arochaka |  | D000855 | SYMP_0000523 | Anorexia |
| Arsa | DOID_9745; DOID_9746 | D006484 |  | Hemorrhoids; Perianal Hematoma |
| Arsa; Arsha | DOID_9745; DOID_9746 | D006484 |  | Hemorrhoids; Perianal Hematoma |
| Arsa; Raktarśa | DOID_9746 | D006484 |  | Hemorrhoids |
| Arsha | DOID_9745; DOID_9746 | D006484 |  | Hemorrhoids; Perianal Hematoma |
| Artavavedana; Kastartava |  | D004412 |  | Dysmenorrhea |
| Arthritis | DOID_848 | D001168 | SYMP_0019169 | Arthritis |
| Aruchi |  | D000855 | SYMP_0000523 | Anorexia |
| Aruci |  | D000855 | SYMP_0000523 | Anorexia |
| Aruæsika | DOID_3087 | D005891 |  | Gingivitis |
| Arśa | DOID_9745; DOID_9746 | D006484 |  | Hemorrhoids; Perianal Hematoma |
| Ascites Associated With Spleenomegaly) |  | D001201 | SYMP_0000526 | Ascites |
| Ashmari |  | D002137 |  | Calculi |
| Ashmari; Asmara |  | D002137 |  | Calculi |
| Asmara | DOID_585 | D002137; D053040 |  | Calculi; Nephrolithiasis |
| Asmari | DOID_585 | D002137; D053040 |  | Calculi; Nephrolithiasis |
| Asrgdara |  | D004412; D008796 | SYMP_0000855 | Dysmenorrhea; Metrorrhagia |
| Asrgdhara |  | D004412; D008796 | SYMP_0000855 | Dysmenorrhea; Metrorrhagia |
| Asrigdara |  | D008796 | SYMP_0000855 | Metrorrhagia |
| Asrigdara Ruja |  | D004412; D008796 | SYMP_0000855 | Dysmenorrhea; Metrorrhagia |
| Asthala | DOID_11132; DOID_2883 | D011470 |  | Prostatic Hyperplasia; Prostatic Hypertrophy |
| Asthi Bhagna |  | D050723 |  | Fractures, Bone |
| Asthi Chyuti |  | D004204 |  | Joint Dislocations |
| Asthi Cyuti |  | D004204 |  | Joint Dislocations |
| Asthi Ruja |  |  | SYMP_0020023 | Bone Pain |
| Asthibhagna |  | D050723 |  | Fractures, Bone |
| Asthicyuti |  | D004204 |  | Joint Dislocations |
| Asthila | DOID_11132; DOID_2883 | D011470 |  | Prostatic Hyperplasia; Prostatic Hypertrophy |
| Asthiruja |  |  | SYMP_0020023 | Bone Pain |
| Asthivata | DOID_0080001 | D001847 |  | Bone Diseases |
| Atisara | DOID_13250 | D003967; D004403 | SYMP_0000570 | Diarrhea; Dysentery |
| Atisāra | DOID_13250 | D003967; D004403 | SYMP_0000570 | Diarrhea; Dysentery |
| Avabahuka | DOID_14188; DOID_14276 | D002062; D019534 |  | Bursitis; Shoulder Impingement Syndrome |
| Badhiratva |  | D003638 | SYMP_0000019 | Deafness |
| Badhirya |  | D003638 | SYMP_0000019 | Deafness |
| Balaksaya |  | D000073496 |  | Frailty |
| Bastiroga | DOID_18 | D014570 |  | Urinary System Disease; Urologic Diseases |
| Bhagandara | DOID_0060328 | D012003 |  | Rectal Fistula |
| Bhagandara; Nad Avrana | DOID_0060328 | D012003 |  | Rectal Fistula |
| Bhagandara; Nadavrana | DOID_0060328 | D012003 |  | Rectal Fistula |
| Bhagandara; Nadivrana | DOID_0060328 | D012003 |  | Rectal Fistula |
| Bhootonmada | DOID_1742; DOID_2468 | D011605 |  | Psychoses, Substance-Induced; Psychotic Disorder |
| Bhrama | DOID_2479; DOID_9849 | D008575; D014717 | SYMP_0000399 | Meniere Disease; Vertigo |
| Bhutonmada | DOID_1742; DOID_2468 | D011605 |  | Psychoses, Substance-Induced; Psychotic Disorder |
| Body Pains |  |  | SYMP_0000230 | Body Ache |
| Buddhimandata | DOID_1059 | D008607 |  | Intellectual Disability |
| Chardi | DOID_1059 | D008607; D014839; D020250 | SYMP_0019145 | Intellectual Disability; Postoperative Nausea And Vomiting; Vomiting |
| Dadru | DOID_0050596 | D013622 |  | Taeniasis |
| Daha |  | D002056 |  | Burns |
| Danta Roga | DOID_1091 | D014076 |  | Tooth Diseases |
| Dantaroga | DOID_1091 | D014076 |  | Tooth Diseases |
| Dantasula | DOID_1091 | D014076; D014098 | SYMP_0000438 | Tooth Diseases; Toothache |
| Dantaveshtaka |  | D006209; D006472 |  | Halitosis; Oral Hemorrhage |
| Daurbalya |  | D000073496 |  | Frailty |
| Dengue | DOID_12205 | D003715 |  | Dengue |
| Dhatu Kshaya |  | D002100 | SYMP_0000174; SYMP_0000402 | Cachexia; Wasting |
| Dhatuksaya |  | D002100 | SYMP_0000174; SYMP_0000402 | Cachexia; Wasting |
| Duradristi | DOID_9834 | D006956 |  | Hyperopia |
| Dustavrana |  | D014456 |  | Ulcer |
| Ekakushta | DOID_4398 | D011565 |  | Psoriasis |
| Ekangasosa | DOID_767 | D009133 |  | Muscular Atrophy |
| Galagraha |  |  | SYMP_0000505 | Throat Pain |
| Galaroga And Daurbalya |  | D000073496 | SYMP_0000385 | Frailty; Throat Symptom |
| Ganda | DOID_4637; DOID_4889 | D014388 |  | Cervical Adenitis; Tuberculosis, Lymph Node |
| Gandamala | DOID_4637; DOID_4889 | D014388 |  | Cervical Adenitis; Tuberculosis, Lymph Node |
| Garbhadosha |  | D000013 |  | Congenital Abnormalities |
| Graha Dosha | DOID_2468 |  |  | Psychotic Disorder |
| Grahadosa | DOID_2468 |  |  | Psychotic Disorder |
| Grahana |  | D008286 |  | Malabsorption Syndromes |
| Grahani |  | D008286 |  | Malabsorption Syndromes |
| Granthi |  | D003560 |  | Cysts |
| Gridhrasa |  | D012585 |  | Sciatica |
| Gridhrasa; Gridhrasi |  | D012585 |  | Sciatica |
| Gridhrasi |  | D012585 |  | Sciatica |
| Guhyavrana |  | D014456 |  | Ulcer |
| Gulma |  |  | SYMP_0000799 | Abdominal Lump |
| Halamaka | DOID_13603 | D007565; D041781 | SYMP_0000539 | Jaundice; Jaundice, Obstructive |
| Halamaka; Kamala |  | D007565 | SYMP_0000539 | Jaundice |
| Halimaka | DOID_13603 | D007565; D041781 | SYMP_0000539 | Jaundice; Jaundice, Obstructive |
| Halimaka; Kamala |  | D007565 | SYMP_0000539 | Jaundice |
| Halīmaka | DOID_13603 | D007565; D041781 | SYMP_0000539 | Jaundice; Jaundice, Obstructive |
| Hanugraha |  | D014313 |  | Trismus |
| Hanustambha |  | D014313 |  | Trismus |
| Hikka |  | D006606 |  | Hiccup |
| Hillasa |  | D009325 | SYMP_0000458 | Nausea |
| Hridgraha | DOID_6000 | D006333 | SYMP_0000292 | Heart Failure |
| Hridroga | DOID_114 | D006331 |  | Heart Diseases |
| Hridruja |  | D000787 |  | Angina Pectoris |
| Hrillasa |  | D009325 | SYMP_0000458 | Nausea |
| Hritgraha | DOID_6000 | D006333 | SYMP_0000292 | Heart Failure |
| Hritsula |  | D000787 |  | Angina Pectoris |
| Hrllasa |  | D009325 | SYMP_0000458 | Nausea |
| Hrtsoola |  | D000787 |  | Angina Pectoris |
| Hr#Droga | DOID_114 | D006331 |  | Heart Diseases |
| Hṛdroga | DOID_114 | D006331 |  | Heart Diseases |
| Indralupta | DOID_986 | D000506 |  | Alopecia Areata |
| Indralupta; Kesapata; Khalitya | DOID_987 | D000505 |  | Alopecia |
| Indralupta; Khalitya | DOID_987 | D000505 |  | Alopecia |
| Influenza | DOID_8469 | D007251 |  | Influenza, Human |
| Jalodara |  | D001201 | SYMP_0000526 | Ascites |
| Janu Sula | DOID_14284 | D046788 | SYMP_0000442 | Knee Pain; Patellofemoral Pain Syndrome |
| Janustabdhata | DOID_14284 | D046788 | SYMP_0000442 | Knee Pain; Patellofemoral Pain Syndrome |
| Janusula | DOID_14284 | D046788 | SYMP_0000442 | Knee Pain; Patellofemoral Pain Syndrome |
| Jihvastambha | DOID_10944 | D014060 |  | Tongue Diseases |
| Jvara |  | D005334 | SYMP_0000613 | Fever |
| Jwara |  | D005334 | SYMP_0000613 | Fever |
| Kaca; Timira | DOID_83 | D002386 |  | Cataract |
| Kacha; Timira | DOID_83 | D002386 |  | Cataract |
| Kamala |  | D007565 | SYMP_0000539 | Jaundice |
| Kampa |  | D014202 | SYMP_0000162 | Tremor |
| Kandoo |  | D011537 | SYMP_0000432 | Itching; Pruritus |
| Kandu |  | D011537 | SYMP_0000432 | Itching; Pruritus |
| Kaphadhika Tamaka Swasa | DOID_2841 | D001249 |  | Asthma |
| Kaphadhika Tamaka Swasa; Shvasa | DOID_2841 | D001249 |  | Asthma |
| Kaphaja Agnimandya |  | D004415 |  | Dyspepsia |
| Karna Roga | DOID_2742 | D004427 |  | Ear Diseases |
| Karnanada |  | D014012 | SYMP_0000393 | Tinnitus |
| Karnaroga | DOID_2742 | D004427 |  | Ear Diseases |
| Karnashula |  | D004433 | SYMP_0000437 | Earache |
| Karnasula |  | D004433 | SYMP_0000437 | Earache |
| Karshya |  | D004614 | SYMP_0000360 | Emaciation |
| Karsya |  | D004614 | SYMP_0000360 | Emaciation |
| Kasa |  | D003371 | SYMP_0000614 | Cough |
| Kashtaartava |  | D004412 |  | Dysmenorrhea |
| Kashtaartava With Heavy Bleeding |  | D004412 |  | Dysmenorrhea |
| Kashtaartava With Scanty Bleeding |  | D004412 |  | Dysmenorrhea |
| Kastartava |  | D004412 |  | Dysmenorrhea |
| Kati Graha | DOID_2300 | D013169 |  | Spondylolysis |
| Katigraha | DOID_2300 | D013169 |  | Spondylolysis |
| Katistambha |  | D017116 |  | Low Back Pain |
| Kaubja | DOID_4667 | D007738 |  | Kyphosis |
| Kesa Patana |  |  | SYMP_0000440 | Hair Loss |
| Kesapata And Palita | DOID_987 | D000505 |  | Alopecia |
| Khalitya | DOID_987 | D000505 |  | Alopecia |
| Khanja |  |  | SYMP_0000479 | Abnormality Of Gait |
| Khanjavata |  |  | SYMP_0000479 | Abnormality Of Gait |
| Kotha; Shatapitta; Udarda | DOID_1555 | D014581 | SYMP_0000434 | Urticaria |
| Kotha; Shitapitta; Udarda | DOID_1555 | D014581 | SYMP_0000434 | Urticaria |
| Krimi | DOID_883 | D006373 |  | Helminthiasis |
| Krishatva |  | D002100 | SYMP_0000402 | Cachexia |
| Krostusarsaka | DOID_848 | D001168 | SYMP_0019169 | Arthritis |
| Kr#Mi | DOID_883 | D006373 |  | Helminthiasis |
| Kr∃Mi | DOID_883 | D006373 |  | Helminthiasis |
| Ksata Ksaya |  | D004614 | SYMP_0000360 | Emaciation |
| Ksataksana |  | D012769; D013898 | SYMP_0000450 | Shock; Thoracic Injuries |
| Ksataksaya |  | D004614 | SYMP_0000360 | Emaciation |
| Ksaya | DOID_2957 | D014397 |  | Tuberculosis, Pulmonary |
| Kshaya | DOID_2957 | D014397 |  | Tuberculosis, Pulmonary |
| Ksina Retas | DOID_14228 | D009845 |  | Oligospermia |
| Ksina Sukra | DOID_14228 | D009845 |  | Oligospermia |
| Ksudrakustha; Mahakustha | DOID_37 | D012871; D012873 |  | Skin Diseases; Skin Diseases, Genetic |
| Kubja | DOID_4667 | D007738 |  | Kyphosis |
| Kubja Roga | DOID_4667 | D007738 |  | Kyphosis |
| Kubja Vata | DOID_4667 | D007738 |  | Kyphosis |
| Kubjata |  | D004392 |  | Dwarfism |
| Kukshisula |  | D017699 |  | Pelvic Pain |
| Kukshisula; Yoni Shula |  | D017699 |  | Pelvic Pain |
| Kumbhakamala |  | D006505 |  | Hepatitis |
| Kushta | DOID_37 | D012871; D012873 |  | Skin Diseases; Skin Diseases, Genetic |
| Kushtha | DOID_37 | D012871; D012873 |  | Skin Diseases; Skin Diseases, Genetic |
| Kustha | DOID_37 | D012871; D012873 |  | Skin Diseases; Skin Diseases, Genetic |
| Kustha; Tvagdosha | DOID_37 | D012873 |  | Skin Diseases, Genetic |
| Kustha; Tvagroga | DOID_37 | D012873 |  | Skin Diseases, Genetic |
| Kṛmi | DOID_883 | D006373 |  | Helminthiasis |
| Mada | DOID_252 | D000435; D011604 |  | Alcoholic Intoxication; Psychoses, Alcoholic |
| Mada; Madatyaya | DOID_252 | D000435; D011604 |  | Alcoholic Intoxication; Psychoses, Alcoholic |
| Madatyaya | DOID_252 | D000435; D011604 |  | Alcoholic Intoxication; Psychoses, Alcoholic |
| Madhumeha | DOID_9351 | D003920 |  | Diabetes Mellitus |
| Mahakustha | DOID_37 | D012871; D012873 |  | Skin Diseases; Skin Diseases, Genetic |
| Majjavata | DOID_4961 | D001855 |  | Bone Marrow Diseases |
| Malabandha |  | D003248 |  | Constipation |
| Malaria | DOID_12365 | D008288 |  | Malaria |
| Mandadristi |  | D015354 |  | Vision, Low |
| Mandagni |  | D004415 |  | Dyspepsia |
| Manya Stambha |  | D014103 | SYMP_0000617 | Torticollis |
| Manyaroga |  |  | SYMP_0000384 | Neck Symptom |
| Manyastambha |  | D014103 | SYMP_0000617 | Torticollis |
| Manyastambha Hanustambha |  | D014103; D014313 | SYMP_0000617 | Torticollis; Trismus |
| Medoroga | DOID_9970 | D009765 |  | Obesity |
| Meha |  | D011141 | SYMP_0000565 | Polyuria |
| Mookata | DOID_0060046 | D001037 | SYMP_0000508 | Aphasia |
| Moorcha |  | D013575 | SYMP_0000407; SYMP_0000608 | Syncope; Syncope And Collapse |
| Mootra Krcchra |  | D053159 | SYMP_0000485 | Dysuria |
| Mootrabandha |  | D016055 | SYMP_0000557 | Urinary Retention |
| Mootraroga | DOID_18 | D014570 |  | Urinary System Disease; Urologic Diseases |
| Mootr#Akrcchra |  | D053159 | SYMP_0000485 | Dysuria |
| Mudhagarbha |  | D004420 |  | Dystocia |
| Mukata | DOID_0060046 | D001037 | SYMP_0000508 | Aphasia |
| Mukha Daurgandhya |  | D006209 |  | Halitosis |
| Mukha Durgandhya |  | D006209 |  | Halitosis |
| Mukha Paka | DOID_9637 | D013280 |  | Stomatitis |
| Mukha Roga | DOID_403 | D009059 |  | Mouth Diseases |
| Mukhadaurgandhya |  | D006209 |  | Halitosis |
| Mukhadurgandha |  | D006209 |  | Halitosis |
| Mukhapaka | DOID_9637 | D013280 |  | Stomatitis |
| Mukharoga | DOID_403 | D009059 |  | Mouth Diseases |
| Murccha |  | D013575 | SYMP_0000407; SYMP_0019179 | Lightheadedness; Syncope |
| Murcha |  | D013575 | SYMP_0000407; SYMP_0019179 | Lightheadedness; Syncope |
| Murchha |  | D013575 | SYMP_0000407; SYMP_0019179 | Lightheadedness; Syncope |
| Mutra Roga | DOID_18 | D014570 |  | Urinary System Disease; Urologic Diseases |
| Mutraghata | DOID_5200 |  |  | Urinary Tract Obstruction |
| Mutrakrcchra |  | D053159 | SYMP_0000485 | Dysuria |
| Mutrakricchra |  | D053159 | SYMP_0000485 | Dysuria |
| Mutrakrichchhra |  | D053159 | SYMP_0000485 | Dysuria |
| Mutrakrichhra |  | D053159 | SYMP_0000485 | Dysuria |
| Mutrashtila | DOID_11132; DOID_2883 | D011470 |  | Prostatic Hyperplasia; Prostatic Hypertrophy |
| Mutravibandha |  | D016055 | SYMP_0000557 | Urinary Retention |
| Nad Avrana | DOID_0080171 |  |  | Esophageal Atresia/Tracheoesophageal Fistula |
| Nadavrana | DOID_0060328; DOID_0080171 | D012003 |  | Esophageal Atresia/Tracheoesophageal Fistula; Rectal Fistula |
| Nadivrana | DOID_0060328; DOID_0080171 | D012003 |  | Esophageal Atresia/Tracheoesophageal Fistula; Rectal Fistula |
| Naktadhya | DOID_8499 | D009755 |  | Night Blindness |
| Naktandhya | DOID_8499 | D009755 |  | Night Blindness |
| Nasa Roga | DOID_2825 | D009668 |  | Nose Diseases |
| Nayana Roga | DOID_0070356 | D005128 |  | Eye Diseases |
| Netra Srava | DOID_13757; DOID_9937 | D007766 | SYMP_0019164 | Chronic Dacryocystitis; Lacrimal Apparatus Diseases |
| Netrabhisyanda | DOID_6195 | D003231 | SYMP_0000128 | Conjunctivitis |
| Netrakandu |  |  | SYMP_0019163 | Eye Symptom |
| Netraroga | DOID_0070356 | D005128 |  | Eye Diseases |
| Netrasrava | DOID_13757; DOID_9937 | D007766 | SYMP_0019164 | Chronic Dacryocystitis; Lacrimal Apparatus Diseases |
| Oedema |  | D004487 | SYMP_0000538 | Edema |
| Osteomyelitis Of Femur; Osteomyelitis Of Pelvic Bones | DOID_1019 | D010019 |  | Osteomyelitis |
| Paksaghata | DOID_10969 | D006429; D010243 | SYMP_0000030 | Hemiplegia; Paralysis |
| Pakshaghat | DOID_10969 | D006429; D010243 | SYMP_0000030 | Hemiplegia; Paralysis |
| Pakshaghat; Pakshavadha | DOID_10969 | D006429; D010243 | SYMP_0000030 | Hemiplegia; Paralysis |
| Pakshaghata | DOID_10969 | D006429; D010243 | SYMP_0000030 | Hemiplegia; Paralysis |
| Pakshavadha |  | D020521 |  | Stroke |
| Paksmakopa | DOID_12397 | D004774; D058457 |  | Entropion; Trichiasis |
| Paktishula | DOID_1724 | D004381 |  | Duodenal Ulcer |
| Paktisula | DOID_1724 | D004381 |  | Duodenal Ulcer |
| Pama |  | D004485 | SYMP_0000289 | Eczema |
| Pama; Vicarcika |  | D004485 | SYMP_0000289 | Eczema |
| Pama; Vicharchika |  | D004485 | SYMP_0000289 | Eczema |
| Panasa | DOID_0050127; DOID_4483 | D012220; D012852 | SYMP_0000134 | Rhinitis; Sinusitis |
| Pandu | DOID_2355 | D000740 | SYMP_0000208 | Anemia |
| Pandu Roga | DOID_2355 | D000740 | SYMP_0000208 | Anemia |
| Pangu | DOID_607 | D010264 | SYMP_0000349 | Paraplegia |
| Pangutva | DOID_607 | D010264 | SYMP_0000349 | Paraplegia |
| Pan#D#U | DOID_2355 | D000740 | SYMP_0000208 | Anemia |
| Parikarta |  | D005401 |  | Fissure In Ano |
| Parikartika |  | D005401 |  | Fissure In Ano |
| Parinama Shula | DOID_1724 | D004381 |  | Duodenal Ulcer |
| Parinama Sula | DOID_1724 | D004381 |  | Duodenal Ulcer |
| Parisarpa | DOID_11330 | D004886 |  | Erysipelas |
| Parshva Shula | DOID_10882 | D009437; D011000 |  | Neuralgia; Pleurodynia, Epidemic |
| Parshva Sula | DOID_10882 | D009437; D011000 |  | Neuralgia; Pleurodynia, Epidemic |
| Parsva Sula | DOID_10882 | D009437; D011000 |  | Neuralgia; Pleurodynia, Epidemic |
| Parsvasoola | DOID_10882 | D009437; D011000 |  | Neuralgia; Pleurodynia, Epidemic |
| Parsvasula | DOID_10882 | D009437; D011000 |  | Neuralgia; Pleurodynia, Epidemic |
| Phuphphusa Roga | DOID_850 | D008171 |  | Lung Diseases |
| Pidaka | DOID_2176 | D002270 |  | Carbuncle |
| Pidika | DOID_2176 | D002270 |  | Carbuncle |
| Pinasa | DOID_0050127; DOID_4483 | D012220; D012852 | SYMP_0000134 | Rhinitis; Sinusitis |
| Pinasa; Pratishyay | DOID_4483 | D012220 |  | Rhinitis |
| Pinasa; Pratishyaya | DOID_4483 | D012220 |  | Rhinitis |
| Pinasa; Pratisyaya | DOID_4483 | D012220 |  | Rhinitis |
| Pipasa |  |  | SYMP_0000156 | Thirst |
| Pittaja Gulma |  |  | SYMP_0000799 | Abdominal Lump |
| Pittaja Kasa |  | D003371 | SYMP_0000614 | Cough |
| Pittarsa | DOID_9746 | D006484 |  | Hemorrhoids |
| Pl Aha | DOID_2529 | D013158 |  | Splenic Diseases |
| Plaha | DOID_2529 | D013158 |  | Splenic Diseases |
| Plaha Roga | DOID_2529 | D013158 |  | Splenic Diseases |
| Plaharoga | DOID_2529 | D013158 |  | Splenic Diseases |
| Plahodara |  | D013163 | SYMP_0000531 | Splenomegaly |
| Pleeha Roga | DOID_2529 | D013158 |  | Splenic Diseases |
| Pleeha Vriddhi | DOID_6376 | D006971; D013163 | SYMP_0000531 | Hypersplenism; Splenomegaly |
| Pliha | DOID_2529 | D013158 |  | Splenic Diseases |
| Pliha Roga | DOID_2529 | D013158 |  | Splenic Diseases |
| Pliha Vruddhi | DOID_6376 | D006971; D013163 | SYMP_0000531 | Hypersplenism; Splenomegaly |
| Plihamaya | DOID_2529 | D013158 |  | Splenic Diseases |
| Pliharuja |  |  | SYMP_0000547 | Right Upper Quadrant Abdominal Pain |
| Plihodara |  | D013163 | SYMP_0000531 | Splenomegaly |
| Pradara | DOID_3767 | D019522 | SYMP_0000398 | Vaginal Discharge |
| Prameha |  |  | SYMP_0000486 | Urinary System Symptom |
| Pramehapidaka | DOID_2176 | D002270 |  | Carbuncle |
| Praseka |  |  | SYMP_0000277 | Excess Salivation |
| Pratishyay | DOID_4483 | D012220 |  | Rhinitis |
| Pratishyaya | DOID_4483 | D012220 |  | Rhinitis |
| Pratisyaya | DOID_4483 | D012220 |  | Rhinitis |
| Pristha Stambha |  |  | SYMP_0000141 | Spasticity |
| Pristha Sula; Trika Sula |  |  | SYMP_0000006 | Backache |
| Pristhastambha |  |  | SYMP_0000141 | Spasticity |
| Pristhasula |  |  | SYMP_0000006 | Backache |
| Prstha Shula; Trika Shula |  |  | SYMP_0000006 | Backache |
| Prstha Sula |  |  | SYMP_0000006 | Backache |
| Puranajvara |  |  | SYMP_0000880 | Prolonged Fever |
| Rajayakshma | DOID_399 | D014375 | SYMP_0000174 | Tuberculoma; Wasting |
| Rajayaksma | DOID_399 | D014375 | SYMP_0000174 | Tuberculoma; Wasting |
| Rajo Dosha |  | D008599 |  | Menstruation Disturbances |
| Rajodosha |  | D008599 |  | Menstruation Disturbances |
| Rajorodha |  | D008599 |  | Menstruation Disturbances |
| Rakatadosa | DOID_74 | D006402 |  | Hematologic Diseases |
| Raktaatisara |  |  | SYMP_0020011 | Bloody Diarrhea |
| Raktanisthavana |  |  | SYMP_0000447 | Bloody Sputum |
| Raktapitta | DOID_1247 | D001778 | SYMP_0000007 | Bleeding; Blood Coagulation Disorders |
| Raktaroga | DOID_74 | D006402 |  | Hematologic Diseases |
| Raktarsa | DOID_9746 | D006484 |  | Hemorrhoids |
| Raktarsha | DOID_9746 | D006484 |  | Hemorrhoids |
| Raktasha | DOID_9746 | D006484 |  | Hemorrhoids |
| Raktasha; Shushkarsha | DOID_9746 | D006484 |  | Hemorrhoids |
| Raktasrshta | DOID_9746 | D006484 |  | Hemorrhoids |
| Raktatisara |  |  | SYMP_0020011 | Bloody Diarrhea |
| Raktatīsāra |  |  | SYMP_0020011 | Bloody Diarrhea |
| Raktavataja Sopha | DOID_8437 | D007415 |  | Intestinal Obstruction |
| Raktavikara | DOID_74 | D006402 |  | Hematologic Diseases |
| Regional Ileitis | DOID_0060189 | D007079 |  | Ileitis |
| Ruja |  | D010146 | SYMP_0000099 | Pain |
| Samapadristi | DOID_11830 | D009216 |  | Myopia |
| Sandhi Pida |  |  | SYMP_0000064 | Joint Pain |
| Sandhigata Vata | DOID_14283 | D010004 |  | Osteoarthropathy, Primary Hypertrophic |
| Sandhivata | DOID_8398 | D010003 |  | Osteoarthritis |
| Sanjnanasa |  |  | SYMP_0000718 | Loss Of Consciousness |
| Sarkara | DOID_0080653 | D014545; D052878 |  | Urinary Calculi; Urolithiasis |
| Sarpadansha |  | D012909 |  | Snake Bites |
| Sarpadaæsa |  | D012909 |  | Snake Bites |
| Sarvanga Kampa |  | D014202 | SYMP_0000162 | Tremor |
| Sarvanga Shotha |  |  | SYMP_0000433 | Anasarca |
| Sarvangasotha |  |  | SYMP_0000433 | Anasarca |
| Sarvatisara |  | D003967 |  | Diarrhea |
| Savrana Shukra | DOID_8463 | D003320 |  | Corneal Ulcer |
| Sharkara | DOID_0080653 | D014545; D052878 |  | Urinary Calculi; Urolithiasis |
| Shighra Shukra Skhalana | DOID_13709 | D061686 |  | Premature Ejaculation |
| Shirashula |  | D006261 | SYMP_0000504 | Headache |
| Shiroruja |  | D006261 | SYMP_0000504 | Headache |
| Shlapada | DOID_1080 | D005368 |  | Filariasis |
| Shlipada | DOID_1080 | D005368 |  | Filariasis |
| Shmasrupatana |  |  | SYMP_0000440; SYMP_0020044 | Hair Loss; Hair Shedding |
| Shoola |  |  | SYMP_0000236 | Colicky Pain |
| Shopha |  | D004487 | SYMP_0000538 | Edema |
| Shosa |  | D002100 | SYMP_0000402 | Cachexia |
| Shosha |  | D002100 | SYMP_0000402 | Cachexia |
| Shotha |  | D007249 | SYMP_0000061 | Inflammation |
| Shotha Roga |  | D007249 | SYMP_0000061 | Inflammation |
| Shrama |  | D005221 | SYMP_0019177 | Fatigue |
| Shukra Dosa | DOID_14228 | D009845 |  | Oligospermia |
| Shukra Dosha | DOID_14228 | D009845 |  | Oligospermia |
| Shukra Vikara | DOID_12336 | D007248 |  | Infertility, Male |
| Shukradosa | DOID_14228 | D009845 |  | Oligospermia |
| Shukraksaya | DOID_14228 | D009845 |  | Oligospermia |
| Shukrakshaya | DOID_14228 | D009845 |  | Oligospermia |
| Shula |  |  | SYMP_0000236 | Colicky Pain |
| Shula Roga | DOID_10808; DOID_1724 | D003085; D004381; D013276 | SYMP_0000235 | Colic; Duodenal Ulcer; Stomach Ulcer |
| Shularoga | DOID_10808; DOID_1724 | D003085; D004381; D013276 | SYMP_0000235 | Colic; Duodenal Ulcer; Stomach Ulcer |
| Shuskarsha | DOID_9746 | D006484 |  | Hemorrhoids |
| Shvaasa | DOID_2841 | D001249; D004417 | SYMP_0019153 | Asthma; Dyspnea |
| Shvasa | DOID_2841 | D001249; D004417 | SYMP_0019153 | Asthma; Dyspnea |
| Shvayathu |  | D004487 | SYMP_0000538 | Edema |
| Shvetapradara | DOID_3766 | D007973 |  | Leukorrhea |
| Shvitra | DOID_12306 | D014820 |  | Vitiligo |
| Shvitraroga | DOID_12306 | D014820 |  | Vitiligo |
| Shwasa | DOID_2841 | D001249; D004417 | SYMP_0019153 | Asthma; Dyspnea |
| Shweta Pradara | DOID_3766 | D007973 |  | Leukorrhea |
| Sirahsula |  | D006261 | SYMP_0000504 | Headache |
| Slipada | DOID_1080 | D005368 |  | Filariasis |
| Smriti Daurbalya |  |  | SYMP_0000719 | Memory Impairment |
| Smriti Ksaya |  |  | SYMP_0000719 | Memory Impairment |
| Smritibhrama |  |  | SYMP_0000543 | Memory Loss |
| Smritikshina |  |  | SYMP_0000719 | Memory Impairment |
| Smrti Daurbalya And Daurbalya |  | D000073496 | SYMP_0000719 | Frailty; Memory Impairment |
| Smrti Ksaya |  |  | SYMP_0000719 | Memory Impairment |
| Smrti Kshaya And Buddhi Mandya | DOID_1059 | D008607 | SYMP_0000719 | Intellectual Disability; Memory Impairment |
| Smrtinasa |  |  | SYMP_0000543 | Memory Loss |
| Snayubhagna |  | D000070598 |  | Anterior Cruciate Ligament Injuries |
| Snayuruja | DOID_971 | D052256 |  | Tendinopathy |
| Snayuvata |  | D007249 | SYMP_0000061 | Inflammation |
| Somaroga |  | D011141 | SYMP_0000565 | Polyuria |
| Sopha |  | D004487 | SYMP_0000538 | Edema |
| Sosa |  | D002100 | SYMP_0000402 | Cachexia |
| Sotha |  | D007249 | SYMP_0000061 | Inflammation |
| Sparsha Sunyata |  |  | SYMP_0000834 | Hypoesthesia |
| Sphota |  |  | SYMP_0019160 | Boil |
| Sphotaka |  |  | SYMP_0019160 | Boil |
| Srama |  | D005221 | SYMP_0019177 | Fatigue |
| Sravana Lopa |  | D003638 | SYMP_0000019 | Deafness |
| Sroni Sula |  | D017699 |  | Pelvic Pain |
| Sthaulya | DOID_9970 | D009765 |  | Obesity |
| Stimita |  |  | SYMP_0000834 | Hypoesthesia |
| Striroga | DOID_229 | D005831 |  | Genital Diseases, Female |
| Sukra Dosa | DOID_14228 | D009845 |  | Oligospermia |
| Sukraksaya | DOID_14228 | D009845 |  | Oligospermia |
| Sula |  |  | SYMP_0000236 | Colicky Pain |
| Suryavarta | DOID_0050127 | D012852 | SYMP_0000134 | Sinusitis |
| Sutika Roga |  | D011644 |  | Puerperal Disorders |
| Sutikaroga |  | D011644 |  | Puerperal Disorders |
| Svarabheda | DOID_3437 | D006685; D007827 | SYMP_0000068; SYMP_0000298; SYMP_0019181 | Hoarse Voice; Hoarseness; Laryngitis |
| Svarakarshya | DOID_0060046 | D001037 | SYMP_0000508 | Aphasia |
| Svaraksaya | DOID_0060046 | D001037 | SYMP_0000508 | Aphasia |
| Svasa | DOID_2841 | D001249; D004417 | SYMP_0019153 | Asthma; Dyspnea |
| Svayathu |  | D004487 | SYMP_0000538 | Edema |
| Svedadhikya |  | D006945 |  | Hyperhidrosis |
| Svitra | DOID_12306 | D014820 |  | Vitiligo |
| Swasa | DOID_2841 | D001249; D004417 | SYMP_0019153 | Asthma; Dyspnea |
| Tamaka Shvaasa | DOID_2841 | D001249; D004417 | SYMP_0019153 | Asthma; Dyspnea |
| Tamaka Svasa | DOID_2841 | D001249; D004417 | SYMP_0019153 | Asthma; Dyspnea |
| Timira | DOID_83 | D002386 |  | Cataract |
| Timira Roga | DOID_1432 | D001766 | SYMP_0000008 | Blindness |
| Trika Ruja |  | D017116 | SYMP_0000006 | Backache; Low Back Pain |
| Trika Shula |  | D017116 | SYMP_0000006 | Backache; Low Back Pain |
| Trika Sula |  | D017116 | SYMP_0000006 | Backache; Low Back Pain |
| Trikasula |  | D017116 | SYMP_0000006 | Backache; Low Back Pain |
| Trishna |  |  | SYMP_0000156 | Thirst |
| Trisna |  |  | SYMP_0000156 | Thirst |
| Trshna |  |  | SYMP_0000156 | Thirst |
| Trsna |  |  | SYMP_0000156 | Thirst |
| Trt |  |  | SYMP_0000156 | Thirst |
| Tuberculosis Of Hip Joint; Tuberculosis Of Spine | DOID_399 | D014375; D014376 |  | Tuberculoma; Tuberculosis |
| Tvagdosa | DOID_37 | D012873 |  | Skin Diseases, Genetic |
| Tvakrauksya | DOID_1697 | D007057 |  | Ichthyosis |
| Tvakroga | DOID_37 | D012873 |  | Skin Diseases, Genetic |
| Udakodara |  | D001201 | SYMP_0000526 | Ascites |
| Udarasula |  | D015746 | SYMP_0000457 | Abdominal Pain |
| Ugra Atisara |  | D003967 |  | Diarrhea |
| Unmada |  | D000087122 | SYMP_0000607 | Hallucination; Mania |
| Unmāda |  | D000087122 | SYMP_0000607 | Hallucination; Mania |
| Upadamsa | DOID_4166 | D002601 |  | Chancre |
| Upadamsaja Vrana | DOID_13778 | D002602 |  | Chancroid |
| Upadamsha | DOID_4166 | D002601 |  | Chancre |
| Upadansha Vrana | DOID_13778 | D002602 |  | Chancroid |
| Upadaæsa | DOID_4166 | D002601 |  | Chancre |
| Urahksata |  | D013898 |  | Thoracic Injuries |
| Urahkshata |  | D013898 |  | Thoracic Injuries |
| Uraåksata |  | D013898 |  | Thoracic Injuries |
| Urdhvasvasa |  | D059246 | SYMP_0000603 | Tachypnea |
| Uroroga | DOID_0060118 | D013896 |  | Thoracic Disease; Thoracic Diseases |
| Urushula |  | D010146 | SYMP_0000099 | Pain |
| Urusula |  | D010146 | SYMP_0000099 | Pain |
| Utklesha |  | D009325 | SYMP_0000458 | Nausea |
| Vakdosa | DOID_92 | D013064 |  | Speech Disorders |
| Vala | DOID_3144 | D003483 |  | Cutis Laxa |
| Vami |  | D014839 | SYMP_0019145 | Vomiting |
| Vandhyaroga | DOID_5223 | D007246 | SYMP_0000712 | Infertility |
| Vandhyatva | DOID_5223 | D007246 | SYMP_0000712 | Infertility |
| Varcovibandha | DOID_8448 | D003248 |  | Constipation; Intestinal Impaction |
| Vardhama | DOID_1283 | D006547 |  | Hernia |
| Varna Vikara | DOID_3156 | D010859 |  | Pigmentation Disorders |
| Varna Vikara; Vyanga | DOID_3156 | D010859 |  | Pigmentation Disorders |
| Varna Vikriti | DOID_3156 | D010859 |  | Pigmentation Disorders |
| Vartma Roga | DOID_530 | D005141 |  | Eyelid Diseases |
| Varyaksaya | DOID_14227 | D053713 |  | Azoospermia |
| Vastiroga | DOID_365 | D001745 |  | Urinary Bladder Diseases |
| Vatadhika Tamaka Swasa | DOID_2841 | D001249; D004417 | SYMP_0019153 | Asthma; Dyspnea |
| Vatagulma |  |  | SYMP_0000516; SYMP_0000799 | Abdominal Lump; Lump In Chest |
| Vataja Kasa |  | D003371 | SYMP_0000614 | Cough |
| Vatakaphaja Gulma |  |  | SYMP_0000799 | Abdominal Lump |
| Vatakaphajvara |  | D005334 | SYMP_0000613 | Fever |
| Vatarakta | DOID_13189 | D006073 |  | Gout |
| Vatasonita | DOID_13189 | D006073 |  | Gout |
| Vatavidradhi |  | D000038 | SYMP_0000672 | Abscess |
| Venereal Diseases |  | D012749 |  | Sexually Transmitted Diseases |
| Vibandha |  | D003248 |  | Constipation |
| Vicarcika |  | D004485 | SYMP_0000289 | Eczema |
| Vicharchika |  | D004485 | SYMP_0000289 | Eczema |
| Vidradhi |  | D000038 | SYMP_0000672 | Abscess |
| Viryaksaya | DOID_14227 | D053713 |  | Azoospermia |
| Visarpa | DOID_11330 | D004886 |  | Erysipelas |
| Vishaada |  |  | SYMP_0000412 | Anxiety |
| Vishavikara |  | D011041 |  | Poisoning |
| Vishoocika | DOID_2326 | D005759 | SYMP_0019159 | Gastroenteritis |
| Vishuchika | DOID_2326 | D005759 | SYMP_0019159 | Gastroenteritis |
| Vishucika | DOID_2326 | D005759 | SYMP_0019159 | Gastroenteritis |
| Visphota |  | D001768 | SYMP_0000009 | Blister |
| Vistambha |  | D003248 |  | Constipation |
| Visuchika | DOID_2326 | D005759 | SYMP_0019159 | Gastroenteritis |
| Visucika | DOID_2326 | D005759 | SYMP_0019159 | Gastroenteritis |
| Vitsanga |  | D003248 |  | Constipation |
| Vrana |  | D014456 |  | Ulcer |
| Vranasula |  | D010146 | SYMP_0000099 | Pain |
| Vranopacara |  | D014947 |  | Wounds And Injuries |
| Vrddhi | DOID_0060320 | D006552 |  | Hernia, Inguinal |
| Vriddhi | DOID_0060320 | D006552 |  | Hernia, Inguinal |
| Vrukkaroga | DOID_557 | D007674 |  | Kidney Diseases |
| Vyadhi Karshita Nastashukra | DOID_14228 | D009845 |  | Oligospermia |
| Vyanga | DOID_10123; DOID_3156 | D010859 |  | Pigmentation Disease; Pigmentation Disorders |
| Yakridroga | DOID_409 | D008107 |  | Liver Diseases |
| Yakrit | DOID_409 | D008107 |  | Liver Diseases |
| Yakrit Roga | DOID_409 | D008107 |  | Liver Diseases |
| Yakritpliharoga | DOID_2529; DOID_409 | D008107; D013158 |  | Liver Diseases; Splenic Diseases |
| Yakrtpliharoga | DOID_2529; DOID_409 | D008107; D013158 |  | Liver Diseases; Splenic Diseases |
| Yakrtplihodara | DOID_2529; DOID_409 | D008107; D013158 |  | Liver Diseases; Splenic Diseases |
| Yakrtroga | DOID_409 | D008107 |  | Liver Diseases |
| Yakrutplihodara | DOID_2529; DOID_409 | D008107; D013158 |  | Liver Diseases; Splenic Diseases |
| Yakshma | DOID_399 | D014375 | SYMP_0000174 | Tuberculoma; Wasting |
| Yaksma | DOID_399 | D014375; D014376 |  | Tuberculoma; Tuberculosis |
| Yoni Roga | DOID_229 | D005831 |  | Genital Diseases, Female |
| Yoni Vikara | DOID_121 | D014623 |  | Vaginal Diseases |
| Yonibhramsha | DOID_1284 |  |  | Prolapse Of Female Genital Organ |
| Yonidosa | DOID_229 | D005831 |  | Genital Diseases, Female |
| Yonidosha | DOID_229 | D005831 |  | Genital Diseases, Female |
| Yoniroga | DOID_229 | D005831 |  | Genital Diseases, Female |
| Yoniruja |  | D017699 |  | Pelvic Pain |
| Yonishula |  | D017699 |  | Pelvic Pain |
| Yonisula |  | D017699 |  | Pelvic Pain |
| Yoniśūla |  | D017699 |  | Pelvic Pain |
| Ādhmāna |  | D005414 | SYMP_0000420; SYMP_0000509 | Bloating; Flatulence |
| Śūla |  |  | SYMP_0000236 | Colicky Pain |

*Supplementary Table 3: Information on Benzylamine analogs from GRAPH traversal using Advanced Search*

|  | **Names** |
| --- | --- |
| **Benzyamine analogs** | Benzylamine (7504), N-Methylbenzylamine (7669), N,N-Dimethylbenzylamine (7681), 2-(Aminomethyl)phenol (70267), Bamipine (72075), Lavendustin C (3896), 4-Hydroxybenzylamine (97472) |
| **Plants containing these Phytochemicals** | Acanthospermum hispidum, Apium graveolens, Brassica oleracea, Camellia sinensis, Cestrum nocturnum, Cinnamomum glanduliferum, Cydonia oblonga, Daucus carota, Empetrum nigrum, Ephedra sinica, Erythroxylum coca, Fagopyrum esculentum, Malus domestica, Malus pumila, Moringa oleifera, Nama hispidum, Onosma hispidum, Piper acutifolium, Piper aduncum, Piper aequale, Piper angustifolium, Piper arboreum, Piper argyrophyllum, Piper attenuatum, Piper baccatum, Piper banksii, Piper bantamense, Piper barberi, Piper bavinum, Piper betel, Piper betle, Piper betleoides, Piper boehmeriaefolium, Piper brachystachyum, Piper callosum, Piper caninum, Piper chaba, Piper crassinervium, Piper cubeba, Piper diffusum, Piper dilatatum, Piper divaricatum, Piper elongatum, Piper futokadsura, Piper griffthii, Piper guineense, Piper hamiltonii, Piper hancei, Piper hispidum, Piper hookeri, Piper hostmannianum, Piper kadsura, Piper lanceifolium, Piper longum, Piper marginatum, Piper methysticum, Piper mullesua, Piper nepalens, Piper nigrum, Piper officinarum, Piper pedicellatum, Piper pedicellosum, Piper peepuloides, Piper polysyphorum, Piper puberulum, Piper regnellii, Piper retrofractum, Piper sanctum, Piper sarmentosum, Piper schmidtii, Piper spp, Piper sylvaticum, Piper taboganum, Piper taiwanense, Piper thomsonii, Piper trichostachyon, Piper trioicum, Piper tuberculatum, Piper umbellatum, Piper wallichii, Piper wightii, Populus tomentosa, Reseda odorata, Ribes nigrum, Salix babylonica, Solanum nigrum, Spinacia oleracea, Triumfetta rhomboidea, Veratrum nigrum, Viburnum prunifolium, Zea mays, Brassica juncea, Galeola faberi, Gastrodia elata, Sinapis alba, Urtica dioica |
| **Diseases associated with all these plants** | Gastroschisis, Chronic Bronchitis, Alopecia, Alopecia Areata, Amenorrhea, Atherosclerosis, Urolithiasis, Anemia, Hemolytic Anemia, Anorexia, Anuria, Penile Disease, Aphasia, Apnea, Appendicitis, Arteriosclerosis, Arthritis, Muscular Disease, Rheumatoid Arthritis, Urinary System Disease, Asthma, Bacterial Infections, Beriberi, Urinary Bladder Diseases, Urinary Bladder Neoplasms, Psychotic Disorder, Generalized Anxiety Disorder, Alzheimer'S Disease 1, Brain Diseases, Brain Edema, Bronchiectasis, Sexual Dysfunction, Bronchitis, Female Infertility Of Uterine Origin, Cardiovascular Diseases, Cataract, Celiac Disease, Central Nervous System Diseases, Rheumatic Disease, Uterine Cervical Neoplasms, Chancre, Chickenpox, Cholelithiasis, Cholera, Cholestasis, Common Wart, Common Cold, Conjunctivitis, Constipation, Coronary Artery Disease, Coronary Disease, Corynebacterium Infections, Crohn Disease, Cystic Fibrosis, Cystitis, Dementia, Dengue, Dental Caries, Dermatitis, Diabetes Mellitus, Type 1 Diabetes Mellitus, Digestive System Diseases, Duodenal Obstruction, Dysentery, Epilepsy, Erysipelas, Esophagitis, Eye Diseases, Eyelid Diseases, Facial Paralysis, Fibromyalgia, Filariasis, Gallbladder Diseases, Gastritis, Gastrointestinal Diseases, Glaucoma, Goiter, Gonorrhea, Gout, Heart Diseases, Hematologic Diseases, Hemorrhoids, Chronic Hepatitis, Viral Hepatitis, Animal, Herpes Simplex, Hypertension, Hypoglycemia, Hypohidrosis, Hypokalemia, Male Infertility, Human Influenza, Intestinal Diseases, Intestinal Obstruction, Joint Diseases, Kidney Diseases, Laryngeal Diseases, Laryngitis, Leprosy, Leukemia, Leukorrhea, Liver Diseases, Liver Neoplasms, Lung Diseases, Systemic Lupus Erythematosus, Lymphadenitis, Lymphoma, Malaria, Migraine Disorders, Mouth Diseases, Muscular Dystrophies, Musculoskeletal Diseases, Cardiomyopathies, Myocardial Infarction, Myopia, Neoplasms, Nephritis, Nervous System Diseases, Neuritis, Nose Diseases, Night Blindness, Obesity, Oligospermia, Endophthalmitis, Osteoarthritis, Osteoporosis, Otitis Media, Paraplegia, Peptic Ulcer, Familial Mediterranean Fever, Periodontal Diseases, Pharyngeal Diseases, Pleurisy, Pneumonia, Poliomyelitis, Pre-Eclampsia, Prostatitis, Psoriasis, Scabies, Systemic Scleroderma, Scurvy, Sinusitis, Skin Diseases, Genetic Skin Diseases, Skin Neoplasms, Smallpox, Splenic Diseases, Spondylitis, Stomach Diseases, Stomach Neoplasms, Stomatitis, Aphthous Stomatitis, Testicular Diseases, Tetanus, Thrombocytopenia, Thrombosis, Tongue Diseases, Tooth Diseases, Tuberculoma, Typhoid Fever, Urethritis, Urinary Tract Infections, Uterine Diseases, Vaginitis, Varicose Veins, Vision Disorders, Vitiligo, Gouty Arthritis, Inflammatory Bowel Diseases, Carotid Stenosis, Myocardial Ischemia, Hypophosphatemia, Acquired Immunodeficiency Syndrome, Addison Disease, Adenocarcinoma, Vaginal Discharge |

*Supplementary Table 4: Common Disease Connections between Myristica malabarica and Syzygium aromaticum*

| source_name | source_type | target_name | target_type | label | props_json |
| --- | --- | --- | --- | --- | --- |
| Myristica malabarica | Plant | Myocardial Infarction | Disease | ASSOCIATED_WITH_DISEASE | {"DOID":"DOID_5844","ICD11":"BA41;BA43","MESH":"D009203","association_by_therapeutic_target":"F2"} |
| Myristica malabarica | Plant | Common Cold | Disease | ASSOCIATED_WITH_DISEASE | {"DOID":"DOID_10459","ICD11":"CA00","MESH":"D003139","part":"fruit","reference":"ISBN:9770972795006"} |
| Myristica malabarica | Plant | Asthma | Disease | ASSOCIATED_WITH_DISEASE | {"DOID":"DOID_2841","ICD11":"CA23","MESH":"D001249","association_by_therapeutic_target":"F2"} |
| Myristica malabarica | Plant | Diabetes Mellitus | Disease | ASSOCIATED_WITH_DISEASE | {"DOID":"DOID_9351","ICD11":"5A14","MESH":"D003920","association_by_therapeutic_target":"RELA"} |
| Myristica malabarica | Plant | Crohn Disease | Disease | ASSOCIATED_WITH_DISEASE | {"DOID":"DOID_8778","ICD11":"DD70","MESH":"D003424","association_by_therapeutic_target":"RORC"} |
| Myristica malabarica | Plant | Cardiovascular Diseases | Disease | ASSOCIATED_WITH_DISEASE | {"DOID":"DOID_1287","ICD11":"BA00;BE2Z","MESH":"D002318","association_by_therapeutic_target":"F2;ALDH2"} |
| Myristica malabarica | Plant | Psoriasis | Disease | ASSOCIATED_WITH_DISEASE | {"DOID":"DOID_8893","ICD11":"EA90","MESH":"D011565","association_by_therapeutic_target":"RORC;RELA"} |
| Myristica malabarica | Plant | Dementia | Disease | ASSOCIATED_WITH_DISEASE | {"DOID":"DOID_1307","ICD11":"6D80;6D8Z","MESH":"D003704","association_by_therapeutic_target":"DRD1"} |
| Myristica malabarica | Plant | Arthritis, Rheumatoid | Disease | ASSOCIATED_WITH_DISEASE | {"DOID":"DOID_7148","ICD11":"FA20","MESH":"D001172","association_by_therapeutic_target":"RELA"} |
| Myristica malabarica | Plant | Nephritis | Disease | ASSOCIATED_WITH_DISEASE | {"DOID":"DOID_10952","ICD11":"GB40","MESH":"D009393","association_by_clinical_trials_of_plant_ingredients":"NCT02662283"} |
| Myristica malabarica | Plant | Bronchitis | Disease | ASSOCIATED_WITH_DISEASE | {"DOID":"DOID_6132","ICD11":"CA20","MESH":"D001991","part":"seed","reference":"ISBN:9788173717055"} |
| Myristica malabarica | Plant | Muscular Dystrophies | Disease | ASSOCIATED_WITH_DISEASE | {"DOID":"DOID_9884","ICD11":"8C70","MESH":"D009136","association_by_therapeutic_target":"RELA"} |
| Myristica malabarica | Plant | Liver Diseases | Disease | ASSOCIATED_WITH_DISEASE | {"DOID":"DOID_409","ICD11":"SA0Z","MESH":"D008107","part":"seed","reference":"ISBN:9770972795006"} |
| Myristica malabarica | Plant | Thrombocytopenia | Disease | ASSOCIATED_WITH_DISEASE | {"DOID":"DOID_1588","ICD11":"3B64","MESH":"D013921","association_by_therapeutic_target":"F2"} |
| Myristica malabarica | Plant | Psychotic Disorder | Disease | ASSOCIATED_WITH_DISEASE | {"DOID":"DOID_5419","ICD11":"6A20;6A25","MESH":"D012559","association_by_therapeutic_target":"DRD1"} |
| Syzygium aromaticum | Plant | Liver Diseases | Disease | ASSOCIATED_WITH_DISEASE | {"DOID":"DOID_409","ICD11":"SA0Z","MESH":"D008107","part":"flower","reference":"ISBN:9788173717062"} |
| Syzygium aromaticum | Plant | Psychotic Disorder | Disease | ASSOCIATED_WITH_DISEASE | {"DOID":"DOID_5419","ICD11":"6A20;6A25","MESH":"D012559","association_by_therapeutic_target":"GRM3;INSR;GRM2"} |
| Syzygium aromaticum | Plant | Nephritis | Disease | ASSOCIATED_WITH_DISEASE | {"DOID":"DOID_10952","ICD11":"GB40","MESH":"D009393","association_by_clinical_trials_of_plant_ingredients":"NCT00549692"} |
| Syzygium aromaticum | Plant | Muscular Dystrophies | Disease | ASSOCIATED_WITH_DISEASE | {"DOID":"DOID_9884","ICD11":"8C70","MESH":"D009136","association_by_therapeutic_target":"NFKB1;NR3C1;RELA;PTGS1"} |
| Syzygium aromaticum | Plant | Arthritis, Rheumatoid | Disease | ASSOCIATED_WITH_DISEASE | {"DOID":"DOID_7148","ICD11":"FA20","MESH":"D001172","association_by_clinical_trials_of_plant_ingredients":"NCT01850680;NCT02795299;NCT01116141;NCT02293902;NCT01061736;NCT01217814;NCT04535427;NCT00243412;NCT03028467;NCT00965653;NCT00613106;NCT00299130;NCT02930343;NCT01711359;NCT00578305;NCT05363917;NCT02093026;NCT00266227;NCT02762838;NCT02379091;NCT02504671;NCT00346216;NCT00299104;NCT00950989;NCT01873443;NCT01010581;NCT00298272;NCT02833350;NCT02393378;NCT00145730;NCT02467504;NCT03172325;NCT00072982","association_by_therapeutic_target":"ABCG2;RELA;AKR1B1;PTGS1;ALOX5;NFKB1;PTGS2;CA2;MMP9;MMP1;NR3C1"} |
| Syzygium aromaticum | Plant | Thrombocytopenia | Disease | ASSOCIATED_WITH_DISEASE | {"DOID":"DOID_1588","ICD11":"3B64","MESH":"D013921","association_by_clinical_trials_of_plant_ingredients":"NCT01014546;NCT00272610;NCT05025488","association_by_therapeutic_target":"F2;MIF;AHR;KDR;ALOX5"} |
| Syzygium aromaticum | Plant | Psoriasis | Disease | ASSOCIATED_WITH_DISEASE | {"DOID":"DOID_8893","ICD11":"EA90","MESH":"D011565","association_by_disease_transcriptome_reversion":"FABP5;AKR1B10","association_by_therapeutic_target":"ALOX5;DPP4;FLT3;AHR;TOP2A;RELA;RORC;NFKB1"} |
| Syzygium aromaticum | Plant | Cardiovascular Diseases | Disease | ASSOCIATED_WITH_DISEASE | {"DOID":"DOID_1287","ICD11":["BA00","BE2Z"],"MESH":"D002318","association_by_clinical_trials_of_plant_ingredients":["NCT02178410","NCT03679780","NCT02647333","NCT00408577"],"association_by_therapeutic_target":["HCAR2","F2","MET","PTGS2","SRC","KDR","PTGS1","PPARA"],"reference":"ISBN:9788172363093"} |
| Syzygium aromaticum | Plant | Diabetes Mellitus | Disease | ASSOCIATED_WITH_DISEASE | {"DOID":"DOID_9351","ICD11":"5A14","MESH":"D003920","association_by_clinical_trials_of_plant_ingredients":"NCT02107976;NCT02297399","association_by_therapeutic_target":"NFKB1;ESR1;MIF;INSR;PPARA;RELA;DPP4"} |
| Syzygium aromaticum | Plant | Bronchitis | Disease | ASSOCIATED_WITH_DISEASE | {"DOID":"DOID_6132","ICD11":"CA20","MESH":"D001991","part":"flower","reference":"ISBN:9788171360536; ISBN:9788172361150; ISBN:9788173717062"} |
| Syzygium aromaticum | Plant | Crohn Disease | Disease | ASSOCIATED_WITH_DISEASE | {"DOID":"DOID_8778","ICD11":"DD70","MESH":"D003424","association_by_clinical_trials_of_plant_ingredients":"NCT00275418","association_by_disease_transcriptome_reversion":"MMP9;GRIN3A;MMP1;HCAR2;PTGS2","association_by_therapeutic_target":"CNR2;INSR;RORC;MMP9"} |
| Syzygium aromaticum | Plant | Common Cold | Disease | ASSOCIATED_WITH_DISEASE | {"DOID":"DOID_10459","ICD11":"CA00","MESH":"D003139","part":"flower","reference":"ISBN:9788171360536; ISBN:9788172361150; ISBN:9788173717062"} |
| Syzygium aromaticum | Plant | Asthma | Disease | ASSOCIATED_WITH_DISEASE | {"DOID":"DOID_2841","ICD11":"CA23","MESH":"D001249","association_by_clinical_trials_of_plant_ingredients":"NCT01501942;NCT01057615;NCT00029679;NCT00280683;NCT00526357","association_by_therapeutic_target":"PTGS2;F2;TOP2A;NR3C1;ALOX5","part":"flower","reference":"ISBN:9788171360536; ISBN:9788173717062"} |
| Syzygium aromaticum | Plant | Myocardial Infarction | Disease | ASSOCIATED_WITH_DISEASE | {"DOID":"DOID_5844","ICD11":"BA41","MESH":"D009203","association_by_clinical_trials_of_plant_ingredients":"NCT02929888"} |
| Syzygium aromaticum | Plant | Dementia | Disease | ASSOCIATED_WITH_DISEASE | {"DOID":"DOID_1307","ICD11":"6D80;6D8Z","MESH":"D003704","association_by_therapeutic_target":"ACHE;SLC6A3"} |

*Supplementary Table 5: Hub plants (10) and associated phytochemicals (20) identified from Ayurvedic formulations (17) for anemia in GRAYU.*

| **No.** | **Formulations** | ***Piper longum*** | ***Terminalia chebula*** | ***Piper nigrum*** | ***Zingiber officinale*** | ***Phyllanthus emblica*** | ***Terminalia bellirica*** | ***Coriandrum sativum*** | ***Woodfordia fruticosa*** | ***Elettaria cardamomum*** | ***Embelia ribes*** |
| --- | --- | --- | --- | --- | --- | --- | --- | --- | --- | --- | --- |
| 1 | Dasamularista | Yes | Yes | – | – | Yes | Yes | Yes | Yes | Yes | Yes |
| 2 | Dasamulasatpalaka Ghrta | Yes | – | – | Yes | – | – | – | – | – | – |
| 3 | Draksasava | Yes | – | – | – | – | – | – | Yes | Yes | – |
| 4 | Draksavaleha | Yes | – | – | Yes | Yes | – | Yes | – | – | – |
| 5 | Gomutra Haritaki | – | Yes | – | – | – | – | – | – | – | – |
| 6 | Guggulu Kaisora Guggulu | Yes | Yes | Yes | Yes | Yes | Yes | – | – | – | Yes |
| 7 | Kalyanaka Ghrta | – | Yes | – | – | Yes | Yes | – | – | Yes | Yes |
| 8 | Khadirarista | Yes | Yes | – | – | Yes | Yes | – | Yes | Yes | – |
| 9 | Lohasava | Yes | Yes | Yes | Yes | Yes | Yes | Yes | Yes | – | Yes |
| 10 | Narasimha Curna | Yes | – | Yes | Yes | – | – | Yes | – | – | – |
| 11 | Navayasa Curna | Yes | Yes | Yes | Yes | Yes | Yes | – | – | – | Yes |
| 12 | Pippalyadyasava | Yes | – | Yes | – | – | – | – | Yes | Yes | Yes |
| 13 | Puga Khanda | Yes | – | Yes | Yes | – | – | Yes | – | Yes | – |
| 14 | Punarnavasava | Yes | Yes | Yes | Yes | – | Yes | Yes | Yes | – | – |
| 15 | Simhanada Guggulu | – | Yes | – | – | Yes | Yes | – | – | – | – |
| 16 | Talisadya Curna | Yes | – | Yes | Yes | – | – | – | – | Yes | – |
| 17 | Usirasava | – | – | Yes | – | – | – | Yes | Yes | – | – |
|  | **Phytochemicals** |  |  |  |  |  |  |  |  |  |  |
| 1 | Palmitic Acid | Yes | Yes | Yes | Yes | Yes | Yes | Yes | Yes | Yes | Yes |
| 2 | Beta-Sitosterol | Yes | Yes | Yes | Yes | Yes | Yes | Yes | Yes | Yes | Yes |
| 3 | Stearic Acid | Yes | Yes | Yes | Yes | Yes | Yes | Yes | Yes | Yes | Yes |
| 4 | Nicotinic Acid | Yes | Yes | Yes | Yes | Yes | – | Yes | Yes | Yes | Yes |
| 5 | Alpha-Tocopherol (Vit E) | Yes | Yes | Yes | Yes | – | Yes | Yes | Yes | Yes | Yes |
| 6 | Rutin | Yes | Yes | Yes | Yes | Yes | Yes | Yes | Yes | – | – |
| 7 | Linoleic Acid | – | Yes | Yes | Yes | Yes | Yes | Yes | Yes | Yes | – |
| 8 | Ascorbic Acid (Vit C) | – | Yes | Yes | Yes | Yes | Yes | Yes | Yes | Yes | – |
| 9 | Oleic Acid | – | Yes | Yes | Yes | Yes | Yes | Yes | Yes | Yes | – |
| 10 | Elaidic Acid | Yes | Yes | Yes | Yes | – | Yes | Yes | – | Yes | Yes |
| 11 | Caryophyllene | Yes | Yes | Yes | Yes | Yes | – | Yes | – | Yes | Yes |
| 12 | Isoquercetin | Yes | – | Yes | Yes | – | – | Yes | Yes | – | Yes |
| 13 | Eucalyptol | Yes | – | Yes | Yes | – | – | Yes | Yes | Yes | – |
| 14 | Triacontane | Yes | Yes | – | Yes | – | Yes | Yes | Yes | – | – |
| 15 | Kaempferol | Yes | – | Yes | Yes | Yes | – | – | Yes | – | Yes |
| 16 | Riboflavin (Vit B₂) | – | – | Yes | Yes | Yes | – | Yes | Yes | Yes | – |
| 17 | D-Galactose | Yes | – | Yes | Yes | – | Yes | Yes | Yes | – | – |
| 18 | Chinese Gallotannin | – | Yes | Yes | Yes | Yes | Yes | – | Yes | – | – |
| 19 | Gallic Acid | – | Yes | – | Yes | Yes | Yes | Yes | Yes | – | – |
| 20 | Myrcene | Yes | – | Yes | Yes | Yes | – | Yes | – | Yes | – |

*Supplementary Table 6: All connections referred to in Fig 3b*

| source_name | source_type | target_name | target_type | label | props_json |
| --- | --- | --- | --- | --- | --- |
| Talisadya Curna | Formulation | Anemia | Disease | ASSOCIATED_WITH | {"source_db":"Ayurvedic Standard Treatment Guidelines; Ayurvedic Pharmacopeia of India"} |
| Usirasava | Formulation | Anemia | Disease | ASSOCIATED_WITH | {"source_db":"Ayurvedic Standard Treatment Guidelines; Ayurvedic Pharmacopeia of India"} |
| Puga Khanda | Formulation | Anemia | Disease | ASSOCIATED_WITH | {"source_db":"Ayurvedic Standard Treatment Guidelines; Ayurvedic Pharmacopeia of India"} |
| Simhanada Guggulu | Formulation | Anemia | Disease | ASSOCIATED_WITH | {"source_db":"Ayurvedic Standard Treatment Guidelines; Ayurvedic Pharmacopeia of India"} |
| Lohasava | Formulation | Anemia | Disease | ASSOCIATED_WITH | {"source_db":"Ayurvedic Standard Treatment Guidelines; Ayurvedic Pharmacopeia of India"} |
| Navayasa Curna | Formulation | Anemia | Disease | ASSOCIATED_WITH | {"source_db":"Ayurvedic Standard Treatment Guidelines; Ayurvedic Pharmacopeia of India"} |
| Draksavaleha | Formulation | Anemia | Disease | ASSOCIATED_WITH | {"source_db":"Ayurvedic Standard Treatment Guidelines; Ayurvedic Pharmacopeia of India"} |
| Kalyanaka Ghrta | Formulation | Anemia | Disease | ASSOCIATED_WITH | {"source_db":"Ayurvedic Standard Treatment Guidelines; Ayurvedic Pharmacopeia of India"} |
| Dasamularista | Formulation | Anemia | Disease | ASSOCIATED_WITH | {"source_db":"Ayurvedic Standard Treatment Guidelines; Ayurvedic Pharmacopeia of India"} |
| Dasamulasatpalaka Ghrta | Formulation | Anemia | Disease | ASSOCIATED_WITH | {"source_db":"Ayurvedic Standard Treatment Guidelines; Ayurvedic Pharmacopeia of India"} |
| Gomutra Haritaki | Formulation | Anemia | Disease | ASSOCIATED_WITH | {"source_db":"Ayurvedic Standard Treatment Guidelines; Ayurvedic Pharmacopeia of India"} |
| Draksasava | Formulation | Anemia | Disease | ASSOCIATED_WITH | {"source_db":"Ayurvedic Standard Treatment Guidelines; Ayurvedic Pharmacopeia of India"} |
| Guggulu Kaisora Guggulu | Formulation | Anemia | Disease | ASSOCIATED_WITH | {"source_db":"Ayurvedic Standard Treatment Guidelines; Ayurvedic Pharmacopeia of India"} |
| Punarnavasava | Formulation | Anemia | Disease | ASSOCIATED_WITH | {"source_db":"Ayurvedic Standard Treatment Guidelines; Ayurvedic Pharmacopeia of India"} |
| Narasimha Curna | Formulation | Anemia | Disease | ASSOCIATED_WITH | {"source_db":"Ayurvedic Standard Treatment Guidelines; Ayurvedic Pharmacopeia of India"} |
| Pippalyadyasava | Formulation | Anemia | Disease | ASSOCIATED_WITH | {"source_db":"Ayurvedic Standard Treatment Guidelines; Ayurvedic Pharmacopeia of India"} |
| Khadirarista | Formulation | Anemia | Disease | ASSOCIATED_WITH | {"source_db":"Ayurvedic Standard Treatment Guidelines; Ayurvedic Pharmacopeia of India"} |
| Piper longum | Plant | Narasimha Curna | Formulation | IS_INGREDIENT_IN | {"match_type":"synonym_exact","matched_synonym":"Piper longum","part":"Fruit","pharmacopoeia_ref":"Pippali(Fruit)","quantity":"128 g","source_db":"Ayurvedic Standard Treatment Guidelines; Ayurvedic Pharmacopeia of India"} |
| Piper longum | Plant | Guggulu Kaisora Guggulu | Formulation | IS_INGREDIENT_IN | {"match_type":"synonym_exact","matched_synonym":"Piper longum","part":"Fruit","pharmacopoeia_ref":"Pippali(Fruit)","quantity":"24 g","source_db":"Ayurvedic Pharmacopeia of India"} |
| Piper longum | Plant | Talisadya Curna | Formulation | IS_INGREDIENT_IN | {"match_type":"synonym_exact","matched_synonym":"Piper longum","part":"Fruit","pharmacopoeia_ref":"Pippali(Fruit)","quantity":"48 g","source_db":"Ayurvedic Pharmacopeia of India"} |
| Piper longum | Plant | Lohasava | Formulation | IS_INGREDIENT_IN | {"match_type":"synonym_exact","matched_synonym":"Piper longum","part":"Fruit","pharmacopoeia_ref":"Pippali(Fruit)","quantity":"192 g","source_db":"Ayurvedic Standard Treatment Guidelines; Ayurvedic Pharmacopeia of India; Ayurvedic Formulary of India"} |
| Piper longum | Plant | Dasamulasatpalaka Ghrta | Formulation | IS_INGREDIENT_IN | {"match_type":"synonym_exact","matched_synonym":"Piper longum","part":"Root","pharmacopoeia_ref":"Pippalimula(Stem)","quantity":"21.33 g","source_db":"Ayurvedic Standard Treatment Guidelines; Ayurvedic Pharmacopeia of India"} |
| Piper longum | Plant | Khadirarista | Formulation | IS_INGREDIENT_IN | {"match_type":"synonym_exact","matched_synonym":"Piper longum","part":"Fruit","pharmacopoeia_ref":"Pippali(Fruit)","quantity":"192 g","source_db":"Ayurvedic Standard Treatment Guidelines; Ayurvedic Pharmacopeia of India; Ayurvedic Formulary of India"} |
| Piper longum | Plant | Dasamularista | Formulation | IS_INGREDIENT_IN | {"match_type":"synonym_exact","matched_synonym":"Piper longum","part":"Fruit","pharmacopoeia_ref":"Pippali(Fruit)","quantity":"19 g","source_db":"Ayurvedic Standard Treatment Guidelines; Ayurvedic Pharmacopeia of India"} |
| Piper longum | Plant | Puga Khanda | Formulation | IS_INGREDIENT_IN | {"match_type":"synonym_exact","matched_synonym":"Piper longum","part":"Fruit","pharmacopoeia_ref":"Pippali(Fruit)","quantity":"24 g","source_db":"Ayurvedic Pharmacopeia of India"} |
| Piper longum | Plant | Punarnavasava | Formulation | IS_INGREDIENT_IN | {"match_type":"synonym_exact","matched_synonym":"Piper longum","part":"Fruit","pharmacopoeia_ref":"Pippali(Fruit)","quantity":"16 g","source_db":"Ayurvedic Standard Treatment Guidelines; Ayurvedic Pharmacopeia of India; Ayurvedic Formulary of India"} |
| Piper longum | Plant | Pippalyadyasava | Formulation | IS_INGREDIENT_IN | {"match_type":"synonym_exact","matched_synonym":"Piper longum","part":"Fruit","pharmacopoeia_ref":"Pippali(Fruit)","quantity":"8g","source_db":"Ayurvedic Standard Treatment Guidelines; Ayurvedic Pharmacopeia of India; Ayurvedic Formulary of India"} |
| Piper longum | Plant | Draksasava | Formulation | IS_INGREDIENT_IN | {"match_type":"synonym_exact","matched_synonym":"Piper longum","part":"Fruit","pharmacopoeia_ref":"Pippali(Fruit)","quantity":"24 g","source_db":"Ayurvedic Standard Treatment Guidelines; Ayurvedic Pharmacopeia of India"} |
| Piper longum | Plant | Navayasa Curna | Formulation | IS_INGREDIENT_IN | {"match_type":"synonym_exact","matched_synonym":"Piper longum","part":"Fruit","pharmacopoeia_ref":"Pippali(Fruit)","quantity":"1 part","source_db":"Ayurvedic Pharmacopeia of India"} |
| Piper longum | Plant | Draksavaleha | Formulation | IS_INGREDIENT_IN | {"match_type":"synonym_exact","matched_synonym":"Piper longum","part":"Fruit","pharmacopoeia_ref":"Pippali(Fruit)","quantity":"768 g","source_db":"Ayurvedic Standard Treatment Guidelines; Ayurvedic Pharmacopeia of India"} |
| Piper longum | Plant | Anemia | Disease | ASSOCIATED_WITH_DISEASE | {"DOID":"DOID_2355","ICD11":"3A9Z","MESH":"D000740","part":"fruit","reference":"ISBN:9780387706375; ISBN:81-204-0828-4"} |
| Embelia ribes | Plant | Navayasa Curna | Formulation | IS_INGREDIENT_IN | {"match_type":"synonym_exact","matched_synonym":"Embelia ribes","part":"Fruit","pharmacopoeia_ref":"Vidanga(Fruit)","quantity":"1 part","source_db":"Ayurvedic Pharmacopeia of India"} |
| Embelia ribes | Plant | Guggulu Kaisora Guggulu | Formulation | IS_INGREDIENT_IN | {"match_type":"synonym_exact","matched_synonym":"Embelia ribes","part":"Fruit","pharmacopoeia_ref":"Vidanga(Fruit)","quantity":"24 g","source_db":"Ayurvedic Pharmacopeia of India"} |
| Embelia ribes | Plant | Kalyanaka Ghrta | Formulation | IS_INGREDIENT_IN | {"match_type":"synonym_exact","matched_synonym":"Embelia ribes","part":"Fruit","pharmacopoeia_ref":"Vidanga(Fruit)","quantity":"12 g","source_db":"Ayurvedic Standard Treatment Guidelines; Ayurvedic Pharmacopeia of India"} |
| Embelia ribes | Plant | Lohasava | Formulation | IS_INGREDIENT_IN | {"match_type":"synonym_exact","matched_synonym":"Embelia ribes","part":"Fruit","pharmacopoeia_ref":"Vidanga(Fruit)","quantity":"192 g","source_db":"Ayurvedic Standard Treatment Guidelines; Ayurvedic Pharmacopeia of India; Ayurvedic Formulary of India"} |
| Embelia ribes | Plant | Dasamularista | Formulation | IS_INGREDIENT_IN | {"match_type":"synonym_exact","matched_synonym":"Embelia ribes","part":"Fruit","pharmacopoeia_ref":"Vidanga(Fruit)","quantity":"19 g","source_db":"Ayurvedic Standard Treatment Guidelines; Ayurvedic Pharmacopeia of India"} |
| Embelia ribes | Plant | Pippalyadyasava | Formulation | IS_INGREDIENT_IN | {"match_type":"synonym_exact","matched_synonym":"Embelia ribes","part":"Fruit","pharmacopoeia_ref":"Vidanga(Fruit)","quantity":"8g","source_db":"Ayurvedic Standard Treatment Guidelines; Ayurvedic Pharmacopeia of India; Ayurvedic Formulary of India"} |
| Embelia ribes | Plant | Anemia | Disease | ASSOCIATED_WITH_DISEASE | {"DOID":"DOID_2355","ICD11":"3A9Z","MESH":"D000740","part":"fruit; seed","reference":"ISBN:9788172361150; ISBN:9780387706375"} |
| Terminalia chebula | Plant | Dasamularista | Formulation | IS_INGREDIENT_IN | {"match_type":"synonym_exact","matched_synonym":"Terminalia chebula","part":"Pericarp","pharmacopoeia_ref":"Haritaki(Fruit)","quantity":"77g","source_db":"Ayurvedic Standard Treatment Guidelines; Ayurvedic Pharmacopeia of India"} |
| Terminalia chebula | Plant | Gomutra Haritaki | Formulation | IS_INGREDIENT_IN | {"match_type":"synonym_exact","matched_synonym":"Terminalia chebula","part":"Pericarp","pharmacopoeia_ref":"Haritaki(Fruit)","quantity":"1 part","source_db":"Ayurvedic Standard Treatment Guidelines; Ayurvedic Pharmacopeia of India"} |
| Terminalia chebula | Plant | Khadirarista | Formulation | IS_INGREDIENT_IN | {"match_type":"synonym_exact","matched_synonym":"Terminalia chebula","part":"Pericarp","pharmacopoeia_ref":"Haritaki(Fruit)","quantity":"960 g","source_db":"Ayurvedic Standard Treatment Guidelines; Ayurvedic Pharmacopeia of India; Ayurvedic Formulary of India"} |
| Terminalia chebula | Plant | Guggulu Kaisora Guggulu | Formulation | IS_INGREDIENT_IN | {"match_type":"synonym_exact","matched_synonym":"Terminalia chebula","part":"Pericarp","pharmacopoeia_ref":"Haritaki(Fruit)","quantity":"8g","source_db":"Ayurvedic Pharmacopeia of India"} |
| Terminalia chebula | Plant | Punarnavasava | Formulation | IS_INGREDIENT_IN | {"match_type":"synonym_exact","matched_synonym":"Terminalia chebula","part":"Fr.P","pharmacopoeia_ref":"Haritaki(Fruit)","quantity":"16 g","source_db":"Ayurvedic Standard Treatment Guidelines; Ayurvedic Pharmacopeia of India; Ayurvedic Formulary of India"} |
| Terminalia chebula | Plant | Kalyanaka Ghrta | Formulation | IS_INGREDIENT_IN | {"match_type":"synonym_exact","matched_synonym":"Terminalia chebula","part":"Pericarp","pharmacopoeia_ref":"Haritaki(Fruit)","quantity":"12 g","source_db":"Ayurvedic Standard Treatment Guidelines; Ayurvedic Pharmacopeia of India"} |
| Terminalia chebula | Plant | Simhanada Guggulu | Formulation | IS_INGREDIENT_IN | {"match_type":"synonym_exact","matched_synonym":"Terminalia chebula","part":"Pericarp","pharmacopoeia_ref":"Haritaki(Fruit)","quantity":"48 g","source_db":"Ayurvedic Standard Treatment Guidelines; Ayurvedic Pharmacopeia of India"} |
| Terminalia chebula | Plant | Navayasa Curna | Formulation | IS_INGREDIENT_IN | {"match_type":"synonym_exact","matched_synonym":"Terminalia chebula","part":"Pericarp","pharmacopoeia_ref":"Haritaki(Fruit)","quantity":"1 part","source_db":"Ayurvedic Pharmacopeia of India"} |
| Terminalia chebula | Plant | Lohasava | Formulation | IS_INGREDIENT_IN | {"match_type":"synonym_exact","matched_synonym":"Terminalia chebula","part":"Pericarp","pharmacopoeia_ref":"Haritaki(Fruit)","quantity":"192 g","source_db":"Ayurvedic Standard Treatment Guidelines; Ayurvedic Pharmacopeia of India; Ayurvedic Formulary of India"} |
| Terminalia chebula | Plant | Anemia | Disease | ASSOCIATED_WITH_DISEASE | {"DOID":"DOID_2355","ICD11":"3A9Z;3A00","MESH":"D000740","association_by_clinical_trials_of_plant_ingredients":"NCT02780505;NCT02225886;NCT02631668;NCT00920413","part":"fruit","reference":"ISBN:81-204-0828-4; ISBN:9780387706375; ISBN:9788171360536; ISBN:9788172363093; ISBN:9788190648943"} |
| Piper nigrum | Plant | Talisadya Curna | Formulation | IS_INGREDIENT_IN | {"match_type":"synonym_exact","matched_synonym":"Piper nigrum","part":"Fruit","pharmacopoeia_ref":"Marica(Fruit)","quantity":"24 g","source_db":"Ayurvedic Pharmacopeia of India"} |
| Piper nigrum | Plant | Usirasava | Formulation | IS_INGREDIENT_IN | {"match_type":"synonym_exact","matched_synonym":"Piper nigrum","part":"Fruit","pharmacopoeia_ref":"Marica(Fruit)","quantity":"q.s. for dhupana","source_db":"Ayurvedic Standard Treatment Guidelines; Ayurvedic Pharmacopeia of India"} |
| Piper nigrum | Plant | Puga Khanda | Formulation | IS_INGREDIENT_IN | {"match_type":"synonym_exact","matched_synonym":"Piper nigrum","part":"Fruit","pharmacopoeia_ref":"Marica(Fruit)","quantity":"24 g","source_db":"Ayurvedic Pharmacopeia of India"} |
| Piper nigrum | Plant | Punarnavasava | Formulation | IS_INGREDIENT_IN | {"match_type":"synonym_exact","matched_synonym":"Piper nigrum","part":"Fruit","pharmacopoeia_ref":"Marica(Fruit)","quantity":"16 g","source_db":"Ayurvedic Standard Treatment Guidelines; Ayurvedic Pharmacopeia of India; Ayurvedic Formulary of India"} |
| Piper nigrum | Plant | Guggulu Kaisora Guggulu | Formulation | IS_INGREDIENT_IN | {"match_type":"synonym_exact","matched_synonym":"Piper nigrum","part":"Fruit","pharmacopoeia_ref":"Marica(Fruit)","quantity":"24 g","source_db":"Ayurvedic Pharmacopeia of India"} |
| Piper nigrum | Plant | Pippalyadyasava | Formulation | IS_INGREDIENT_IN | {"match_type":"synonym_exact","matched_synonym":"Piper nigrum","part":"Fruit","pharmacopoeia_ref":"Marica(Fruit)","quantity":"8g","source_db":"Ayurvedic Standard Treatment Guidelines; Ayurvedic Pharmacopeia of India; Ayurvedic Formulary of India"} |
| Piper nigrum | Plant | Navayasa Curna | Formulation | IS_INGREDIENT_IN | {"match_type":"synonym_exact","matched_synonym":"Piper nigrum","part":"Fruit","pharmacopoeia_ref":"Marica(Fruit)","quantity":"1 part","source_db":"Ayurvedic Pharmacopeia of India"} |
| Piper nigrum | Plant | Narasimha Curna | Formulation | IS_INGREDIENT_IN | {"match_type":"synonym_exact","matched_synonym":"Piper nigrum","part":"Fruit","pharmacopoeia_ref":"Marica(Fruit)","quantity":"128 g","source_db":"Ayurvedic Standard Treatment Guidelines; Ayurvedic Pharmacopeia of India"} |
| Piper nigrum | Plant | Lohasava | Formulation | IS_INGREDIENT_IN | {"match_type":"synonym_exact","matched_synonym":"Piper nigrum","part":"Fruit","pharmacopoeia_ref":"Marica(Fruit)","quantity":"192 g","source_db":"Ayurvedic Standard Treatment Guidelines; Ayurvedic Pharmacopeia of India; Ayurvedic Formulary of India"} |
| Piper nigrum | Plant | Anemia | Disease | ASSOCIATED_WITH_DISEASE | {"DOID":"DOID_2355","ICD11":"3A9Z;3A00","MESH":"D000740","association_by_clinical_trials_of_plant_ingredients":"NCT00920413;NCT02631668;NCT02225886;NCT01490944;NCT00953134;NCT01670955;NCT02780505"} |
| Zingiber officinale | Plant | Lohasava | Formulation | IS_INGREDIENT_IN | {"match_type":"synonym_exact","matched_synonym":"Zingiber officinale","part":"Rhizome","pharmacopoeia_ref":"Sunthi(Rhizome)","quantity":"192 g","source_db":"Ayurvedic Standard Treatment Guidelines; Ayurvedic Pharmacopeia of India; Ayurvedic Formulary of India"} |
| Zingiber officinale | Plant | Dasamulasatpalaka Ghrta | Formulation | IS_INGREDIENT_IN | {"match_type":"synonym_exact","matched_synonym":"Zingiber officinale","part":"Rhizome","pharmacopoeia_ref":"Sunthi(Rhizome)","quantity":"21.33 g","source_db":"Ayurvedic Standard Treatment Guidelines; Ayurvedic Pharmacopeia of India"} |
| Zingiber officinale | Plant | Punarnavasava | Formulation | IS_INGREDIENT_IN | {"match_type":"synonym_exact","matched_synonym":"Zingiber officinale","part":"Rhizome","pharmacopoeia_ref":"Sunthi(Rhizome)","quantity":"16 g","source_db":"Ayurvedic Standard Treatment Guidelines; Ayurvedic Pharmacopeia of India; Ayurvedic Formulary of India"} |
| Zingiber officinale | Plant | Talisadya Curna | Formulation | IS_INGREDIENT_IN | {"match_type":"synonym_exact","matched_synonym":"Zingiber officinale","part":"Rhizome","pharmacopoeia_ref":"Sunthi(Rhizome)","quantity":"36 g","source_db":"Ayurvedic Pharmacopeia of India"} |
| Zingiber officinale | Plant | Guggulu Kaisora Guggulu | Formulation | IS_INGREDIENT_IN | {"match_type":"synonym_exact","matched_synonym":"Zingiber officinale","part":"Rhizome","pharmacopoeia_ref":"Sunthi(Rhizome)","quantity":"24 g","source_db":"Ayurvedic Pharmacopeia of India"} |
| Zingiber officinale | Plant | Draksavaleha | Formulation | IS_INGREDIENT_IN | {"match_type":"synonym_exact","matched_synonym":"Zingiber officinale","part":"Rhizome","pharmacopoeia_ref":"Sunthi(Rhizome)","quantity":"96 g","source_db":"Ayurvedic Standard Treatment Guidelines; Ayurvedic Pharmacopeia of India"} |
| Zingiber officinale | Plant | Puga Khanda | Formulation | IS_INGREDIENT_IN | {"match_type":"synonym_exact","matched_synonym":"Zingiber officinale","part":"Rhizome","pharmacopoeia_ref":"Sunthi(Rhizome)","quantity":"24 g","source_db":"Ayurvedic Pharmacopeia of India"} |
| Zingiber officinale | Plant | Narasimha Curna | Formulation | IS_INGREDIENT_IN | {"match_type":"synonym_exact","matched_synonym":"Zingiber officinale","part":"Rhizome","pharmacopoeia_ref":"Sunthi(Rhizome)","quantity":"128 g","source_db":"Ayurvedic Standard Treatment Guidelines; Ayurvedic Pharmacopeia of India"} |
| Zingiber officinale | Plant | Navayasa Curna | Formulation | IS_INGREDIENT_IN | {"match_type":"synonym_exact","matched_synonym":"Zingiber officinale","part":"Rhizome","pharmacopoeia_ref":"Sunthi(Rhizome)","quantity":"1 part","source_db":"Ayurvedic Pharmacopeia of India"} |
| Zingiber officinale | Plant | Anemia | Disease | ASSOCIATED_WITH_DISEASE | {"DOID":"DOID_2355","ICD11":"3A9Z;3A00","MESH":"D000740","association_by_clinical_trials_of_plant_ingredients":"NCT00953134;NCT02780505;NCT01490944;NCT01670955;NCT00920413;NCT02631668;NCT02225886","part":"rhizome","reference":"The Ayurvedic Pharmacopoeia of India Part-1 Volume-9; ISBN:81-204-0828-4"} |
| Terminalia bellirica | Plant | Dasamularista | Formulation | IS_INGREDIENT_IN | {"match_type":"synonym_exact","matched_synonym":"Terminalia bellirica","part":"Pericarp","pharmacopoeia_ref":"Bibhitaka(Fruit)","quantity":"19 g","source_db":"Ayurvedic Standard Treatment Guidelines; Ayurvedic Pharmacopeia of India"} |
| Terminalia bellirica | Plant | Lohasava | Formulation | IS_INGREDIENT_IN | {"match_type":"synonym_exact","matched_synonym":"Terminalia bellirica","part":"Pericarp","pharmacopoeia_ref":"Bibhitaka(Fruit)","quantity":"192 g","source_db":"Ayurvedic Standard Treatment Guidelines; Ayurvedic Pharmacopeia of India; Ayurvedic Formulary of India"} |
| Terminalia bellirica | Plant | Guggulu Kaisora Guggulu | Formulation | IS_INGREDIENT_IN | {"match_type":"synonym_exact","matched_synonym":"Terminalia bellirica","part":"Pericarp","pharmacopoeia_ref":"Bibhitaka(Fruit)","quantity":"8g","source_db":"Ayurvedic Pharmacopeia of India"} |
| Terminalia bellirica | Plant | Kalyanaka Ghrta | Formulation | IS_INGREDIENT_IN | {"match_type":"synonym_exact","matched_synonym":"Terminalia bellirica","part":"Pericarp","pharmacopoeia_ref":"Bibhitaka(Fruit)","quantity":"12 g","source_db":"Ayurvedic Standard Treatment Guidelines; Ayurvedic Pharmacopeia of India"} |
| Terminalia bellirica | Plant | Navayasa Curna | Formulation | IS_INGREDIENT_IN | {"match_type":"synonym_exact","matched_synonym":"Terminalia bellirica","part":"Pericarp","pharmacopoeia_ref":"Bibhitaka(Fruit)","quantity":"1 part","source_db":"Ayurvedic Pharmacopeia of India"} |
| Terminalia bellirica | Plant | Punarnavasava | Formulation | IS_INGREDIENT_IN | {"match_type":"synonym_exact","matched_synonym":"Terminalia bellirica","part":"Fr.P","pharmacopoeia_ref":"Bibhitaka(Fruit)","quantity":"16 g","source_db":"Ayurvedic Standard Treatment Guidelines; Ayurvedic Pharmacopeia of India; Ayurvedic Formulary of India"} |
| Terminalia bellirica | Plant | Khadirarista | Formulation | IS_INGREDIENT_IN | {"match_type":"synonym_exact","matched_synonym":"Terminalia bellirica","part":"Pericarp","pharmacopoeia_ref":"Bibhitaka(Fruit)","quantity":"960 g","source_db":"Ayurvedic Standard Treatment Guidelines; Ayurvedic Pharmacopeia of India; Ayurvedic Formulary of India"} |
| Terminalia bellirica | Plant | Simhanada Guggulu | Formulation | IS_INGREDIENT_IN | {"match_type":"synonym_exact","matched_synonym":"Terminalia bellirica","part":"Pericarp","pharmacopoeia_ref":"Bibhitaka(Fruit)","quantity":"48 g","source_db":"Ayurvedic Standard Treatment Guidelines; Ayurvedic Pharmacopeia of India"} |
| Terminalia bellirica | Plant | Anemia | Disease | ASSOCIATED_WITH_DISEASE | {"DOID":"DOID_2355","ICD11":"3A9Z;3A00","MESH":"D000740","association_by_clinical_trials_of_plant_ingredients":"NCT02780505;NCT02631668;NCT00920413;NCT02225886","part":"bark; fruit","reference":"ISBN:9788171360536; ISBN:9788173717062; ISBN:81-204-0828-4; ISBN:9788172363093"} |
| Coriandrum sativum | Plant | Dasamularista | Formulation | IS_INGREDIENT_IN | {"match_type":"synonym_contains","matched_synonym":"Dhoney","pharmacopoeia_ref":"Madhu(-)","quantity":"307 g","source_db":"Ayurvedic Standard Treatment Guidelines; Ayurvedic Pharmacopeia of India"} |
| Coriandrum sativum | Plant | Usirasava | Formulation | IS_INGREDIENT_IN | {"match_type":"synonym_contains","matched_synonym":"Dhoney","pharmacopoeia_ref":"Madhu(-)","quantity":"4.8 kg","source_db":"Ayurvedic Standard Treatment Guidelines; Ayurvedic Pharmacopeia of India"} |
| Coriandrum sativum | Plant | Narasimha Curna | Formulation | IS_INGREDIENT_IN | {"match_type":"synonym_contains","matched_synonym":"Dhoney","pharmacopoeia_ref":"Madhu(-)","quantity":"1.680 kg","source_db":"Ayurvedic Standard Treatment Guidelines; Ayurvedic Pharmacopeia of India"} |
| Coriandrum sativum | Plant | Punarnavasava | Formulation | IS_INGREDIENT_IN | {"match_type":"synonym_contains","matched_synonym":"Dhoney","pharmacopoeia_ref":"Madhu(-)","quantity":"800 g","source_db":"Ayurvedic Standard Treatment Guidelines; Ayurvedic Pharmacopeia of India; Ayurvedic Formulary of India"} |
| Coriandrum sativum | Plant | Draksavaleha | Formulation | IS_INGREDIENT_IN | {"match_type":"synonym_contains","matched_synonym":"Dhoney","pharmacopoeia_ref":"Madhu(-)","quantity":"768 g","source_db":"Ayurvedic Standard Treatment Guidelines; Ayurvedic Pharmacopeia of India"} |
| Coriandrum sativum | Plant | Lohasava | Formulation | IS_INGREDIENT_IN | {"match_type":"synonym_contains","matched_synonym":"Dhoney","pharmacopoeia_ref":"Madhu(-)","quantity":"3.072 kg","source_db":"Ayurvedic Standard Treatment Guidelines; Ayurvedic Pharmacopeia of India; Ayurvedic Formulary of India"} |
| Coriandrum sativum | Plant | Puga Khanda | Formulation | IS_INGREDIENT_IN | {"match_type":"synonym_exact","matched_synonym":"Coriandrum sativum","part":"Fruit","pharmacopoeia_ref":"Dhanyaka(Fruit)","quantity":"24 g","source_db":"Ayurvedic Pharmacopeia of India"} |
| Coriandrum sativum | Plant | Anemia | Disease | ASSOCIATED_WITH_DISEASE | {"DOID":"DOID_2355","ICD11":"3A9Z;3A00","MESH":"D000740","association_by_clinical_trials_of_plant_ingredients":"NCT01490944;NCT02780505;NCT02631668;NCT01670955;NCT02225886;NCT00920413;NCT00953134"} |
| Elettaria cardamomum | Plant | Talisadya Curna | Formulation | IS_INGREDIENT_IN | {"match_type":"synonym_exact","matched_synonym":"Elettaria cardamomum","part":"Seed","pharmacopoeia_ref":"Suksmaila(Fruit)","quantity":"6g","source_db":"Ayurvedic Pharmacopeia of India"} |
| Elettaria cardamomum | Plant | Puga Khanda | Formulation | IS_INGREDIENT_IN | {"match_type":"synonym_exact","matched_synonym":"Elettaria cardamomum","part":"Seed","pharmacopoeia_ref":"Suksmaila(Fruit)","quantity":"24 g","source_db":"Ayurvedic Pharmacopeia of India"} |
| Elettaria cardamomum | Plant | Pippalyadyasava | Formulation | IS_INGREDIENT_IN | {"match_type":"synonym_exact","matched_synonym":"Elettaria cardamomum","part":"Seed","pharmacopoeia_ref":"Suksmaila(Fruit)","quantity":"8g","source_db":"Ayurvedic Standard Treatment Guidelines; Ayurvedic Pharmacopeia of India; Ayurvedic Formulary of India"} |
| Elettaria cardamomum | Plant | Kalyanaka Ghrta | Formulation | IS_INGREDIENT_IN | {"match_type":"synonym_exact","matched_synonym":"Elettaria cardamomum","part":"Seed","pharmacopoeia_ref":"Suksmaila(Fruit)","quantity":"12 g","source_db":"Ayurvedic Standard Treatment Guidelines; Ayurvedic Pharmacopeia of India"} |
| Elettaria cardamomum | Plant | Draksasava | Formulation | IS_INGREDIENT_IN | {"match_type":"synonym_exact","matched_synonym":"Elettaria cardamomum","part":"Seed","pharmacopoeia_ref":"Suksmaila(Fruit)","quantity":"24 g","source_db":"Ayurvedic Standard Treatment Guidelines; Ayurvedic Pharmacopeia of India"} |
| Elettaria cardamomum | Plant | Khadirarista | Formulation | IS_INGREDIENT_IN | {"match_type":"synonym_exact","matched_synonym":"Elettaria cardamomum","part":"Seed","pharmacopoeia_ref":"Suksmaila(Fruit)","quantity":"48 g","source_db":"Ayurvedic Standard Treatment Guidelines; Ayurvedic Pharmacopeia of India; Ayurvedic Formulary of India"} |
| Elettaria cardamomum | Plant | Dasamularista | Formulation | IS_INGREDIENT_IN | {"match_type":"synonym_exact","matched_synonym":"Elettaria cardamomum","part":"Seed","pharmacopoeia_ref":"Suksmaila(Fruit)","quantity":"19 g","source_db":"Ayurvedic Standard Treatment Guidelines; Ayurvedic Pharmacopeia of India"} |
| Elettaria cardamomum | Plant | Anemia | Disease | ASSOCIATED_WITH_DISEASE | {"DOID":"DOID_2355","ICD11":"3A9Z;3A00","MESH":"D000740","association_by_clinical_trials_of_plant_ingredients":"NCT02780505;NCT02631668;NCT02225886;NCT00920413"} |
| Woodfordia fruticosa | Plant | Draksasava | Formulation | IS_INGREDIENT_IN | {"match_type":"synonym_exact","matched_synonym":"Woodfordia fruticosa","part":"Flower","pharmacopoeia_ref":"Dhataki(Flower)","quantity":"336 g","source_db":"Ayurvedic Standard Treatment Guidelines; Ayurvedic Pharmacopeia of India"} |
| Woodfordia fruticosa | Plant | Punarnavasava | Formulation | IS_INGREDIENT_IN | {"match_type":"synonym_exact","matched_synonym":"Woodfordia fruticosa","part":"Flower","pharmacopoeia_ref":"Dhataki(Flower)","quantity":"256 g","source_db":"Ayurvedic Standard Treatment Guidelines; Ayurvedic Pharmacopeia of India; Ayurvedic Formulary of India"} |
| Woodfordia fruticosa | Plant | Pippalyadyasava | Formulation | IS_INGREDIENT_IN | {"match_type":"synonym_exact","matched_synonym":"Woodfordia fruticosa","part":"Flower","pharmacopoeia_ref":"Dhataki(Flower)","quantity":"160 g","source_db":"Ayurvedic Standard Treatment Guidelines; Ayurvedic Pharmacopeia of India; Ayurvedic Formulary of India"} |
| Woodfordia fruticosa | Plant | Khadirarista | Formulation | IS_INGREDIENT_IN | {"match_type":"synonym_exact","matched_synonym":"Woodfordia fruticosa","part":"Flower","pharmacopoeia_ref":"Dhataki(Flower)","quantity":"960 g","source_db":"Ayurvedic Standard Treatment Guidelines; Ayurvedic Pharmacopeia of India; Ayurvedic Formulary of India"} |
| Woodfordia fruticosa | Plant | Dasamularista | Formulation | IS_INGREDIENT_IN | {"match_type":"synonym_exact","matched_synonym":"Woodfordia fruticosa","part":"Flower","pharmacopoeia_ref":"Dhataki(Flower)","quantity":"290 g","source_db":"Ayurvedic Standard Treatment Guidelines; Ayurvedic Pharmacopeia of India"} |
| Woodfordia fruticosa | Plant | Lohasava | Formulation | IS_INGREDIENT_IN | {"match_type":"synonym_exact","matched_synonym":"Woodfordia fruticosa","part":"Flower","pharmacopoeia_ref":"Dhataki(Flower)","quantity":"960 g","source_db":"Ayurvedic Standard Treatment Guidelines; Ayurvedic Pharmacopeia of India; Ayurvedic Formulary of India"} |
| Woodfordia fruticosa | Plant | Usirasava | Formulation | IS_INGREDIENT_IN | {"match_type":"synonym_exact","matched_synonym":"Woodfordia fruticosa","part":"Flower","pharmacopoeia_ref":"Dhataki(Flower)","quantity":"q.s. for dhupana","source_db":"Ayurvedic Standard Treatment Guidelines; Ayurvedic Pharmacopeia of India"} |
| Phyllanthus emblica | Plant | Guggulu Kaisora Guggulu | Formulation | IS_INGREDIENT_IN | {"match_type":"synonym_exact","matched_synonym":"Emblica officinalis","part":"Pericarp","pharmacopoeia_ref":"Amalaki(Fresh fruit)","quantity":"8g","source_db":"Ayurvedic Pharmacopeia of India"} |
| Phyllanthus emblica | Plant | Simhanada Guggulu | Formulation | IS_INGREDIENT_IN | {"match_type":"synonym_exact","matched_synonym":"Emblica officinalis","part":"Pericarp","pharmacopoeia_ref":"Amalaki(Fresh fruit)","quantity":"48 g","source_db":"Ayurvedic Standard Treatment Guidelines; Ayurvedic Pharmacopeia of India"} |
| Phyllanthus emblica | Plant | Kalyanaka Ghrta | Formulation | IS_INGREDIENT_IN | {"match_type":"synonym_exact","matched_synonym":"Emblica officinalis","part":"Pericarp","pharmacopoeia_ref":"Amalaki(Fresh fruit)","quantity":"12 g","source_db":"Ayurvedic Standard Treatment Guidelines; Ayurvedic Pharmacopeia of India"} |
| Phyllanthus emblica | Plant | Dasamularista | Formulation | IS_INGREDIENT_IN | {"match_type":"synonym_exact","matched_synonym":"Emblica officinalis","part":"Pericarp","pharmacopoeia_ref":"Amalaki(Fresh fruit)","quantity":"154 g","source_db":"Ayurvedic Standard Treatment Guidelines; Ayurvedic Pharmacopeia of India"} |
| Phyllanthus emblica | Plant | Navayasa Curna | Formulation | IS_INGREDIENT_IN | {"match_type":"synonym_exact","matched_synonym":"Emblica officinalis","part":"Pericarp","pharmacopoeia_ref":"Amalaki(Fresh fruit)","quantity":"1 part","source_db":"Ayurvedic Pharmacopeia of India"} |
| Phyllanthus emblica | Plant | Lohasava | Formulation | IS_INGREDIENT_IN | {"match_type":"synonym_exact","matched_synonym":"Emblica officinalis","part":"Pericarp","pharmacopoeia_ref":"Amalaki(Fresh fruit)","quantity":"192 g","source_db":"Ayurvedic Standard Treatment Guidelines; Ayurvedic Pharmacopeia of India; Ayurvedic Formulary of India"} |
| Phyllanthus emblica | Plant | Khadirarista | Formulation | IS_INGREDIENT_IN | {"match_type":"synonym_exact","matched_synonym":"Emblica officinalis","part":"Pericarp","pharmacopoeia_ref":"Amalaki(Fresh fruit)","quantity":"960 g","source_db":"Ayurvedic Standard Treatment Guidelines; Ayurvedic Pharmacopeia of India; Ayurvedic Formulary of India"} |
| Phyllanthus emblica | Plant | Draksavaleha | Formulation | IS_INGREDIENT_IN | {"match_type":"synonym_exact","matched_synonym":"Embelica officinalis","part":"Pericarp","pharmacopoeia_ref":"Amalaki(Fresh fruit)","quantity":"12.288 l","source_db":"Ayurvedic Standard Treatment Guidelines; Ayurvedic Pharmacopeia of India"} |
| Phyllanthus emblica | Plant | Anemia | Disease | ASSOCIATED_WITH_DISEASE | {"DOID":"DOID_2355","ICD11":"3A9Z;3A00","MESH":"D000740","association_by_clinical_trials_of_plant_ingredients":"NCT00920413;NCT02780505;NCT02631668;NCT02225886","part":"fruit","reference":"ISBN:9770972795006; ISBN:9780387706375; ISBN:9788171360536; ISBN:9788172361266; ISBN:9788173717055; Medicinal Plants Of Nagpur And Wardha Forest Divisions (Maharashtra); ISBN:9770972795006; ISBN:9788172360481; ISBN:9788172360818; ISBN:9788172362300"} |
| Choline | Phytochemical | Moringa oleifera | Plant | FOUND_IN | {"source_db":"cmaup_ingredients;fooddb_chem_all;npass_chem_all"} |
| Trigonelline | Phytochemical | Moringa oleifera | Plant | FOUND_IN | {"reference":"ISBN:9770972795006"} |
| Sucrose | Phytochemical | Moringa oleifera | Plant | FOUND_IN | {"reference":"ISBN:9788185042084"} |
| 3,4-Dihydroxybenzoic acid | Phytochemical | Moringa oleifera | Plant | FOUND_IN | {"reference":"ISBN:9788172362461"} |
| Luteolin | Phytochemical | Moringa oleifera | Plant | FOUND_IN | {"reference":"ISBN:9770972795006"} |
| p-Coumaric acid | Phytochemical | Moringa oleifera | Plant | FOUND_IN | {"reference":"ISBN:9770972795006"} |
| Apigenin | Phytochemical | Moringa oleifera | Plant | FOUND_IN | {"reference":"ISBN:9770972795006"} |
| Gallic Acid | Phytochemical | Moringa oleifera | Plant | FOUND_IN | {"reference":"ISBN:9770972795006"} |
| Rutin | Phytochemical | Moringa oleifera | Plant | FOUND_IN | {"reference":"ISBN:9770972795006"} |
| Stigmasterol | Phytochemical | Moringa oleifera | Plant | FOUND_IN | {"source_db":"fooddb_chem_all"} |
| DL-Xylose | Phytochemical | Moringa oleifera | Plant | FOUND_IN | {"source_db":"fooddb_chem_all"} |
| Aspartic Acid | Phytochemical | Moringa oleifera | Plant | FOUND_IN | {"source_db":"fooddb_chem_all"} |
| 2-Amino-3-methylpentanoic acid | Phytochemical | Moringa oleifera | Plant | FOUND_IN | {"source_db":"fooddb_chem_all"} |
| Sulfur | Phytochemical | Moringa oleifera | Plant | FOUND_IN | {"source_db":"fooddb_chem_all"} |
| Iron | Phytochemical | Moringa oleifera | Plant | FOUND_IN | {"source_db":"fooddb_chem_all"} |
| Potassium Cation | Phytochemical | Moringa oleifera | Plant | FOUND_IN | {"source_db":"fooddb_chem_all"} |
| Phosphine | Phytochemical | Moringa oleifera | Plant | FOUND_IN | {"source_db":"fooddb_chem_all"} |
| Glycine | Phytochemical | Moringa oleifera | Plant | FOUND_IN | {"source_db":"fooddb_chem_all"} |
| Alanine | Phytochemical | Moringa oleifera | Plant | FOUND_IN | {"source_db":"fooddb_chem_all"} |
| DL-leucine | Phytochemical | Moringa oleifera | Plant | FOUND_IN | {"source_db":"fooddb_chem_all"} |
| Arginine | Phytochemical | Moringa oleifera | Plant | FOUND_IN | {"source_db":"fooddb_chem_all"} |
| Methionine | Phytochemical | Moringa oleifera | Plant | FOUND_IN | {"source_db":"fooddb_chem_all"} |
| Cholest-5-en-3-ol | Phytochemical | Moringa oleifera | Plant | FOUND_IN | {"source_db":"fooddb_chem_all"} |
| DL-Histidine | Phytochemical | Moringa oleifera | Plant | FOUND_IN | {"source_db":"fooddb_chem_all"} |
| Copper | Phytochemical | Moringa oleifera | Plant | FOUND_IN | {"source_db":"fooddb_chem_all"} |
| Proline | Phytochemical | Moringa oleifera | Plant | FOUND_IN | {"source_db":"fooddb_chem_all"} |
| DL-Cystine | Phytochemical | Moringa oleifera | Plant | FOUND_IN | {"source_db":"fooddb_chem_all"} |
| Lysine | Phytochemical | Moringa oleifera | Plant | FOUND_IN | {"source_db":"fooddb_chem_all"} |
| Sodium | Phytochemical | Moringa oleifera | Plant | FOUND_IN | {"source_db":"fooddb_chem_all"} |
| Caffeic Acid | Phytochemical | Moringa oleifera | Plant | FOUND_IN | {"source_db":"fooddb_chem_all"} |
| DL-Tyrosine | Phytochemical | Moringa oleifera | Plant | FOUND_IN | {"source_db":"fooddb_chem_all"} |
| Threonine | Phytochemical | Moringa oleifera | Plant | FOUND_IN | {"source_db":"fooddb_chem_all"} |
| Tryptophan | Phytochemical | Moringa oleifera | Plant | FOUND_IN | {"source_db":"fooddb_chem_all"} |
| Elaidic Acid | Phytochemical | Moringa oleifera | Plant | FOUND_IN | {"source_db":"fooddb_chem_all"} |
| Calcium Cation | Phytochemical | Moringa oleifera | Plant | FOUND_IN | {"source_db":"fooddb_chem_all"} |
| Glutamic Acid | Phytochemical | Moringa oleifera | Plant | FOUND_IN | {"source_db":"fooddb_chem_all"} |
| Cyanidin 3-(6''-acetyl-galactoside) | Phytochemical | Moringa oleifera | Plant | FOUND_IN | {"source_db":"cmaup_ingredients"} |
| DL-Serine | Phytochemical | Moringa oleifera | Plant | FOUND_IN | {"source_db":"cmaup_ingredients;fooddb_chem_all"} |
| Ascorbic Acid | Phytochemical | Moringa oleifera | Plant | FOUND_IN | {"source_db":"cmaup_ingredients;fooddb_chem_all;npass_chem_all"} |
| Zeaxanthin | Phytochemical | Moringa oleifera | Plant | FOUND_IN | {"source_db":"cmaup_ingredients;npass_chem_all"} |
| Retinol | Phytochemical | Moringa oleifera | Plant | FOUND_IN | {"source_db":"cmaup_ingredients;npass_chem_all"} |
| Quercetin | Phytochemical | Moringa oleifera | Plant | FOUND_IN | {"source_db":"cmaup_ingredients;fooddb_chem_all;npass_chem_all"} |
| Thiamine | Phytochemical | Moringa oleifera | Plant | FOUND_IN | {"source_db":"cmaup_ingredients;npass_chem_all"} |
| Lutein | Phytochemical | Moringa oleifera | Plant | FOUND_IN | {"source_db":"cmaup_ingredients;npass_chem_all"} |
| Beta-Sitosterol | Phytochemical | Moringa oleifera | Plant | FOUND_IN | {"source_db":"cmaup_ingredients;fooddb_chem_all;npass_chem_all"} |
| Pyridoxine | Phytochemical | Moringa oleifera | Plant | FOUND_IN | {"source_db":"cmaup_ingredients;fooddb_chem_all;npass_chem_all"} |
| Nicotinic acid | Phytochemical | Moringa oleifera | Plant | FOUND_IN | {"source_db":"cmaup_ingredients;fooddb_chem_all;npass_chem_all"} |
| Kaempferol | Phytochemical | Moringa oleifera | Plant | FOUND_IN | {"source_db":"cmaup_ingredients;fooddb_chem_all;npass_chem_all"} |
| Ferulic acid | Phytochemical | Moringa oleifera | Plant | FOUND_IN | {"source_db":"cmaup_ingredients;npass_chem_all"} |
| Riboflavin | Phytochemical | Moringa oleifera | Plant | FOUND_IN | {"source_db":"cmaup_ingredients;fooddb_chem_all;npass_chem_all"} |
| all-trans-Neoxanthin | Phytochemical | Moringa oleifera | Plant | FOUND_IN | {"source_db":"cmaup_ingredients;npass_chem_all"} |
| Beta-Carotene | Phytochemical | Moringa oleifera | Plant | FOUND_IN | {"source_db":"cmaup_ingredients;fooddb_chem_all;npass_chem_all"} |
| Moringa oleifera | Plant | Prabhanjana Vimardana Taila | Formulation | IS_INGREDIENT_IN | {"match_type":"synonym_exact","matched_synonym":"Moringa oleifera","part":"Stem bark","pharmacopoeia_ref":"Sigru(Stem bark)","quantity":"25.6 g","source_db":"Ayurvedic Standard Treatment Guidelines; Ayurvedic Pharmacopeia of India"} |
| Moringa oleifera | Plant | Arthritis | Disease | ASSOCIATED_WITH_DISEASE | {"DOID":"DOID_848","ICD11":"FA2Z","MESH":"D001168","part":"flower; fruit; leaf; root; seed","reference":"ISBN:9770972795006; Standardization of Single Drugs of Unani Medicine Part - IV"} |
| Moringa oleifera | Plant | Muscular Dystrophies | Disease | ASSOCIATED_WITH_DISEASE | {"DOID":"DOID_9884","ICD11":"8C70","MESH":"D009136","association_by_therapeutic_target":"NFKB1;RELA"} |
| Moringa oleifera | Plant | Urinary Tract Infections | Disease | ASSOCIATED_WITH_DISEASE | {"DOID":"DOID_0080784","ICD11":"GC08","MESH":"D014552","association_by_clinical_trials_of_plant_ingredients":"NCT04261036;NCT03800667","association_by_therapeutic_target":"TOP2A"} |
| Moringa oleifera | Plant | Obesity | Disease | ASSOCIATED_WITH_DISEASE | {"DOID":"DOID_9970","ICD11":"5B81;5B80","MESH":"D009765;D019440","association_by_clinical_trials_of_plant_ingredients":"NCT01083329;NCT02612259;NCT05219799","association_by_therapeutic_target":"GRM3;GRM2;CHRM1"} |
| Moringa oleifera | Plant | Thrombocytopenia | Disease | ASSOCIATED_WITH_DISEASE | {"DOID":"DOID_1588","ICD11":"3B64","MESH":"D013921","association_by_clinical_trials_of_plant_ingredients":"NCT01014546;NCT00272610;NCT05025488","association_by_therapeutic_target":"F2;AHR;KDR;ALOX5"} |
| Moringa oleifera | Plant | Urinary Bladder Neoplasms | Disease | ASSOCIATED_WITH_DISEASE | {"DOID":"DOID_11054","ICD11":"2C94.Z","MESH":"D001749","association_by_clinical_trials_of_plant_ingredients":"NCT04046094;NCT03359239"} |
| Moringa oleifera | Plant | Neoplasms | Disease | ASSOCIATED_WITH_DISEASE | {"DOID":"DOID_162","ICD11":"2","MESH":"D009369","association_by_clinical_trials_of_plant_ingredients":"NCT01057589;NCT00024310;NCT01920191;NCT01191216;NCT00567931;NCT01324115;NCT00845689","part":"bark; fruit; leaf; root","reference":"ISBN:9770972795006; ISBN:9788173717055"} |
| Moringa oleifera | Plant | Muscular Disease | Disease | ASSOCIATED_WITH_DISEASE | {"DOID":"DOID_0080000","ICD11":"FB3Z","MESH":"D009135","association_by_clinical_trials_of_plant_ingredients":"NCT03223519"} |
| Moringa oleifera | Plant | Nose Diseases | Disease | ASSOCIATED_WITH_DISEASE | {"DOID":"DOID_2825","ICD11":"CA0Z","MESH":"D009668","association_by_clinical_trials_of_plant_ingredients":"NCT02730364;NCT02678234"} |
| Moringa oleifera | Plant | Uterine Cervical Neoplasms | Disease | ASSOCIATED_WITH_DISEASE | {"DOID":"DOID_4362","ICD11":"2E66","MESH":"D002583","association_by_clinical_trials_of_plant_ingredients":"NCT01715597"} |
| Moringa oleifera | Plant | Bronchitis | Disease | ASSOCIATED_WITH_DISEASE | {"DOID":"DOID_6132","ICD11":"CA20","MESH":"D001991","part":"root","reference":"ISBN:9788171360536; ISBN:9788173717055"} |
| Moringa oleifera | Plant | Testicular Diseases | Disease | ASSOCIATED_WITH_DISEASE | {"DOID":"DOID_2519","ICD11":"GB0Y","MESH":"D013733;D014555","part":"plant exudate; root","reference":"ISBN:9770972795006; ISBN:9788173717055"} |
| Moringa oleifera | Plant | Cholestasis | Disease | ASSOCIATED_WITH_DISEASE | {"DOID":"DOID_13580","ICD11":"DC10.02","MESH":"D002779","association_by_clinical_trials_of_plant_ingredients":"NCT03418935"} |
| Moringa oleifera | Plant | Smallpox | Disease | ASSOCIATED_WITH_DISEASE | {"DOID":"DOID_8736","ICD11":"1E70","MESH":"D012899","part":"bark; flower; fruit; leaf; root","reference":"ISBN:9770972795006"} |
| Moringa oleifera | Plant | Celiac Disease | Disease | ASSOCIATED_WITH_DISEASE | {"DOID":"DOID_10608","ICD11":"DA95","MESH":"D002446","association_by_disease_transcriptome_reversion":"HCAR2;CYP1A1;CDK1;TSHR"} |
| Moringa oleifera | Plant | Liver Neoplasms | Disease | ASSOCIATED_WITH_DISEASE | {"DOID":"DOID_3571","ICD11":"2C12","MESH":"D008113","association_by_therapeutic_target":"ADORA3;EGFR;IGF1R;MET;CSNK2A1;KDR;TOP2A"} |
| Moringa oleifera | Plant | Malaria | Disease | ASSOCIATED_WITH_DISEASE | {"DOID":"DOID_12365","ICD11":"1F45;1F40","MESH":"D008288","association_by_clinical_trials_of_plant_ingredients":"NCT02699099;NCT00616304;NCT02878265;NCT00147368;NCT00120822","association_by_therapeutic_target":"ADORA3","part":"bark","reference":"ISBN:9770972795006"} |
| Moringa oleifera | Plant | Asthma | Disease | ASSOCIATED_WITH_DISEASE | {"DOID":"DOID_2841","ICD11":"CA23","MESH":"D001249","association_by_clinical_trials_of_plant_ingredients":"NCT01501942;NCT00280683;NCT01057615","association_by_therapeutic_target":"CHRNA7;ALOX5;TOP2A;F2;CHRM5;CHRM3;CHRM4;CHRFAM7A;CHRM2;CA4","part":"leaf; root; seed","reference":"ISBN:9770972795006; ISBN:9770972795006; ISBN:9788171360536; ISBN:9788173717055; ISBN:9788172363130"} |
| Moringa oleifera | Plant | Epilepsy | Disease | ASSOCIATED_WITH_DISEASE | {"DOID":"DOID_1826","ICD11":"8A6Z","MESH":"D004827","part":"fruit; leaf; plant exudate; root; seed","reference":"ISBN:9770972795006; ISBN:9770972795006; ISBN:9788171360536; ISBN:9788172361266; ISBN:9788173717055"} |
| Moringa oleifera | Plant | Cataract | Disease | ASSOCIATED_WITH_DISEASE | {"DOID":"DOID_83","ICD11":"9B10","MESH":"D002386","association_by_clinical_trials_of_plant_ingredients":"NCT00000161","part":"leaf","reference":"ISBN:9770972795006"} |
| Moringa oleifera | Plant | Pre-Eclampsia | Disease | ASSOCIATED_WITH_DISEASE | {"DOID":"DOID_10591","ICD11":"JA24","MESH":"D011225","association_by_clinical_trials_of_plant_ingredients":"NCT03451266;NCT00571766;NCT00388856;NCT00974714;NCT00157521","association_by_disease_transcriptome_reversion":"ADORA2A;CA4","part":"seed","reference":"ISBN:9770972795006"} |
| Moringa oleifera | Plant | Psychotic Disorder | Disease | ASSOCIATED_WITH_DISEASE | {"DOID":"DOID_5419","ICD11":"6A20;6A25","MESH":"D012559","association_by_therapeutic_target":"GRM2;GRM3;DRD1"} |
| Moringa oleifera | Plant | Inflammatory Bowel Diseases | Disease | ASSOCIATED_WITH_DISEASE | {"DOID":"DOID_0050589","ICD11":"DD7Z","MESH":"D015212","association_by_disease_transcriptome_reversion":"NOX4;HCAR2;GRIN3A"} |
| Moringa oleifera | Plant | Scleroderma, Systemic | Disease | ASSOCIATED_WITH_DISEASE | {"DOID":"DOID_418","ICD11":"4A42","MESH":"D012595","association_by_disease_transcriptome_reversion":"NOX4;FABP4"} |
| Moringa oleifera | Plant | Osteoarthritis | Disease | ASSOCIATED_WITH_DISEASE | {"DOID":"DOID_8398","ICD11":"FA0Z","MESH":"D010003","association_by_disease_transcriptome_reversion":"TOP2A;GRM3;CA12;GRIA2;CDK1;GRIK1;HSD17B2"} |
| Moringa oleifera | Plant | Diabetes Mellitus | Disease | ASSOCIATED_WITH_DISEASE | {"DOID":"DOID_9351","ICD11":"5A14","MESH":"D003920","part":"aerial part; bark; fruit; leaf; seed","reference":"ISBN:9770972795006; Standardization of Single Drugs of Unani Medicine Part - IV"} |
| Moringa oleifera | Plant | Carotid Stenosis | Disease | ASSOCIATED_WITH_DISEASE | {"DOID":"DOID_13001","ICD11":"BD55","MESH":"D016893","association_by_clinical_trials_of_plant_ingredients":"NCT00000599;NCT00000600"} |
| Moringa oleifera | Plant | Lupus Erythematosus, Systemic | Disease | ASSOCIATED_WITH_DISEASE | {"DOID":"DOID_9074","ICD11":"4A40.0","MESH":"D008180","association_by_clinical_trials_of_plant_ingredients":"NCT00054938;NCT00470522","association_by_disease_transcriptome_reversion":"SLC1A1;ACHE;CHRM5;YES1;CYP1A1;ADRA2B"} |
| Moringa oleifera | Plant | Glaucoma | Disease | ASSOCIATED_WITH_DISEASE | {"DOID":"DOID_1686","ICD11":"9C61","MESH":"D005901","association_by_clinical_trials_of_plant_ingredients":"NCT03959592","association_by_therapeutic_target":"ADORA1;CHRM3;CHRM1;CHRM4;CHRM5;CHRM2;ADORA2A;CA2;ACHE;CA4;MET"} |
| Moringa oleifera | Plant | Anorexia | Disease | ASSOCIATED_WITH_DISEASE | {"DOID":"DOID_8689","ICD11":"MG43.7","MESH":"D000855","association_by_clinical_trials_of_plant_ingredients":"NCT01295450","part":"fruit; root","reference":"Standardization of Single Drugs of Unani Medicine Part - IV; ISBN:9788171360536; ISBN:9788173717055"} |
| Moringa oleifera | Plant | Alzheimer'S Disease 1 | Disease | ASSOCIATED_WITH_DISEASE | {"DOID":"DOID_0080348","ICD11":"8A20","MESH":"D000544","association_by_clinical_trials_of_plant_ingredients":"NCT00056225;NCT01439555;NCT04740580","association_by_therapeutic_target":"CHRNA7;TOP2A;GRM2;CHRFAM7A;GRM5;SNCA;MET;APP;GRM3;FLT3;KDR;CHRM1;ACHE;CHRNA4","part":"leaf","reference":"ISBN:9770972795006"} |
| Moringa oleifera | Plant | Psoriasis | Disease | ASSOCIATED_WITH_DISEASE | {"DOID":"DOID_8893","ICD11":"EA90","MESH":"D011565","association_by_disease_transcriptome_reversion":"FABP5","association_by_therapeutic_target":"DPP4;NFKB1;RELA;AHR;TOP2A;FLT3;ADORA3;RORC;ALOX5"} |
| Moringa oleifera | Plant | Cholera | Disease | ASSOCIATED_WITH_DISEASE | {"DOID":"DOID_1498","ICD11":"1A00;1C4Z","MESH":"D002771","association_by_therapeutic_target":"GRIN1;TOP2A;CYP2C9;CA4;CA12;CA2"} |
| Moringa oleifera | Plant | Lymphoma | Disease | ASSOCIATED_WITH_DISEASE | {"DOID":"DOID_0060058","ICD11":"2B33.5","MESH":"D008223","association_by_clinical_trials_of_plant_ingredients":"NCT00626444;NCT02702492"} |
| Moringa oleifera | Plant | Coronary Artery Disease | Disease | ASSOCIATED_WITH_DISEASE | {"DOID":"DOID_3393","ICD11":"BA8Z","MESH":"D003324","association_by_clinical_trials_of_plant_ingredients":"NCT00612521"} |
| Moringa oleifera | Plant | Dementia | Disease | ASSOCIATED_WITH_DISEASE | {"DOID":"DOID_1307","ICD11":"6D80;6D8Z","MESH":"D003704","association_by_therapeutic_target":"DRD1;CHRM1;ACHE"} |
| Moringa oleifera | Plant | Influenza, Human | Disease | ASSOCIATED_WITH_DISEASE | {"DOID":"DOID_8469","ICD11":"1E30;1E32","MESH":"D007251","association_by_clinical_trials_of_plant_ingredients":"NCT02735707","association_by_therapeutic_target":"GLO1;ALOX5;XDH"} |
| Moringa oleifera | Plant | Diabetes Mellitus, Type 1 | Disease | ASSOCIATED_WITH_DISEASE | {"DOID":"DOID_9744","ICD11":"5A10","MESH":"D003922","association_by_clinical_trials_of_plant_ingredients":"NCT02109315;NCT02730949"} |
| Moringa oleifera | Plant | Dental Caries | Disease | ASSOCIATED_WITH_DISEASE | {"DOID":"DOID_216","ICD11":"DA08.0","MESH":"D003731","association_by_clinical_trials_of_plant_ingredients":"NCT04750902;NCT02388178","part":"bark; plant exudate","reference":"ISBN:9770972795006; ISBN:9770972795006; ISBN:9788172363130"} |
| Moringa oleifera | Plant | Dysentery | Disease | ASSOCIATED_WITH_DISEASE | {"DOID":"DOID_13250","ICD11":"ME05.1","MESH":"D003967","part":"bark; fruit; leaf; root; stem","reference":"ISBN:9770972795006; ISBN:9770972795006; ISBN:9788171360536; ISBN:9788173717055"} |
| Moringa oleifera | Plant | Arthritis, Rheumatoid | Disease | ASSOCIATED_WITH_DISEASE | {"DOID":"DOID_7148","ICD11":"FA20","MESH":"D001172","association_by_clinical_trials_of_plant":"NCT05665985","association_by_clinical_trials_of_plant_ingredients":"NCT02930343;NCT00299104;NCT02379091;NCT00299130;NCT02093026;NCT01116141;NCT00266227;NCT01217814;NCT02393378;NCT01061736;NCT02293902;NCT02504671;NCT02833350;NCT01711359;NCT03172325;NCT01010581;NCT05363917;NCT00298272;NCT02795299;NCT03028467;NCT00950989;NCT04535427;NCT00243412;NCT01850680;NCT02467504;NCT02762838;NCT00578305;NCT00965653;NCT01873443","association_by_therapeutic_target":"RELA;ABCG2;ALOX5;NFKB1;ADORA3;CA2"} |
| Moringa oleifera | Plant | Myocardial Infarction | Disease | ASSOCIATED_WITH_DISEASE | {"DOID":"DOID_5844","ICD11":"BA41;BA43","MESH":"D009203","association_by_clinical_trials_of_plant_ingredients":"NCT00000541;NCT03516903;NCT00120289;NCT00000482;NCT00590070","association_by_therapeutic_target":"DPP4;F2"} |
| Moringa oleifera | Plant | Osteoporosis | Disease | ASSOCIATED_WITH_DISEASE | {"DOID":"DOID_11476","ICD11":"FB83.1","MESH":"D010024","association_by_clinical_trials_of_plant":"NCT03026660","association_by_clinical_trials_of_plant_ingredients":"NCT00330096"} |
| Moringa oleifera | Plant | Infertility, Male | Disease | ASSOCIATED_WITH_DISEASE | {"DOID":"DOID_12336","ICD11":"GB04","MESH":"D007248","association_by_clinical_trials_of_plant_ingredients":"NCT01407432;NCT00406874"} |
| Moringa oleifera | Plant | Cardiovascular Diseases | Disease | ASSOCIATED_WITH_DISEASE | {"DOID":"DOID_1287","ICD11":"BA00;BE2Z","MESH":"D002318","association_by_clinical_trials_of_plant_ingredients":"NCT00408577;NCT03679780","association_by_therapeutic_target":"SRC;HCAR2;MET;KDR;F2;PPARA"} |
| Moringa oleifera | Plant | Anemia | Disease | ASSOCIATED_WITH_DISEASE | {"DOID":"DOID_2355","ICD11":"3A9Z;3A00","MESH":"D000740","association_by_clinical_trials_of_plant_ingredients":"NCT02225886;NCT00920413;NCT02631668;NCT02780505;NCT01490944;NCT00953134;NCT03446612;NCT01670955","part":"fruit; leaf","reference":"ISBN:9770972795006"} |
| Moringa oleifera | Plant | Alopecia | Disease | ASSOCIATED_WITH_DISEASE | {"DOID":"DOID_987","ICD11":"ED70","MESH":"D000505","part":"flower; leaf","reference":"ISBN:9770972795006"} |
| Moringa oleifera | Plant | Skin Diseases | Disease | ASSOCIATED_WITH_DISEASE | {"DOID":"DOID_37","ICD11":"EM0Z","MESH":"D012871","part":"bark; leaf; root; seed","reference":"ISBN:9770972795006"} |
| Moringa oleifera | Plant | Adenocarcinoma | Disease | ASSOCIATED_WITH_DISEASE | {"DOID":"DOID_299","ICD11":"2D40","MESH":"D000230","association_by_clinical_trials_of_plant_ingredients":"NCT03908333;NCT03697239;NCT03410030"} |
| Moringa oleifera | Plant | Filariasis | Disease | ASSOCIATED_WITH_DISEASE | {"DOID":"DOID_1080","ICD11":"1F66","MESH":"D005368","association_by_therapeutic_target":"ALOX5","part":"bark","reference":"ISBN:9770972795006; ISBN:9788172362461"} |
| Moringa oleifera | Plant | Esophagitis | Disease | ASSOCIATED_WITH_DISEASE | {"DOID":"DOID_11963","ICD11":"DA24","MESH":"D004941","association_by_therapeutic_target":"CYP19A1"} |
| Moringa oleifera | Plant | Arthritis, Gouty | Disease | ASSOCIATED_WITH_DISEASE | {"DOID":"DOID_13189","ICD11":"FA25","MESH":"D006073","association_by_therapeutic_target":"PPARA;XDH","part":"bark; leaf; root; seed","reference":"ISBN:9770972795006; ISBN:9770972795006; Standardization of Single Drugs of Unani Medicine Part - IV; ISBN:9788172363130"} |
| Moringa oleifera | Plant | Crohn Disease | Disease | ASSOCIATED_WITH_DISEASE | {"DOID":"DOID_8778","ICD11":"DD70","MESH":"D003424","association_by_clinical_trials_of_plant_ingredients":"NCT00275418","association_by_disease_transcriptome_reversion":"HCAR2;CHRM4;GRIN3A","association_by_therapeutic_target":"RORC"} |
| Moringa oleifera | Plant | Splenic Diseases | Disease | ASSOCIATED_WITH_DISEASE | {"DOID":"DOID_2529","ICD11":"3B8Z","MESH":"D013158","part":"bark; flower; fruit; leaf; root; seed","reference":"ISBN:9770972795006; ISBN:9770972795006; ISBN:9788172361266; ISBN:9788172363130"} |
| Moringa oleifera | Plant | Stomatitis | Disease | ASSOCIATED_WITH_DISEASE | {"DOID":"DOID_9637","ICD11":"DA01","MESH":"D013280","association_by_clinical_trials_of_plant_ingredients":"NCT03581773;NCT02868151"} |
| Moringa oleifera | Plant | Cystic Fibrosis | Disease | ASSOCIATED_WITH_DISEASE | {"DOID":"DOID_1485","ICD11":"CA25","MESH":"D003550","association_by_clinical_trials_of_plant_ingredients":"NCT01417481;NCT00405665;NCT01348204"} |
| Stigmasterol | Phytochemical | Trigonella foenum-graecum | Plant | FOUND_IN | {"source_db":"fooddb_chem_all"} |
| DL-Xylose | Phytochemical | Trigonella foenum-graecum | Plant | FOUND_IN | {"source_db":"fooddb_chem_all"} |
| Aspartic Acid | Phytochemical | Trigonella foenum-graecum | Plant | FOUND_IN | {"source_db":"fooddb_chem_all"} |
| 2-Amino-3-methylpentanoic acid | Phytochemical | Trigonella foenum-graecum | Plant | FOUND_IN | {"source_db":"fooddb_chem_all"} |
| Sulfur | Phytochemical | Trigonella foenum-graecum | Plant | FOUND_IN | {"source_db":"fooddb_chem_all"} |
| Kaempferol | Phytochemical | Trigonella foenum-graecum | Plant | FOUND_IN | {"source_db":"fooddb_chem_all"} |
| Phosphine | Phytochemical | Trigonella foenum-graecum | Plant | FOUND_IN | {"source_db":"fooddb_chem_all"} |
| Potassium Cation | Phytochemical | Trigonella foenum-graecum | Plant | FOUND_IN | {"source_db":"fooddb_chem_all"} |
| Iron | Phytochemical | Trigonella foenum-graecum | Plant | FOUND_IN | {"source_db":"fooddb_chem_all"} |
| Beta-Sitosterol | Phytochemical | Trigonella foenum-graecum | Plant | FOUND_IN | {"source_db":"fooddb_chem_all"} |
| Glycine | Phytochemical | Trigonella foenum-graecum | Plant | FOUND_IN | {"source_db":"fooddb_chem_all"} |
| Apigenin | Phytochemical | Trigonella foenum-graecum | Plant | FOUND_IN | {"source_db":"fooddb_chem_all"} |
| DL-leucine | Phytochemical | Trigonella foenum-graecum | Plant | FOUND_IN | {"source_db":"fooddb_chem_all"} |
| Alanine | Phytochemical | Trigonella foenum-graecum | Plant | FOUND_IN | {"source_db":"fooddb_chem_all"} |
| Arginine | Phytochemical | Trigonella foenum-graecum | Plant | FOUND_IN | {"source_db":"fooddb_chem_all"} |
| Methionine | Phytochemical | Trigonella foenum-graecum | Plant | FOUND_IN | {"source_db":"fooddb_chem_all"} |
| Cholest-5-en-3-ol | Phytochemical | Trigonella foenum-graecum | Plant | FOUND_IN | {"source_db":"fooddb_chem_all"} |
| Lysine | Phytochemical | Trigonella foenum-graecum | Plant | FOUND_IN | {"source_db":"fooddb_chem_all"} |
| Proline | Phytochemical | Trigonella foenum-graecum | Plant | FOUND_IN | {"source_db":"fooddb_chem_all"} |
| DL-Cystine | Phytochemical | Trigonella foenum-graecum | Plant | FOUND_IN | {"source_db":"fooddb_chem_all"} |
| Sodium | Phytochemical | Trigonella foenum-graecum | Plant | FOUND_IN | {"source_db":"fooddb_chem_all"} |
| p-Coumaric acid | Phytochemical | Trigonella foenum-graecum | Plant | FOUND_IN | {"source_db":"fooddb_chem_all"} |
| Copper | Phytochemical | Trigonella foenum-graecum | Plant | FOUND_IN | {"source_db":"fooddb_chem_all"} |
| DL-Histidine | Phytochemical | Trigonella foenum-graecum | Plant | FOUND_IN | {"source_db":"fooddb_chem_all"} |
| Tryptophan | Phytochemical | Trigonella foenum-graecum | Plant | FOUND_IN | {"source_db":"fooddb_chem_all"} |
| Luteolin | Phytochemical | Trigonella foenum-graecum | Plant | FOUND_IN | {"source_db":"fooddb_chem_all"} |
| Threonine | Phytochemical | Trigonella foenum-graecum | Plant | FOUND_IN | {"source_db":"fooddb_chem_all"} |
| DL-Tyrosine | Phytochemical | Trigonella foenum-graecum | Plant | FOUND_IN | {"source_db":"fooddb_chem_all"} |
| Elaidic Acid | Phytochemical | Trigonella foenum-graecum | Plant | FOUND_IN | {"source_db":"fooddb_chem_all"} |
| Calcium Cation | Phytochemical | Trigonella foenum-graecum | Plant | FOUND_IN | {"source_db":"fooddb_chem_all"} |
| Glutamic Acid | Phytochemical | Trigonella foenum-graecum | Plant | FOUND_IN | {"source_db":"fooddb_chem_all"} |
| Sucrose | Phytochemical | Trigonella foenum-graecum | Plant | FOUND_IN | {"source_db":"fooddb_chem_all"} |
| Cyanidin 3-(6''-acetyl-galactoside) | Phytochemical | Trigonella foenum-graecum | Plant | FOUND_IN | {"source_db":"cmaup_ingredients"} |
| DL-Serine | Phytochemical | Trigonella foenum-graecum | Plant | FOUND_IN | {"source_db":"cmaup_ingredients;fooddb_chem_all"} |
| Ascorbic Acid | Phytochemical | Trigonella foenum-graecum | Plant | FOUND_IN | {"source_db":"cmaup_ingredients;fooddb_chem_all;npass_chem_all"} |
| Gallic Acid | Phytochemical | Trigonella foenum-graecum | Plant | FOUND_IN | {"source_db":"cmaup_ingredients;npass_chem_all"} |
| Zeaxanthin | Phytochemical | Trigonella foenum-graecum | Plant | FOUND_IN | {"source_db":"cmaup_ingredients;npass_chem_all"} |
| Retinol | Phytochemical | Trigonella foenum-graecum | Plant | FOUND_IN | {"source_db":"cmaup_ingredients;npass_chem_all"} |
| Caffeic Acid | Phytochemical | Trigonella foenum-graecum | Plant | FOUND_IN | {"source_db":"cmaup_ingredients;npass_chem_all"} |
| Trigonelline | Phytochemical | Trigonella foenum-graecum | Plant | FOUND_IN | {"source_db":"cmaup_ingredients;fooddb_chem_all;npass_chem_all"} |
| Pyridoxine | Phytochemical | Trigonella foenum-graecum | Plant | FOUND_IN | {"source_db":"cmaup_ingredients;fooddb_chem_all;npass_chem_all"} |
| Rutin | Phytochemical | Trigonella foenum-graecum | Plant | FOUND_IN | {"source_db":"cmaup_ingredients;fooddb_chem_all;npass_chem_all"} |
| Nicotinic acid | Phytochemical | Trigonella foenum-graecum | Plant | FOUND_IN | {"source_db":"cmaup_ingredients;fooddb_chem_all;npass_chem_all"} |
| Ferulic acid | Phytochemical | Trigonella foenum-graecum | Plant | FOUND_IN | {"source_db":"cmaup_ingredients;npass_chem_all"} |
| Riboflavin | Phytochemical | Trigonella foenum-graecum | Plant | FOUND_IN | {"source_db":"cmaup_ingredients;fooddb_chem_all;npass_chem_all"} |
| all-trans-Neoxanthin | Phytochemical | Trigonella foenum-graecum | Plant | FOUND_IN | {"source_db":"cmaup_ingredients;npass_chem_all"} |
| Beta-Carotene | Phytochemical | Trigonella foenum-graecum | Plant | FOUND_IN | {"source_db":"cmaup_ingredients;fooddb_chem_all;npass_chem_all"} |
| Choline | Phytochemical | Trigonella foenum-graecum | Plant | FOUND_IN | {"source_db":"cmaup_ingredients;fooddb_chem_all;npass_chem_all"} |
| Lutein | Phytochemical | Trigonella foenum-graecum | Plant | FOUND_IN | {"source_db":"cmaup_ingredients;fooddb_chem_all;npass_chem_all"} |
| 3,4-Dihydroxybenzoic acid | Phytochemical | Trigonella foenum-graecum | Plant | FOUND_IN | {"source_db":"cmaup_ingredients;npass_chem_all"} |
| Thiamine | Phytochemical | Trigonella foenum-graecum | Plant | FOUND_IN | {"source_db":"cmaup_ingredients;npass_chem_all"} |
| Quercetin | Phytochemical | Trigonella foenum-graecum | Plant | FOUND_IN | {"source_db":"cmaup_ingredients;fooddb_chem_all;npass_chem_all"} |
| Trigonella foenum-graecum | Plant | Prabhanjana Vimardana Taila | Formulation | IS_INGREDIENT_IN | {"match_type":"synonym_exact","matched_synonym":"Trigonella foenum-graecum","part":"Seed","pharmacopoeia_ref":"Methi(Seed)","quantity":"12 g","source_db":"Ayurvedic Standard Treatment Guidelines; Ayurvedic Pharmacopeia of India"} |
| Trigonella foenum-graecum | Plant | Asthma | Disease | ASSOCIATED_WITH_DISEASE | {"DOID":"DOID_2841","ICD11":"CA23","MESH":"D001249","association_by_clinical_trials_of_plant_ingredients":"NCT01057615;NCT00280683;NCT01501942","association_by_therapeutic_target":"ESRRB;ALOX5;ESRRA;CA4"} |
| Trigonella foenum-graecum | Plant | Arthritis, Rheumatoid | Disease | ASSOCIATED_WITH_DISEASE | {"DOID":"DOID_7148","ICD11":"FA20","MESH":"D001172","association_by_clinical_trials_of_plant_ingredients":"NCT00298272;NCT03172325;NCT02833350;NCT00578305;NCT05363917;NCT01850680;NCT01061736;NCT01873443;NCT00266227;NCT02467504;NCT02093026;NCT00299104;NCT02379091;NCT02293902;NCT02795299;NCT00299130;NCT02393378;NCT01711359;NCT00243412;NCT01010581;NCT00950989;NCT03028467;NCT01217814;NCT02930343;NCT04535427;NCT00965653;NCT02762838;NCT02504671;NCT01116141","association_by_therapeutic_target":"ALOX5;ABCG2;ESRRA;MMP1;NFKB1;CA2;MMP9"} |
| Trigonella foenum-graecum | Plant | Osteoarthritis | Disease | ASSOCIATED_WITH_DISEASE | {"DOID":"DOID_8398","ICD11":"FA0Z","MESH":"D010003","association_by_disease_transcriptome_reversion":"CA12;CDK1;MMP9;MMP2;GRIA2;GRIK1;GRM3"} |
| Trigonella foenum-graecum | Plant | Dementia | Disease | ASSOCIATED_WITH_DISEASE | {"DOID":"DOID_1307","ICD11":"6D80;6D8Z","MESH":"D003704","association_by_therapeutic_target":"MAOB;ACHE"} |
| Trigonella foenum-graecum | Plant | Dysentery | Disease | ASSOCIATED_WITH_DISEASE | {"DOID":["DOID_12384;DOID_100","DOID_13250"],"ICD11":["1A40.Z","ME05.1"],"MESH":["D004751;D007411;D004403;D003968","D003967"],"part":"seed","reference":["ISBN:9780387706375; ISBN:9788171360536; ISBN:9788172361266; ISBN:9789327275590; ISBN:9788172363178","ISBN:9780387706375; ISBN:9788171360536; ISBN:9789327275590; ISBN:9788172363178"]} |
| Trigonella foenum-graecum | Plant | Epilepsy | Disease | ASSOCIATED_WITH_DISEASE | {"DOID":"DOID_1826","ICD11":"8A6Z;8A61","MESH":"D004827","association_by_clinical_trials_of_plant_ingredients":"NCT02318446;NCT02369822;NCT00004758","association_by_therapeutic_target":"GRIK1;GRM2;GRIN2B"} |
| Trigonella foenum-graecum | Plant | Testicular Diseases | Disease | ASSOCIATED_WITH_DISEASE | {"DOID":"DOID_2519","ICD11":"GB0Y","MESH":"D013733;D014555","part":"seed","reference":"The Ayurvedic Pharmacopoeia of India Part-1 Volume-9"} |
| Trigonella foenum-graecum | Plant | Neoplasms | Disease | ASSOCIATED_WITH_DISEASE | {"DOID":"DOID_162","ICD11":"2E2Z;2D50","MESH":"D009369","association_by_therapeutic_target":"ESR1"} |
| Trigonella foenum-graecum | Plant | Scleroderma, Systemic | Disease | ASSOCIATED_WITH_DISEASE | {"DOID":"DOID_418","ICD11":"4A42","MESH":"D012595","association_by_disease_transcriptome_reversion":"NOX4;MMP1"} |
| Trigonella foenum-graecum | Plant | Filariasis | Disease | ASSOCIATED_WITH_DISEASE | {"DOID":"DOID_1080","ICD11":"1F66","MESH":"D005368","association_by_therapeutic_target":"ALOX5"} |
| Trigonella foenum-graecum | Plant | Stomatitis | Disease | ASSOCIATED_WITH_DISEASE | {"DOID":"DOID_9637","ICD11":"DA01","MESH":"D013280","association_by_clinical_trials_of_plant_ingredients":"NCT03581773;NCT02868151"} |
| Trigonella foenum-graecum | Plant | Pre-Eclampsia | Disease | ASSOCIATED_WITH_DISEASE | {"DOID":"DOID_10591","ICD11":"JA24","MESH":"D011225","association_by_clinical_trials_of_plant_ingredients":"NCT00974714;NCT00388856;NCT00571766;NCT03451266;NCT00157521","association_by_disease_transcriptome_reversion":"CA4"} |
| Trigonella foenum-graecum | Plant | Alzheimer'S Disease 1 | Disease | ASSOCIATED_WITH_DISEASE | {"DOID":"DOID_0080348","ICD11":"8A20","MESH":"D000544","association_by_clinical_trials_of_plant_ingredients":"NCT00056225;NCT04740580;NCT01439555;NCT03062449","association_by_therapeutic_target":"GRM3;ACHE;MAPT;SNCA;GRM5;MAOB;FLT3;GRM2;KDR;APP;ESR2;MET"} |
| Trigonella foenum-graecum | Plant | Esophagitis | Disease | ASSOCIATED_WITH_DISEASE | {"DOID":"DOID_11963","ICD11":"DA24","MESH":"D004941","association_by_therapeutic_target":"CYP19A1"} |
| Trigonella foenum-graecum | Plant | Osteoporosis | Disease | ASSOCIATED_WITH_DISEASE | {"DOID":"DOID_11476","ICD11":"FB83.1","MESH":"D010024","association_by_clinical_trials_of_plant_ingredients":"NCT00010686"} |
| Trigonella foenum-graecum | Plant | Arthritis | Disease | ASSOCIATED_WITH_DISEASE | {"DOID":"DOID_848","ICD11":"FA2Z","MESH":"D001168","association_by_therapeutic_target":"ESRRB;ESRRA;FLT3"} |
| Trigonella foenum-graecum | Plant | Anorexia | Disease | ASSOCIATED_WITH_DISEASE | {"DOID":"DOID_8689","ICD11":"MG43.7","MESH":"D000855","association_by_clinical_trials_of_plant_ingredients":"NCT01295450","part":"leaf; seed","reference":"ISBN:9788171360536; ISBN:9780387706375; ISBN:9788173717062; ISBN:9788172363178"} |
| Trigonella foenum-graecum | Plant | Nose Diseases | Disease | ASSOCIATED_WITH_DISEASE | {"DOID":"DOID_2825","ICD11":"CA0Z","MESH":"D009668","association_by_clinical_trials_of_plant_ingredients":"NCT02678234;NCT02730364"} |
| Trigonella foenum-graecum | Plant | Diabetes Mellitus | Disease | ASSOCIATED_WITH_DISEASE | {"DOID":"DOID_9351","ICD11":"5A14","MESH":"D003920","association_by_clinical_trials_of_plant":"NCT00597350","association_by_clinical_trials_of_plant_ingredients":"NCT02107976;NCT02297399","association_by_therapeutic_target":"ESR1;NFKB1;DPP4","part":"leaf; seed","reference":"ISBN:9788171360536; ISBN:9788172363093; Medicinal Plants Of Nagpur And Wardha Forest Divisions (Maharashtra); ISBN:9788172363178"} |
| Trigonella foenum-graecum | Plant | Anemia | Disease | ASSOCIATED_WITH_DISEASE | {"DOID":"DOID_2355","ICD11":"3A9Z;3A00","MESH":"D000740","association_by_clinical_trials_of_plant_ingredients":"NCT00920413;NCT02225886;NCT02631668;NCT02780505;NCT01670955;NCT01490944;NCT00953134","reference":"ISBN:9788172363178"} |
| Trigonella foenum-graecum | Plant | Glaucoma | Disease | ASSOCIATED_WITH_DISEASE | {"DOID":"DOID_1686","ICD11":"9C61","MESH":"D005901","association_by_clinical_trials_of_plant_ingredients":"NCT03959592","association_by_therapeutic_target":"CA4;MET;CA2;ACHE"} |
| Trigonella foenum-graecum | Plant | Cholera | Disease | ASSOCIATED_WITH_DISEASE | {"DOID":"DOID_1498","ICD11":["1A00;CA43","1A00;1C4Z"],"MESH":"D002771","association_by_therapeutic_target":["ESRRB;ESRRA;SRC;ALOX5","CYP2C9;GRIN1;CA12;CA4;CA2;CA14"]} |
| Trigonella foenum-graecum | Plant | Dental Caries | Disease | ASSOCIATED_WITH_DISEASE | {"DOID":"DOID_216","ICD11":"DA08.0","MESH":"D003731","association_by_clinical_trials_of_plant_ingredients":"NCT02388178;NCT04750902"} |
| Trigonella foenum-graecum | Plant | Muscular Dystrophies | Disease | ASSOCIATED_WITH_DISEASE | {"DOID":"DOID_9884","ICD11":"8C70","MESH":"D009136","association_by_therapeutic_target":"NFKB1;MAOB"} |
| Trigonella foenum-graecum | Plant | Thrombocytopenia | Disease | ASSOCIATED_WITH_DISEASE | {"DOID":"DOID_1588","ICD11":"3B64","MESH":"D013921","association_by_clinical_trials_of_plant_ingredients":"NCT05025488;NCT01014546;NCT00272610","association_by_therapeutic_target":"ALOX5;KDR"} |
| Trigonella foenum-graecum | Plant | Psychotic Disorder | Disease | ASSOCIATED_WITH_DISEASE | {"DOID":"DOID_5419","ICD11":"6A20;6A25","MESH":"D012559","association_by_therapeutic_target":"GRM3;GRM2"} |
| Trigonella foenum-graecum | Plant | Cholestasis | Disease | ASSOCIATED_WITH_DISEASE | {"DOID":"DOID_13580","ICD11":"DC10.02","MESH":"D002779","association_by_clinical_trials_of_plant_ingredients":"NCT03418935"} |
| Trigonella foenum-graecum | Plant | Infertility, Male | Disease | ASSOCIATED_WITH_DISEASE | {"DOID":"DOID_12336","ICD11":"GB04","MESH":"D007248","association_by_clinical_trials_of_plant_ingredients":"NCT00406874;NCT01407432","association_by_therapeutic_target":"ESR1"} |
| Trigonella foenum-graecum | Plant | Influenza, Human | Disease | ASSOCIATED_WITH_DISEASE | {"DOID":"DOID_8469","ICD11":"1E30;1E32","MESH":"D007251","association_by_clinical_trials_of_plant_ingredients":"NCT02735707","association_by_therapeutic_target":"ALOX5;XDH"} |
| Trigonella foenum-graecum | Plant | Obesity | Disease | ASSOCIATED_WITH_DISEASE | {"DOID":"DOID_9970","ICD11":"5B81;5B80","MESH":"D009765;D019440","association_by_clinical_trials_of_plant_ingredients":"NCT01083329;NCT02612259","association_by_therapeutic_target":"GRM2;GRM3"} |
| Trigonella foenum-graecum | Plant | Skin Diseases | Disease | ASSOCIATED_WITH_DISEASE | {"DOID":"DOID_37","ICD11":"EA00;EM0Z","MESH":"D012871","association_by_therapeutic_target":"ESRRA;ESRRB"} |
| Trigonella foenum-graecum | Plant | Urinary Bladder Neoplasms | Disease | ASSOCIATED_WITH_DISEASE | {"DOID":"DOID_11054","ICD11":"2C94.Z","MESH":"D001749","association_by_clinical_trials_of_plant_ingredients":"NCT04046094;NCT03359239;NCT01489813"} |
| Trigonella foenum-graecum | Plant | Cataract | Disease | ASSOCIATED_WITH_DISEASE | {"DOID":"DOID_83","ICD11":"9B10","MESH":"D002386","association_by_clinical_trials_of_plant_ingredients":"NCT00000161"} |
| Trigonella foenum-graecum | Plant | Lymphoma | Disease | ASSOCIATED_WITH_DISEASE | {"DOID":"DOID_0060058","ICD11":"2B33.5","MESH":"D008223","association_by_clinical_trials_of_plant_ingredients":"NCT00626444;NCT02702492"} |
| Trigonella foenum-graecum | Plant | Bronchitis | Disease | ASSOCIATED_WITH_DISEASE | {"DOID":"DOID_6132","ICD11":"CA20","MESH":"D001991","part":"leaf; seed","reference":"ISBN:9788171360536; ISBN:9788173717062"} |
| Trigonella foenum-graecum | Plant | Inflammatory Bowel Diseases | Disease | ASSOCIATED_WITH_DISEASE | {"DOID":"DOID_0050589","ICD11":"DD7Z","MESH":"D015212","association_by_disease_transcriptome_reversion":"MMP9;HCAR2;MMP1;GRIN3A;NOX4"} |
| Trigonella foenum-graecum | Plant | Adenocarcinoma | Disease | ASSOCIATED_WITH_DISEASE | {"DOID":"DOID_299","ICD11":"2D40","MESH":"D000230","association_by_clinical_trials_of_plant_ingredients":"NCT03908333;NCT01958372;NCT03410030;NCT03697239"} |
| Trigonella foenum-graecum | Plant | Celiac Disease | Disease | ASSOCIATED_WITH_DISEASE | {"DOID":"DOID_10608","ICD11":"DA95","MESH":"D002446","association_by_disease_transcriptome_reversion":"CDK1;HCAR2;TSHR;CYP1A1"} |
| Trigonella foenum-graecum | Plant | Myocardial Infarction | Disease | ASSOCIATED_WITH_DISEASE | {"DOID":"DOID_5844","ICD11":["BA41;BA43","BA41"],"MESH":"D009203","association_by_clinical_trials_of_plant_ingredients":["NCT00000541;NCT03516903;NCT00120289;NCT00000482","NCT02929888"],"association_by_therapeutic_target":"DPP4"} |
| Trigonella foenum-graecum | Plant | Coronary Artery Disease | Disease | ASSOCIATED_WITH_DISEASE | {"DOID":"DOID_3393","ICD11":"BA8Z;BA8Y","MESH":"D003324","association_by_clinical_trials_of_plant_ingredients":"NCT04907253;NCT03943459;NCT00134433;NCT00300352;NCT00287690"} |
| Trigonella foenum-graecum | Plant | Diabetes Mellitus, Type 1 | Disease | ASSOCIATED_WITH_DISEASE | {"DOID":"DOID_9744","ICD11":"5A10","MESH":"D003922","association_by_clinical_trials_of_plant_ingredients":"NCT02109315;NCT02730949"} |
| Trigonella foenum-graecum | Plant | Urinary Tract Infections | Disease | ASSOCIATED_WITH_DISEASE | {"DOID":"DOID_0080784","ICD11":"GC08","MESH":"D014552","association_by_clinical_trials_of_plant_ingredients":"NCT03800667;NCT04261036"} |
| Trigonella foenum-graecum | Plant | Carotid Stenosis | Disease | ASSOCIATED_WITH_DISEASE | {"DOID":"DOID_13001","ICD11":"BD55","MESH":"D016893","association_by_clinical_trials_of_plant_ingredients":"NCT00000600;NCT00000599"} |
| Trigonella foenum-graecum | Plant | Cardiovascular Diseases | Disease | ASSOCIATED_WITH_DISEASE | {"DOID":"DOID_1287","ICD11":"BA00;BE2Z","MESH":"D002318","association_by_clinical_trials_of_plant_ingredients":"NCT00408577;NCT03679780","association_by_therapeutic_target":"KDR;MET;SRC;HCAR2"} |
| Trigonella foenum-graecum | Plant | Smallpox | Disease | ASSOCIATED_WITH_DISEASE | {"DOID":"DOID_8736","ICD11":"1E70","MESH":"D012899","part":"seed","reference":"ISBN:9788171360536; ISBN:9788172361266; ISBN:9788173717062"} |
| Trigonella foenum-graecum | Plant | Arthritis, Gouty | Disease | ASSOCIATED_WITH_DISEASE | {"DOID":"DOID_13189","ICD11":"FA25","MESH":"D006073","association_by_therapeutic_target":"XDH"} |
| Trigonella foenum-graecum | Plant | Muscular Disease | Disease | ASSOCIATED_WITH_DISEASE | {"DOID":"DOID_0080000","ICD11":"FB3Z","MESH":"D009135","association_by_clinical_trials_of_plant_ingredients":"NCT03223519"} |
| Trigonella foenum-graecum | Plant | Psoriasis | Disease | ASSOCIATED_WITH_DISEASE | {"DOID":"DOID_8893","ICD11":"EA90","MESH":"D011565","association_by_therapeutic_target":"ESRRA;DPP4;ALOX5;NFKB1;ESRRB;FLT3"} |
| Trigonella foenum-graecum | Plant | Crohn Disease | Disease | ASSOCIATED_WITH_DISEASE | {"DOID":"DOID_8778","ICD11":"DD70","MESH":"D003424","association_by_clinical_trials_of_plant_ingredients":"NCT00275418","association_by_disease_transcriptome_reversion":"MMP9;MMP1;HCAR2;GRIN3A","association_by_therapeutic_target":"MMP9"} |
| Trigonella foenum-graecum | Plant | Liver Neoplasms | Disease | ASSOCIATED_WITH_DISEASE | {"DOID":"DOID_3571","ICD11":"2C12","MESH":"D008113","association_by_therapeutic_target":"MET;CSNK2A1;KDR;IGF1R;EGFR"} |
| Trigonella foenum-graecum | Plant | Cystic Fibrosis | Disease | ASSOCIATED_WITH_DISEASE | {"DOID":"DOID_1485","ICD11":"CA25","MESH":"D003550","association_by_clinical_trials_of_plant_ingredients":"NCT01417481;NCT00405665;NCT00590538;NCT00016744;NCT01348204"} |
| Trigonella foenum-graecum | Plant | Alopecia | Disease | ASSOCIATED_WITH_DISEASE | {"DOID":"DOID_987","ICD11":"ED70","MESH":"D000505","part":"seed","reference":"ISBN:9788172363093"} |
| Trigonella foenum-graecum | Plant | Uterine Cervical Neoplasms | Disease | ASSOCIATED_WITH_DISEASE | {"DOID":"DOID_4362","ICD11":"2E66","MESH":"D002583","association_by_clinical_trials_of_plant_ingredients":"NCT01715597"} |
| Trigonella foenum-graecum | Plant | Lupus Erythematosus, Systemic | Disease | ASSOCIATED_WITH_DISEASE | {"DOID":"DOID_9074","ICD11":"4A40.0","MESH":"D008180","association_by_clinical_trials_of_plant_ingredients":"NCT00470522;NCT00054938","association_by_disease_transcriptome_reversion":"MMP9;ACHE;ADRA2B;SLC1A1;YES1;CYP1A1;ESRRB"} |
| Trigonella foenum-graecum | Plant | Malaria | Disease | ASSOCIATED_WITH_DISEASE | {"DOID":"DOID_12365","ICD11":"1F4Z","MESH":"D008288","association_by_disease_transcriptome_reversion":"MMP9;HCAR2"} |
| Trigonella foenum-graecum | Plant | Splenic Diseases | Disease | ASSOCIATED_WITH_DISEASE | {"DOID":"DOID_2529","ICD11":"3B8Z","MESH":"D013158","part":"seed","reference":"ISBN:9789327275590; ISBN:9788172363178"} |
